# Autologous NK cells as consolidation therapy following stem cell transplantation in multiple myeloma

**DOI:** 10.1016/j.xcrm.2022.100508

**Published:** 2022-01-28

**Authors:** Hareth Nahi, Michael Chrobok, Stephan Meinke, Charlotte Gran, Nicole Marquardt, Gabriel Afram, Tolga Sutlu, Mari Gilljam, Birgitta Stellan, Arnika K. Wagner, Pontus Blomberg, Per-Henrik Holmqvist, Lilian Walther-Jallow, Karin Mellström, Johan Liwing, Charlotte Gustafsson, Robert Månsson, Monika Klimkowska, Gösta Gahrton, Johan Lund, Per Ljungman, Hans-Gustaf Ljunggren, Evren Alici

**Affiliations:** 1 Center for Hematology and Regenerative Medicine, Department of Medicine Huddinge, Karolinska Institutet, SE-14183 Huddinge, Sweden; 2 Department of Hematology, Karolinska University Hospital, SE-14186 Huddinge, Sweden; 3 Department of Clinical Chemistry, Karolinska University Laboratory, SE-14183 Huddinge, Sweden; 4 Center for Infectious Medicine, Department of Medicine Huddinge, Karolinska Institutet, SE-14183 Huddinge, Sweden; 5 Vecura, Department of Laboratory Medicine, Karolinska Institutet, SE-14186 Stockholm, Sweden; 6 Vecura, Karolinska Cell Therapy Center, Karolinska University Hospital, SE-14186 Stockholm, Sweden; 7 Department of Clinical Science, Intervention and Technology, Karolinska Institutet, SE-14183 Huddinge, Sweden; 8 XNK Therapeutics AB, Hälsovägen 7, Novum, SE-14157 Huddinge, Sweden; 9 Center for Hematology and Regenerative Medicine, Division of Clinical Immunology and Transfusion Medicine, Department of Laboratory Medicine, Karolinska Institutet, SE-14183 Huddinge, Sweden; 10 Pathology Unit, Department of Laboratory Medicine, Karolinska Institutet, SE-14183 Huddinge, Sweden; 11 Department of Clinical Pathology and Cytology, Karolinska University Hospital, SE-14186 Huddinge, Sweden; 12 Department of Cellular Therapy and Allogeneic Stem Cell Transplantation, Karolinska University Hospital Huddinge, SE-14186 Huddinge, Sweden; 13 Division of Hematology, Department of Medicine Huddinge, Karolinska Institutet, SE-14183 Huddinge, Sweden

**Keywords:** immunotherapy, NK cells, myeloma, consolidation, adoptive cell therapy, hematology, immunotyping, granzyme B

## Abstract

Few approaches have been made toward exploring autologous NK cells in settings of cancer immunotherapy. Here, we demonstrate the feasibility of infusing multiple doses of *ex vivo* activated and expanded autologous NK cells in patients with multiple myeloma (MM) post-autologous stem cell transplantation. Infused NK cells were detected in circulation up to 4 weeks after the last infusion. Elevations in plasma granzyme B levels were observed following each consecutive NK cell infusion. Moreover, increased granzyme B levels were detected in bone marrow 4 weeks after the last infusion. All measurable patients had objective, detectable responses after NK cell infusions in terms of reduction in M-component and/or minimal residual disease. The present study demonstrates that autologous NK cell-based immunotherapy is feasible in a setting of MM consolidation therapy. It opens up the possibility for usage of autologous NK cells in clinical settings where patients are not readily eligible for allogeneic NK cell-based immunotherapies.

## Highlights

• Infusing activated and expanded autologous NK cells in patients with MM is possible
• Infused NK cells are detected in circulation for up to 4 weeks
• Elevated granzyme B levels are observed following each consecutive NK cell infusion
• Objective, detectable responses after NK cell infusions are seen in patients

## Graphical abstract

**Figure:**
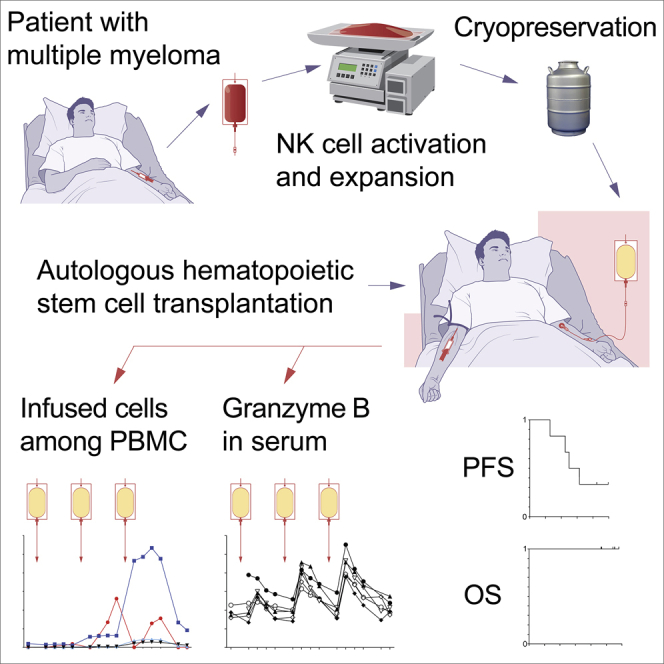

## Teaser

Activated and expanded autologous NK cells were repeatedly infused in patients with multiple myeloma following autologous HSCT. The study by Nahi et al. shows safety and demonstrates efficacy parameters. It opens up the possibility to use autologous NK cells in clinical settings, either alone or as parts of combination therapies.

## Introduction

In recent years, a plethora of allogeneic natural killer (NK) cell-based immunotherapy trials have been reported with promising results.^1^, ^2^, ^3^, ^4^, ^5^, ^6^ The rationale for using allogeneic NK cells was initially a consequence of insights into the molecular specificity of NK cells, in particular their capacity to mediate missing self-reactivity.^7^^,^^8^ Applicability was further supported by findings on the role of NK cells in haploidentical hematopoietic stem cell transplantation (HSCT).^9^^,^^10^ The “off-the-shelf” applicability of allogeneic NK cells has recently further boosted an interest in their clinical use.^6^^,^^11^, ^12^, ^13^, ^14^, ^15^ As a result, allogeneic NK cell-based immunotherapy strategies currently dominate the field of adoptive NK cell-based cancer immunotherapy (see, e.g., Tschan-Plessl et al. 2021).^16^

In contrast, few studies currently encompass the use of autologous NK cells. As such, the concept of using autologous NK cells, however, is not new. Adoptive transfer of autologous NK cells to patients with cancer originates from studies in the mid-1980s.^17^ Yet, these and a few other attempts with autologous NK cells were met with mixed results at best,^18^ at least in part due to the clinical setting in which they were used. For example, clinical treatment of patients with refractory solid tumors, such as progressive stage IV melanoma or renal cancer, was unsuccessful.^19^ Despite these results, we have revisited the potential of using activated and expanded autologous NK cells in a refined clinical consolidation setting, where allogeneic NK cells would not readily be applicable and where autologous NK cells potentially could be effective.

We here present results from an investigator-driven first-in-human clinical trial utilizing *ex vivo* activated and expanded NK cells following autologous HSCT in patients with multiple myeloma (MM). The study design included administration of 3 escalating doses of activated and expanded NK cells in weekly intervals for each patient. Clinical results, including safety analysis and surrogate exploratory parameters indicative of effector functions, are presented. Conceptually, these early results open up the possibility for further assessment of autologous NK cell-based immunotherapies in specific clinical settings; e.g., low tumor burden, minimal residual disease (MRD), or consolidation treatment in malignant diseases.

## Results

### Clinical development of autologous NK cell-based immunotherapy for MM patients

Activated and expanded NK cells display an increased cytotoxic activity against autologous primary MM cells *ex vivo*.^20^ This, and results from earlier studies,^21^, ^22^, ^23^ led to the hypothesis that autologous *ex vivo* activated and expanded NK cells could be evaluated in clinical settings. Hence, we designed a clinical study exploring the feasibility and safety of adoptively transferred activated and expanded autologous NK cells to patients with MM. For this study, a feeder-free, GMP-compliant, automated and closed system for activation and expansion of NK cells from patients with malignant diseases was adopted.^24^ Based on this protocol, a first-in-human investigator-initiated clinical study was initiated in patients having undergone autologous HSCT for MM. The clinical study was designed as an open, single-arm study. The primary objective was to assess safety and tolerability of the NK cell-based product. Secondary objectives were to assess disease response, measured by monoclonal immunoglobulin levels, serum-free light chain, and MRD according to the international myeloma working group (IMWG) uniform response criteria.^25^ The clinical characteristics of patients treated are summarized in Table 1. Additional patient information is listed in Table S1. The setup of the study is outlined in Figure S1 and described in more detail in Methods S1. In the study, 6 study subjects received 3 escalating doses each of activated and expanded NK cells in weekly intervals.

**Table 1:** Patients infused with the investigational NK cell-based product

Study subject	Gender	Performance status^a^	Stage of disease^b^	Type of M component	Risk group according to FISH^c^	Bone lesion	BM plasma cells at diagnosis, %
P103	F	1	III/II	IgGκ	SR	Yes	15
P105	F	1	II/II	IgGκ	SR	Yes	60
P106	M	0	II/II	IgGκ	HR	Yes	47
P107	M	1	-d	κ^e^	HR	Yes	39
P110	M	1	II/II	IgAκ	SR	Yes	97
P111	M	1	I/I	IgDκ	SR	Yes	18

a Performance status grouped according to Eastern Cooperative Oncology Group (ECOG)^26^.

b International staging system (ISS)^27^/revised international staging system (R-ISS)^28^.

c SR/HR denoted standard/high risk as classified by the International Myeloma Working Group (IMWG),^29^ fluorescence *in situ* hybridization (FISH).

d Staging not possible due to lack of B_2_M analysis at diagnosis.

e Light-chain kappa.

### Flow cytometry-based tracking of infused autologous NK cell product in patients

**Figure 1:**
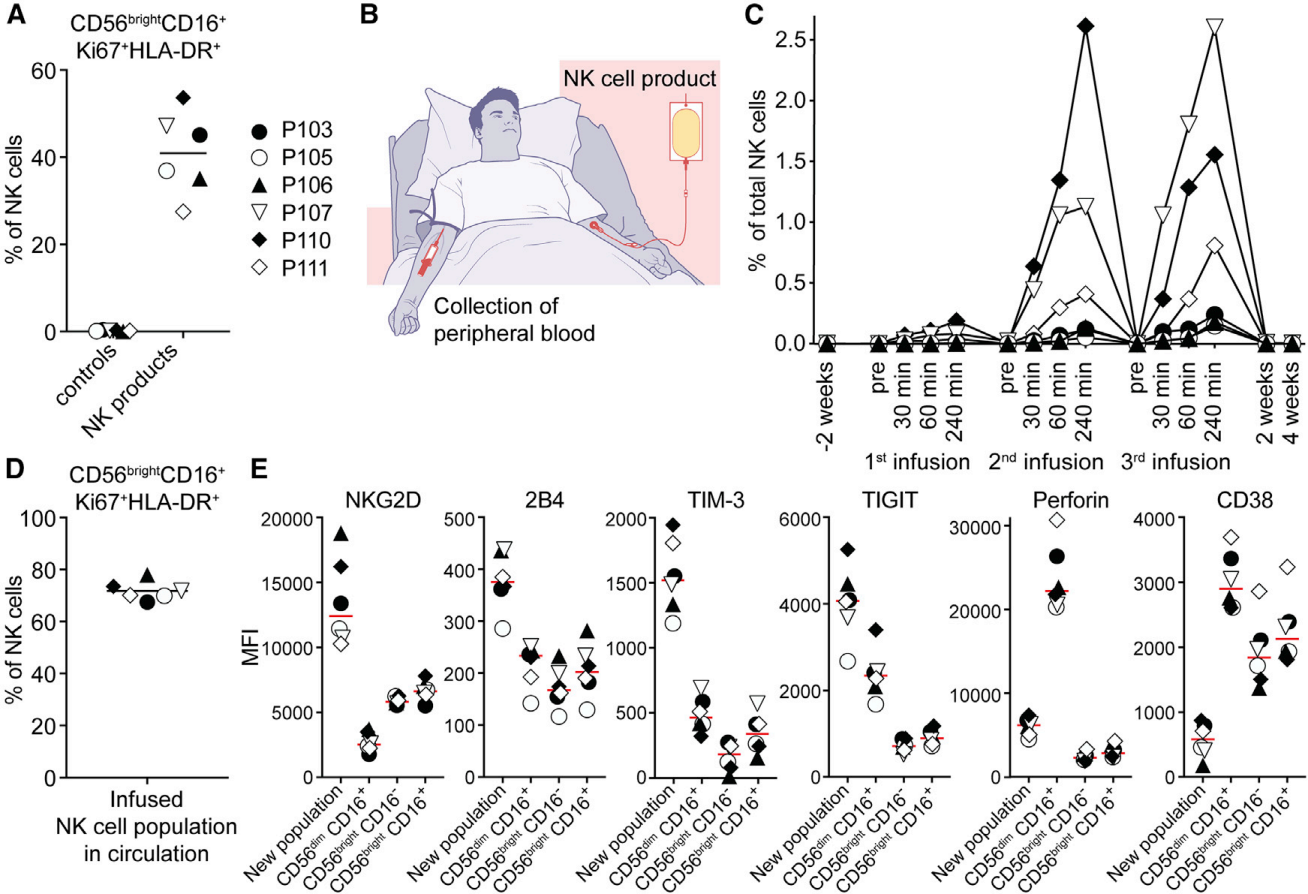
Flow-cytometry-based tracking of infused autologous NK cell product in patients (A) Percentage of *ex vivo* activated and expanded NK cells with the CD56^bright^CD16^+^Ki67^+^HLA-DR^+^ phenotype in the NK cell products. Controls represent study subject peripheral blood NK cells before the first infusion. Lines represent the mean values. Symbols represent patients in all panels displayed (n = 6). (B) Strategy employed to detect *ex vivo* activated and expanded autologous NK cells among peripheral blood NK cells directly following the infusion. (C) Relative size of a defined subset of the infused NK cell population as detected in the circulation of study subjects after infusion of the NK cell product. Infused NK cells were identified by their distinct phenotype by using t-SNE analysis. (D) Percentage of NK cells with the CD56^bright^CD16^+^Ki67^+^HLA-DR^+^ phenotype within the infused cell populations followed in (C). Data shown are pooled from all time points. Line represents the mean value. (E) Median fluorescence intensities of selected markers on the infused NK cell populations followed in (C) compared with other NK cell subpopulations. Data shown are pooled over all time points. Lines represent the median values.

**Figure 2:**
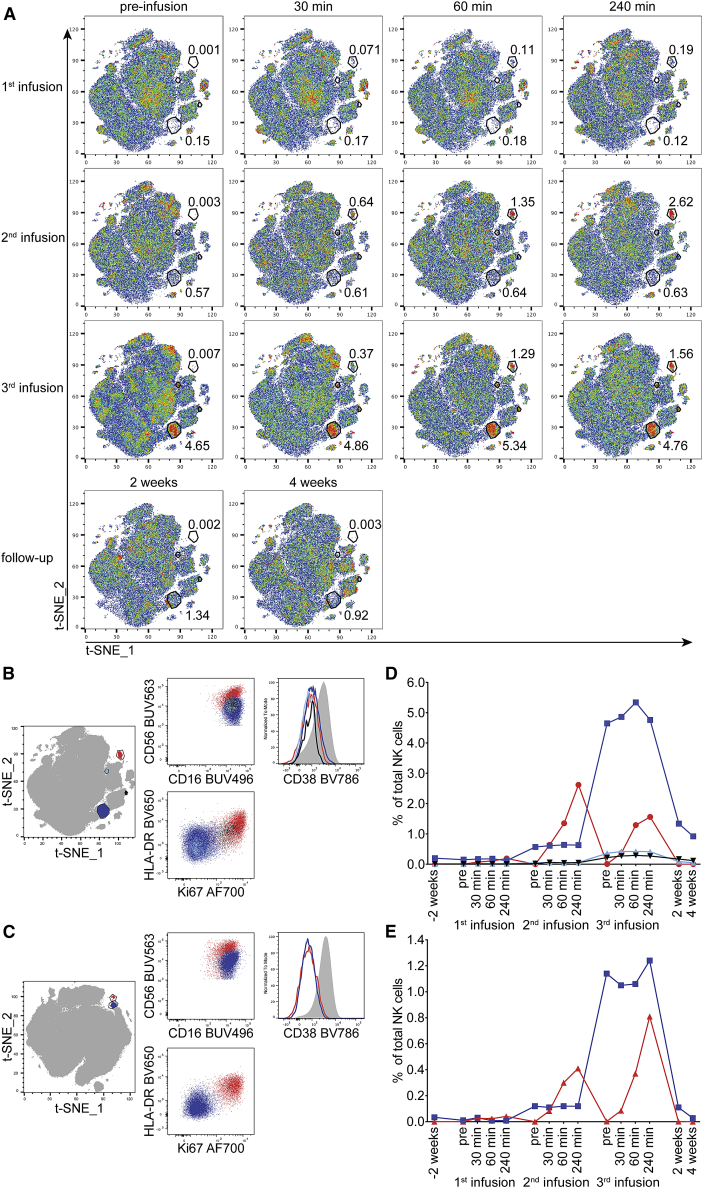
Temporal appearance and phenotype of infused populations within study subject peripheral blood NK cells (A) The temporal appearance of infused populations within the study subject peripheral blood NK cells. Representative t-SNE analysis based on 19 markers of 1 study subject (P110) is shown. The numbers next to the gates represent the percentage of that population within total NK cells at the respective time point. Two populations with different kinetics of appearance are marked (the population on the top right is included in Figure 1C). (B–E) (B and C) Comparison of the phenotypes. (D and E) The relative sizes of the infused NK cell populations detected in the circulation after infusion of the NK cell product for study subject P110 (B and D) and study subject P111 (C and E). The color coding in the t-SNE plots on the left represents the populations in the graphs to the right.

Upon *ex vivo* activation and expansion, we observed that the NK cells gained a unique activated phenotype that includes populations of CD56^bright^CD16^+^Ki67^+^HLA-DR^+^ NK cells (Figure 1A; Figure S2). We hypothesized that this specific phenotype might allow us to detect the infused NK cells among peripheral blood NK cells directly following the infusion to the study subjects. To address this, peripheral blood was collected from the non-NK cell infusion arm (median cubital vein) directly before and at several time points after each consecutive NK cell-based product-infusion (illustrated in Figure 1B). Cells taken from the non-NK cell infusion arm were analyzed by multiparameter flow cytometry. Clustering analyses of data from these samples using t-distributed stochastic neighbor embedding (t-SNE) revealed the gradual appearance of the new NK cell population in a dose-dependent fashion over 4 h following infusion of the NK cell product (Figures 1C and 1D; Figure S3). Furthermore, comparable to the NK cell infusion product, these cells expressed high levels of NKG2D, 2B4, TIM-3, and TIGIT, as well as surprisingly low levels of CD38 (Figure 1E; Figure S4). In 2 of the study subjects, we also observed a gradual appearance of a CD56^dim^CD16^+^Ki67^dim^HLA-DR^dim^ NK cell population (Figures 2A, 2B, 2C, and S5), persisting for at least 4 weeks (Figures 2D and 2E). These observations suggest a phenotypic drift following infusion, potentially due to tumor exposure and reduced non-specific inflammatory stimuli such as IL-2.

### Plasma proteomic assessment of granzymes and pro-inflammatory cytokines in conjunction with NK cell product infusion

**Figure 3:**
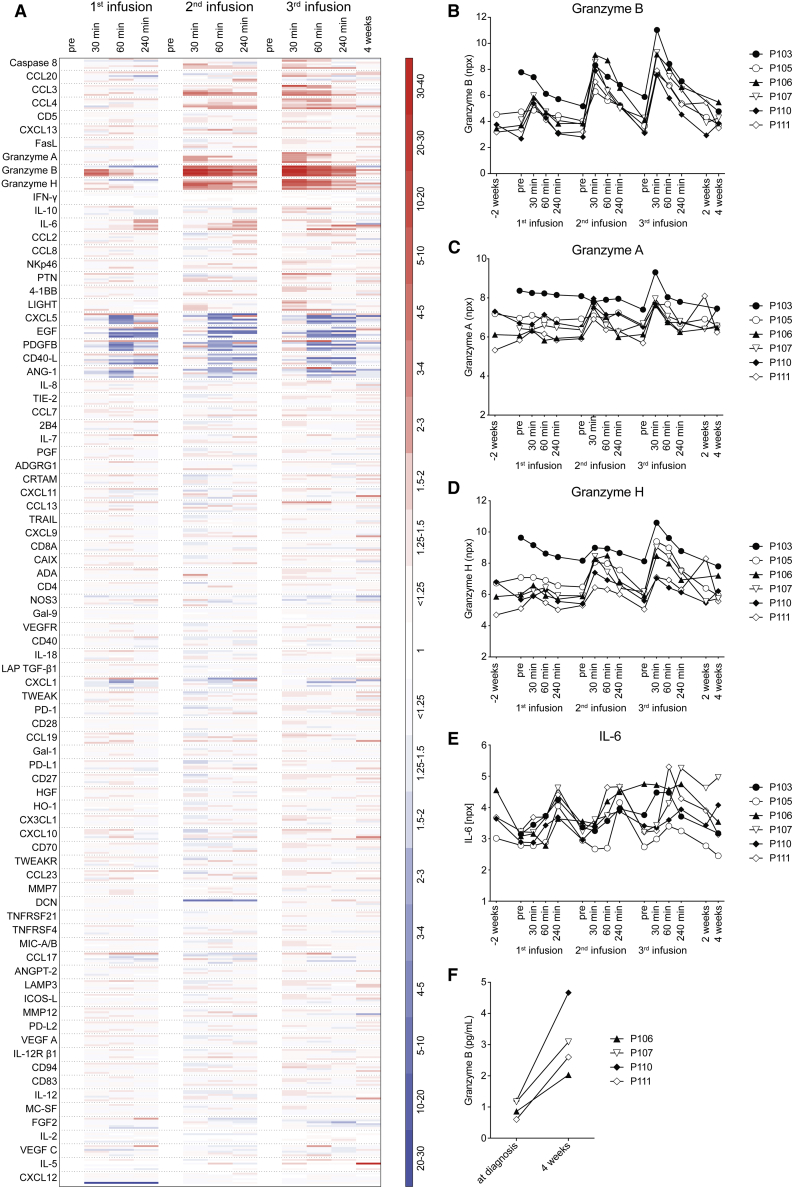
Assessment of the plasma proteome in conjunction with each NK cell product infusion (A) A total of 92 plasma proteins were assessed by proximity extension assay. As described in STAR Methods, 8 were excluded from analysis. The heat map shows the log2-based npx values from each of the 6 study subjects translated into fold change related to the value of the pre-infusion sample of each infusion. Red indicates fold increase; blue indicates fold decrease. (B–E) Assessment of peripheral blood plasma granzyme B (B), granzyme A (C), granzyme H (D), and IL-6 (E) in relation to infusion of *ex vivo* activated and expanded NK cells. The relative concentration was measured by proximity extension assay and is presented in arbitrary log2-based units npx for all study subjects (n = 6). (F) Assessment of bone marrow plasma granzyme B at diagnosis and after infusion of *ex vivo* activated and expanded NK cells by ELISA. Data shown from 4 study subjects; p = 0.021, paired t test.

In parallel with the detailed analysis of NK cell subpopulations in peripheral blood, we assessed the plasma proteome in conjunction with each consecutive NK cell product infusion (Figure 3A). With the exception of the first infusion to study subject P103, a significant and dose-dependent increase, peaking 30 min after each NK cell-product infusion, was observed for granzyme B (Figure 3B). Similar, but less marked, patterns were observed for granzyme A and granzyme H (Figures 3C and 3D) and the pro-inflammatory molecules CCL3 (MIP1-α) and CCL4 (MIP1-β) (Figure 3A). IL-6 levels peaked at 60–240 min after each infusion (Figure 3D). Absolute values for IL-6 as measured by ELISA, however, did not exceed 17 pg/mL. For other plasma proteins, no or insignificant responses were observed (Figure 3A). Finally, an increase in granzyme B was also observed in plasma from bone marrow aspirates taken 4 weeks after the last infusion (Figure 3F; data shown from 4 study subjects from which bone marrow aspirate material was available for analysis).

### Clinical assessment of MM patients following autologous NK cell-based immunotherapy

**Figure 4:**
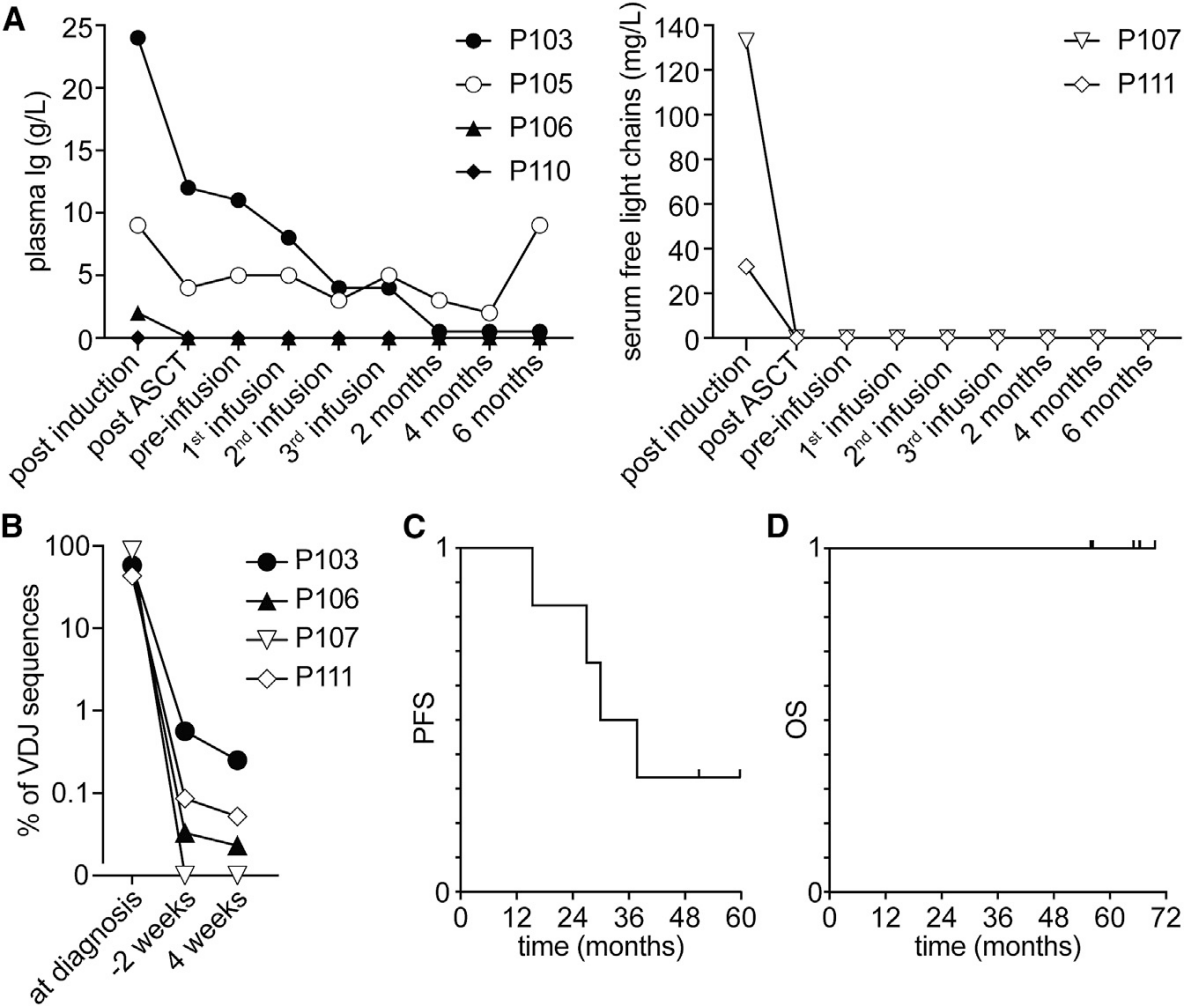
Outcome of autologous NK cell-based immunotherapy for consolidation treatment of patients with multiple myeloma (MM) (A) Dynamics of plasma M-component (left, n = 4) and serum-free light chains (right, n = 2) in study subjects in the course of the clinical study. ASCT, autologous stem cell transplant. (B) IgH variability, diversity, and joining**(**VDJ) rearrangement analysis of BM samples taken at diagnosis and respective MRD values 2 weeks before the first and 4 weeks after the last infusion of the NK cell product. Percentages of the clonal IgH VDJ sequence (as identified in MM diagnosis samples) out of total IgH VDJ sequences are displayed. Data from 4 of 6 study subjects are shown. (C and D) (C) PFS and (D) OS of all study subjects (n = 6) during the course of the study calculated from the time of inclusion.

The clinical effects of the NK cell product infusions were assessed during a 6-month follow-up period. At the time of the first NK cell product infusion, 2 of 6 patients had a detectable M-component. Both of these showed a decrease in M-component following the first NK cell infusion, and additionally, after the completion of the infusion period. In patients with no detectable M-component, whether serum Ig (Figure 4A, left) or free light chains (Figure 4A, right), the M-component remained undetectable throughout the entire infusion period and during the full 6-month follow-up period. In 3 of 4 assessed study subjects, a deepening of MRD was observed 4 weeks after the last infusion (Figure 4B; Table S2). The fourth study subject was MRD negative at the time of infusion and remained MRD negative 4 weeks after the last infusion. Median progression-free survival (PFS) was 34 months (Figure 4C). All six study subjects are still alive, during a minimum 60-month follow-up period (Figure 4D). Additional data, including response status after autologous HSCT, time from autologous HSCT to infusion, NK cell product doses infused, and follow-up response status, are displayed in Table S3.

### Safety assessment of MM patients following autologous NK cell-based immunotherapy

**Figure 5:**
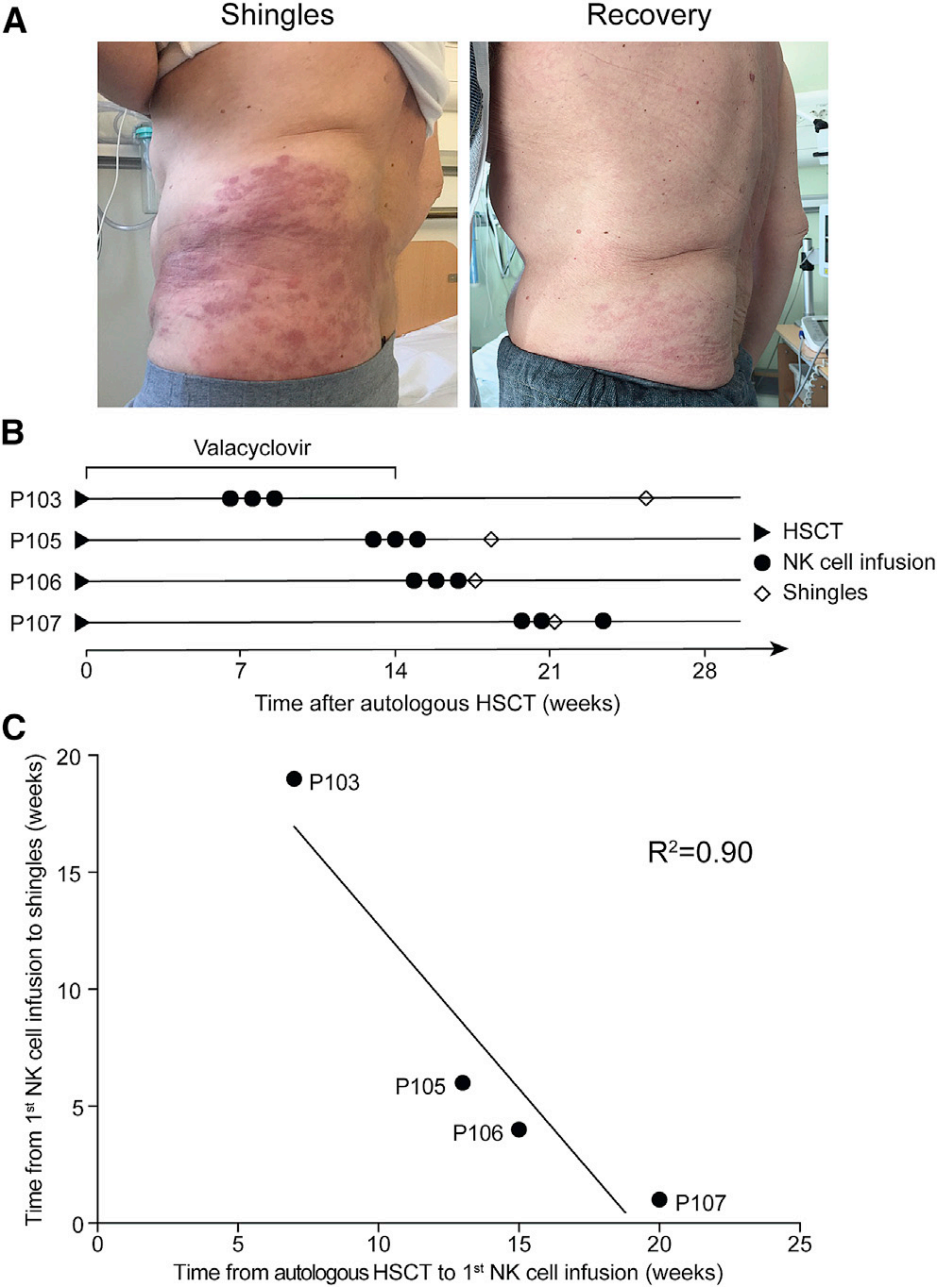
Development of shingles in the first 4 patients following NK cell product infusion (A) Shingles in 1 study subject following NK cell product infusion (left) and recovery after antiviral treatment (right). (B) Development of shingles in relation to NK cell product infusion. Diamonds mark the first appearance of shingles. Period of post-HSCT valacyclovir treatment is indicated (n = 4). (C) Correlation analysis between the time from HSCT to the NK cell product infusion and the time from NK cell product infusion to development of shingles. Regression calculated by Pearson correlation.

The safety of the NK cell product infusions was assessed immediately and for a 6-month follow-up period. No severe adverse events (SAE) were observed. A complete list of treatment-emergent adverse events (TEAE) is displayed in Table S4. Overall, the clinical study demonstrated safety and tolerability. However, unexpectedly, the first 4 study subjects developed herpes zoster (HZ) following the infusion of the NK cell product (Figure 5A). In all cases, HZ development occurred after the antiviral prophylaxis with valacyclovir following the autologous HSCT had ended. In 3 of the 4 study subjects, it manifested after the third NK cell product infusion, and in 1 of the study subjects, it had already manifested at the time of the second infusion (Figures 5B and 5C). Clinically, shingles manifestation was managed with standard antiviral treatment. Upon consultancy with the Swedish Medical Products Agency, the last 2 study subjects were prophylactically treated with valacyclovir (500 mg) twice daily for 6 months from the first NK cell product infusion. No signs of shingles were observed in these 2 study subjects.

## Discussion

The present study has explored the potential use of autologous *ex vivo* activated and expanded NK cells in patients with MM. A particular emphasis was on clinical settings where allogeneic NK cells would not readily be applicable and where there would be an unmet need to explore alternative immunotherapy approaches to treatment. On the basis of *ex vivo* and *in vivo* results from experimental MM model systems, we proceeded with a clinical study in MM patients following autologous HSCT. Although autologous HSCT, following conditioning, serves to reduce the tumor burden, all MM patients eventually relapse.^30^^,^^31^ Hence, in this setting, there is ample reason for the exploration of novel complementary approaches. We here report that escalating doses of *ex vivo* activated and expanded autologous NK cells are well tolerated in MM patients without any SAEs. The 4 of the total 6 patients that had measurable disease following HSCT showed indications of objective, measurable responses to NK cell infusions in terms of reduction in M-component (serum Ig) and/or MRD. Exploratory analyses revealed increased numbers of infused NK cells in patients’ peripheral blood and marked elevations in plasma granzyme B correlating to each individual consecutive infusion. Furthermore, increased levels of granzyme B were also detected in bone marrow aspirates 4 weeks after the last infusion.

No SAE, nor any signs of cytokine release syndrome (CRS) were observed. IL-6 presented with only moderate transient fluctuations following the infusion of the NK cell product. With respect to the manifestation of shingles in the first 4 patients, it is unlikely that the autologous HSCT *per se* is responsible for the HZ reactivation observed in 4 consecutive patients, especially given the time from autologous HSCT to shingles development.^32^ The mechanism for the HZ activation is unknown, but it is fathomable that it could relate to the infusion of the autologous NK cell product. Noteworthy, the present study protocol was originally designed without antiviral prophylaxis upon NK cell product infusion. We speculate that activated NK cells could have attacked reservoir cells of the varicella zoster virus that, in turn, could have caused a viral reactivation. Corroborating this hypothesis, it has been previously reported that activated NK cells can recognize dorsal root ganglia cells in experimental model systems.^33^, ^34^, ^35^ At the present stage, we conclude that autologous NK cell-based immunotherapy, even when performed without a lymphodepletion regimen, should be combined with adequate prophylactic antiviral treatment.

In this study, we attempted to follow the infused activated NK cell population, something that turned out to be feasible in the current clinical context, given the specific features of the NK cell product. The rationale for this approach was the fact that the fate of these cells was unclear. Additionally, the phenotypic dynamics of the infused cell population was equally unclear. The infused cells were traceable for a period of 4 weeks and retained a CD16^+^CD38^dim^ phenotype, a feature that may enable future combinatorial therapies.

While there is a strong rationale for the use of allogeneic NK cell-based immunotherapies, there might be time to revisit the partially omitted field of autologous NK cell-based therapies. Most importantly, the latter may fill a space not easily covered by allogeneic NK cell-based therapies. We base this on the following arguments. Efficient and cost-effective *ex vivo* activation and expansion protocols for human NK cells have now been developed, including the feeder-free protocols as used here. Current protocols allow efficient activation and expansion of NK cells also from patients with malignant diseases.^20^^,^^36^ As verified for cells undergoing activation and expansion using the protocol employed here, long-term storage of patients’ own activated and expanded NK cells is feasible with indications of more than 10 years of stability. The latter may have implications for the development of future therapies. For example, it opens up the possibility for multiple dosing over long periods of time. Importantly, autologous NK cells also allow for infusion to the patient without immunosuppressive conditioning. Possible limitations with respect to autologous NK cell-based products are the obvious lack of off-the-shelf availability, recurring manufacturing costs, and rapid accessibility of the potential therapeutic product.

In adoptive NK cell-based immunotherapy, antitumor activity by NK cells depends on their degree of education/licensing, a process predominantly determined by their interactions with self major histocompatibility complex (MHC) class I molecules on other cells.^37^, ^38^, ^39^ Given this fact, the administration of NK cells to an autologous host sets the stage for an optimal licensing process via NK cell inhibitory receptors interacting with self MHC class I. That is, a process allowing for optimal recognition of “missing self;” i.e., specific tumor target cells lacking some or all self MHC class I molecules.^40^ In this context, there is abundant evidence demonstrating partial or complete loss of histocompatibility leukocyte antigen (HLA) class I expression in a wide spectrum of human tumor types.^41^^,^^42^ Such loss may result from the immune selection by CD8^+^ T cells and has also more recently been linked to acquired resistance to checkpoint inhibition therapy.^43^ Deduced from the latter studies, and the early predictions of the missing self-concept, autologous NK cell-based adoptive immunotherapy may offer a possible treatment strategy in MHC class I-deficient and, hence, T cell checkpoint inhibitor-therapy refractory patients.^44^ Beyond this, current checkpoint inhibitors, e.g., PD1 or PD1L inhibitors may also exert effects on endogenous NK cells and, hence, possibly also enhance the efficacy of adoptively transferred NK cells.^45^^,^^46^ In the latter context, new generations of more NK cell-specific checkpoint inhibitors are also under development.^47^

As noted above, autologous NK cells readily allow multiple dosing, a strategy that may be particularly challenging in many settings of allogeneic therapies. As deduced from the example of the present clinical study, we reason that autologous NK cell-based therapies may be particularly suited for patients with low tumor burden, e.g., in hematological remission but MRD positive. The present reasoning may also apply to other indications where tumor reduction is achieved by irradiation, surgical resection, and/or chemotherapy. This builds on earlier studies in experimental model systems, demonstrating the capacity of NK cells to preferentially target small tumor grafts.^48^

In the present context of autologous NK cell-based immunotherapies, it is noteworthy that the rapid development and recent advances in the autologous T cell therapy field; e.g., the isolation and usage of tumor-infiltrating lymphocytes (TILs)^49^ or chimeric antigen receptor (CAR) T cells^50^ have made stunning progress. In this context, despite obvious differences among T cells and NK cells, the overall development of autologous NK cell-based immunotherapies has been significantly lagging behind that of T cells. However, CARs are now making their way into adoptive NK cell-based immunotherapies.^51^, ^52^, ^53^ One feature of NK cells in contrast to T cells is that they, in addition to introduced CARs, may still be able to recognize tumors through their germline-encoded receptors, reducing the potential risk of tumor escape through antigen modulation. Finally, autologous NK cell products could readily be used in combination with other treatment modalities such as monoclonal antibodies,^54^^,^^55^ bi or tri-specific-engagers,^56^^,^^57^ proteasome inhibitors,^58^ or other types of immunomodulatory drugs.

In summary, the results presented here open up the possibility for further clinical studies utilizing autologous NK cells, e.g., in settings where patients are not readily eligible for allogeneic NK cell-based products. The latter could include MRD and/or consolidation treatment in human cancer. Provided use of properly activated and expanded cells, whether or not in combinations with other drugs, the present results suggest that use of autologous NK cells merits further investigation in the context of future cancer treatment.

### Limitations of the study

Although positive aspects of the study are observed, the present study, focusing on safety and feasibility assessment, is not sufficient to provide a firm conclusion of clinical efficacy of the present treatment. For the latter, a larger study including a control group is needed. Even though it is alluring to compare progression-free and overall survival rates to the same time period cohorts, we believe it might be misleading, considering the small size of the trial. It cannot be excluded that newer maintenance therapies introduced in this patient group may affect the present therapeutic strategy. With respect to the granzyme B data, it cannot be concluded that this is due to the interaction between infused immune effector cells and tumor cells. Likewise, the physiopathology behind shingles manifestation is speculative, based on earlier studies.

Finally, NK cell expansions in closed bioreactor systems are technically challenging and require extensive process optimization. As mentioned above, of 10 expansions, 6 were able to fulfill the release criteria, whereas 1 was directly attributable to a bioreactor-specific consumable error and another was attributable to bioreactor technical error. It is not uncommon to have a higher rate of batch manufacturing failures in early clinical trials. However, the process has currently been optimized, resulting in decreased failure rates.

## STAR★Methods

### Key resources table

**Table:** 

REAGENT or RESOURCE	SOURCE	IDENTIFIER
**Antibodies**		

Mouse monoclonal anti-CD56 BUV563 (clone NCAM16.2)	BD BioSciences	Cat# 565704, Lot# 8250989; RRID: AB_2744431
Mouse monoclonal anti-CD16 BUV496 (clone 3G8)	BD BioSciences	Cat# 564653, lot# 8291900; RRID: AB_2744294
Mouse monoclonal anti-NKp44 AlexaFluor647 (clone p44-8)	BD BioSciences	Cat# 558564, lot# 5082973; RRID: AB_647153
Mouse monoclonal anti-CD25 PE-CF594 (clone M-A251)	BD BioSciences	Cat# 562403, lot# 9099748; RRID: AB_11151919
Mouse monoclonal anti-PD1 BB700 (clone EH12.1)	BD BioSciences	Cat# 566460, Lot# 9182916, 8346689; RRID: AB_2744348
Mouse monoclonal anti-HLA-DR BV650 (clone G46-6)	BD BioSciences	Cat# 564231, lot# 9017957; RRID: AB_2738685
Mouse monoclonal anti-TIM-3 BV711 (clone 7D3)	BD BioSciences	Cat# 565566, lot# 9049619, 9116947; RRID: AB_2744370
Mouse monoclonal anti-CD38 BV738 (clone HIT2)	BD BioSciences	Cat# 563964, lot# 7338505, 9151925; RRID: AB_2738515
Mouse monoclonal anti-DNAM-1 BUV395 (clone DX11)	BD BioSciences	Cat# 742498, lot# 9136597, 9276805, 9276799, 9276800; RRID: AB_2740831
Mouse monoclonal anti-CXCR4 BUV737 (clone 12G5)	BD BioSciences	Cat# 741862, lot# 9276807, 9276809, 9276810; RRID: AB_2871192
Mouse monoclonal anti-Ki67 AlexaFluor700 (clone B56)	BD BioSciences	Cat# 561277, lot# 7349946; RRID: AB_10611571
Mouse monoclonal anti-perforin 1 BB755-P (clone δG9)	BD BioSciences	Custom conjugate, Cat# 624391, lot# 9101891
Mouse monoclonal anti-granzyme B BB790-P (clone GB11)	BD BioSciences	Custom conjugate, Cat# 624296, lot# 9130980
Mouse monoclonal anti-CD19 V500 (clone HIB19)	BD BioSciences	Cat# 561121, lot# 7066974; RRID: AB_10562391
Mouse monoclonal anti-CD14 V500 (clone MΦP9)	BD BioSciences	Cat# 562693, lot# 8215846; RRID: AB_2737727
Mouse monoclonal anti-CD3 PE-Cy5 (clone HIT3a)	BioLegend	Cat# 300310, lot# B181404, B291468; RRID: AB_314046
Mouse monoclonal anti-NKG2D BV421 (clone 1D11)	BioLegend	Cat# 320821, lot# B274633; RRID: AB_2566510
Mouse monoclonal anti-2B4 APC-Cy7 (clone C1.7)	BioLegend	Cat# 329518, lot# B329518, B219470, B276619; RRID: AB_2572015
Mouse monoclonal anti-CD319 PE (clone 162.1)	BioLegend	Cat# 331806, lot# B268818; RRID: AB_2239190
Mouse monoclonal anti-LAG-3 PE-Cy7 (11C3C65)	BioLegend	Cat# 369310, lot# B289010; RRID: AB_2629753
Mouse monoclonal anti-TIGIT BV605 (clone A15153G)	BioLegend	Cat# 372711, lot# B240084, B281575; RRID: AB_2632926
Mouse monoclonal anti-NKG2A VioBright FITC (clone REA110)	Miltenyi Biotec	Cat# 130-105-646, lot# 5160809205; RRID: AB_2655382
Orthoclone OKT3 (MACS GMP CD3 pure)	Miltenyi Biotech	Cat# 170-076-124

**Biological samples**		

Human AB serum	Lonza	Cat# 14-490E; current Cat# 4W-320

**Chemicals, peptides, and recombinant proteins**		

CellGro SCGM serum-free cell culture media	CellGenix	Cat# 0020902-0500
IL-2 (Proleukin)	Chiron, currently Clinigen	https://www.medicines.org.uk/emc/product/291/smpc
Pluronic F68	Life Technologies	Cat# 24040032

**Critical commercial assays**		

eBioscience Foxp3/Transcription Factor Staining Buffer Set	Thermo Fisher Scientific	Cat# 00-5523-00
LIVE/DEAD Fixable Aqua Dead Cell Stain Kit	Thermo Fisher Scientific	Cat# L34957
LymphoTrack Dx IGH FR1/FR2/FR3 Assay Kit A -MiSeq	Invivoscribe	Cat# 9-121-0129
QIAamp DNA micro kit	Qiagen	Cat# 56304
QIAamp DNA Blood Midi Kit	Qiagen	Cat# 51183
Agencourt AMPure XP beads	Beckman Coulter	Cat# A63880
Qubit dsDNA HS Assay Kit	Invitrogen	Cat# Q32854
Agilent High Sensitivity DNA ScreenTape Assay	Agilent	Cat# 5067-5584
Olink Immuno-Oncology panel v.3101	Olink Bioscience	Cat# 95310
PeliKine Compact human GRANZYME B ELISA Kit	Sanquin CLB	Ref# M1936, no longer available

**Software and algorithms**		

FlowJo v.10	Treestar inc.	RRID: SCR_008520
GraphPad Prism v.8	GraphPad Software	RRID: SCR_002798
LymphoTrack Dx Software – MiSeq Version 2.4.3	Invivoscribe	Cat# 95000009
LymphoTrack MRD Software v1.2.0	Invivoscribe	Cat# 75000008

**Other**		

WAVE Bioreactor™ System 2/10	GE Healthcare	
Cobas 8000 system	Roche Diagnostics	https://diagnostics.roche.com/global/en/products/systems/cobas_-8000-modular-analyzer-series.html#productInfo
Beckman Coulter IMMAGE 800 Protein Chemistry Analyzer	Beckman Coulter	RRID: SCR_019642
BN ProSpec	Siemens Healthcare GmbH	https://www.siemens-healthineers.com/se/plasma-protein/systems/bn-prospec-system
Atellica NEPH 630 system	Siemens Healthcare GmbH	https://www.siemens-healthineers.com/se/plasma-protein/systems/atellica-neph-630-system
Sebia Hydrasys LC system	Sébia	https://www.sebia.com/instruments/hydrasys-2-scan-focusing/

### Resource availability

#### Lead contact

Further information and requests for resources and reagents should be directed to and will be fulfilled by the lead contact, Evren Alici (evren.alici@ki.se).

#### Materials availability

This study did not generate new unique reagents.

### Experimental model and subject details

#### Clinical study protocol for autologous NK cell-based immunotherapy of MM

The synopsis of the study protocol is presented in Methods S1. Informed consent to inclusion in the clinical study was obtained at diagnosis. Eleven patients referred to the Hematological Center, Karolinska University Hospital, were enrolled in the study. One patient withdrew consent for participation prior to blood donation for production of the NK cell product. From ten MM patients peripheral blood was collected and NK cells were activated and expanded *ex vivo* as described above. NK cell products from six patients met the release criteria. These patients were infused with the investigational NK cell-based product and were followed in the study (P103, P105, P106, P107, P110, P111). The patient characteristics at inclusion in the study including Ig heavy chain, serum free light chain, Eastern Cooperative Oncology Group (ECOG) scale of performance status,^26^ and international staging system (ISS) classification for MM^27^^,^^28^ are summarized in Table 1. Additional clinical chemistry data on study subjects is provided in Table S1. Briefly, following upfront induction with three to four cycles of cyclophosphamide, bortezomib, and dexamethasone (CyBorD), patients underwent autologous HSCT. Conditioning for HSCT was melphalan 200 mg/m^2^ single day. Following autologous HSCT, standard antiviral prophylaxis (valacyclovir) was administered orally twice daily during the first 100 days for the first four study subjects and for six months after the first NK cell product infusion for the fifth and sixth study subject. The study subjects received NK cell infusions when they were in either subclinical relapse after either complete remission (CR), stable partial remission (PR), or PR with asymptomatic progression. The study subjects that had not relapsed or progressed still received NK cell infusions at six months. Study subjects received three escalating doses of 5 × 10^6^ (dose 1), 5 × 10^7^ (dose 2) and up to 1 × 10^8^ (dose 3) NK cell product/kg at weekly intervals (when not noted otherwise). Study subjects were then evaluated for six months after the last infusion. The patients were thereafter continuously followed clinically for up to five years. Of note, the clinical study protocol allowed treatment of twelve patients (Methods S1). However, upon consultation with the Swedish Medical Products Agency during the interim analysis of the clinical study, the cell therapy product with the doses infused was deemed to fulfill the requirements (primary endpoints) outlined in the study protocol. Consequently, the clinical trial was concluded after the interim analysis.

#### Study approval

The clinical study was approved by the Swedish Medical Products Agency (151:2010/63508; amendment: 5.1-2013-77703) and the Stockholm Regional Ethical Review Board (2010/1618-31/4; amendments: 2013/490-32 and 2018/1899-32). Written informed consent was obtained from the study subjects before inclusion in the study. Studies outside the clinical protocol were approved by the Stockholm Regional Ethical Review Board (2008/1166-31).

### Method details

#### Generation of good manufacturing practice (GMP) *ex vivo* activated and expanded NK cells for the clinical study

The NK cell product used was produced under GMP conditions according to a previously described procedure.^24^ Briefly, peripheral blood lymphocytes were separated from 450 mL blood, donated by the patient at diagnosis. The lymphocytes were expanded for 20 days using a closed Wave bioreactor system (WAVE Bioreactor™ System 2/10, GE Healthcare). The cells were grown in a disposable cell bag, containing CellGro SCGM serum-free cell culture media (CellGenix), supplemented with 500 IU/mL IL-2 (Proleukin, Chiron), 10 ng/mL orthoclone OKT3 (Miltenyi Biotech), 5% (v/v) human AB serum (Lonza) and 0.1% (v/v) pluronic F68 (Life Technologies). The expanded cell product had a composition and phenotype similar to what was previously reported.^24^ The final product was frozen in human AB plasma with 5% DMSO and stored at −180 °C until use. The product showed stability of at least ten years in −180 °C (Table S5; data from one validation study shown).

#### Measurement of M-component and serum free light chain

Quantification of plasma immunoglobulins (M-component) was performed using an immunoturbidimetric method on a Cobas 8000 system (Roche Diagnostics) or an Immage platform (Beckman Coulter). Light chains in urine and serum were quantified using a nephelometric method on a BN ProSpec or Atellica NEPH 630 system (Siemens Healthcare GmbH). Additional quantification of M-component in plasma was performed with gel-electrophoresis on a Sebia Hydrasys LC system (Sébia).

#### Measurement of minimal residual disease (MRD)

To identify the immunoglobulin heavy chain (IGH) V(D)J sequence of the dominating MM clone(s) at diagnosis, DNA was prepared from dried bone marrow smears on glass slides (prepared at diagnosis. In brief, smears were pre-wetted with drops of Buffer ATL and transferred into a 1.5 mL microcentrifuge tube. Buffer ATL was added to a total of 180 μL and DNA extracted using QIAamp DNA micro kit (Qiagen, cat# 56304) following the manufacturer’s instructions for dried blood spots. The LymphoTrack Dx *IGH* FR1/FR2/FR3 Assay Kit A -MiSeq (Invivoscribe, cat# 9-121-0129) was used to identify and quantify *IGH* gene rearrangements according to manufacturer’s instructions. In brief, IGH VDJ sequences were amplified using the supplied framework region (FR) 1, −2, and −3 primers using 50–100 ng of DNA per reaction. Amplified DNA was purified using Agencourt AMPure XP beads (Beckman Coulter cat#A63880). Samples were quantified with Qubit dsDNA HS Assay Kit (Invitrogen cat#Q32854) and size determined with Agilent High Sensitivity DNA ScreenTape Assay (Agilent cat#5067–5584). Successfully amplified FR libraries were pooled and paired-end sequenced (300 × 300 or 250 × 250 cycles) using the Illumina MiSeq system. The IGH V(D)J sequence of the dominating MM clone(s) at diagnosis was subsequently identified using LymphoTrack Dx Software – MiSeq Version 2.4.3 (Invivoscribe), according to the manufacturer's instructions. To subsequently perform MRD analysis on pre- and post-infusion samples, DNA was prepared using the QIAamp DNA Blood Midi Kit (Qiagen cat#51183). In brief, vital frozen unfractionated BM cells were pelleted and resuspended in 1 mL of PBS before proceeding following the manufacturer’s instructions. IGH VDJ libraries were prepared as described above (with the FR primers successfully used to determine the IGH VDJ rearrangement in diagnosis samples) using four times 2 μg of DNA per sample. Libraries were paired-end sequenced (250 × 250 cycles), and the IGH V(D)J repertoire determined using the LymphoTrackDx software. MRD detection levels and confidence of MRD negativity was subsequently determined (based on the quantity of DNA utilized for library preparation and sequencing depth) using LymphoTrack MRD Software v1.2.0 (Invivoscribe) according to manufacturer’s instructions.

#### Flow cytometry

Frozen peripheral blood mononuclear cell samples where thawed, counted (Nucleocounter NC-3000, ChemoMetec), and stained with LIVE/DEAD Fixable Aqua Dead Cell Stain (Invitrogen) diluted 1:1000 in PBS for 20 min at 4 °C. Staining for surface markers was performed in PBS with 2% fetal bovine serum and 1 mM EDTA using optimized antibody concentrations (20 min at 4 °C). Antibodies for surface staining were prepared in BD Brilliant Stain Buffer (BD Biosciences). After surface staining, cells were fixed and permeabilized using the Foxp3/transcription factor staining buffer set (eBioscience) and were stained with optimized concentrations of antibodies against Ki67, perforin, and granzyme B (30 min at RT). The samples were analyzed on a BD Symphony A5 (BD Biosciences). CS&T research beads (BD Biosciences) were used to ensure stable performance between experiments. Data analysis was performed using FlowJo v10 (Treestar Inc.). Live NK cells were defined as CD56^+^CD3^−^CD19^−^CD14^−^. For each study subject, the NK cell populations from every time point were concatenated into one data set for clustering analysis. t-SNE was performed on the pooled NK cell data for each study subject using the standard settings (1000 iterations, perplexity 30, Barnes-Hutt algorithm). All markers were included in the analysis except for CD3, CD14, CD19, and the dead cell marker. Clusters emerging after the infusions were identified by overlaying t-SNE plots of the data from before and after infusions.

#### Measurement of cytokines, chemokines, and other proteins

92 protein biomarkers were analysed by proximity extension assay using the Olink Immuno-Oncology panel (v.3101) at Olink Bioscience, Uppsala, Sweden. Briefly, the proteins were detected using antibody pairs labeled with specific oligonucleotides that hybridize when in close proximity. Their DNA barcodes are subsequently quantified by real-time PCR. Protein levels are reported in arbitrary units, normalized protein expression (npx). Eight analyses were excluded because the proteins were detected in less than 25% of the samples.

#### Measurement of granzyme B from bone marrow-derived plasma

Granzyme B levels were determined with commercially available ELISA (PeliKine Compact human GrB-ELISA; Sanquin CLB).

### Quantification and statistical analysis

Statistical analyses were performed using Prism 8 (GraphPad Software). Granzyme B levels in BM plasma were compared using paired t-test. p-values < 0.05 were considered statistically significant. Correlation between the time from HSCT to the NK cell product infusion and the time from NK cell product infusion to the development of shingles was calculated using Pearson correlation.

### Additional resources

The study was prospectively registered in the EudraCT database (2010-022330-83) and retrospectively registered at clinicaltrials.gov (NCT04558853).

## Data Availability

• Study protocol-related data have been submitted to European Union Drug Regulating Authorities Clinical Trials Database (EudraCT). Anonymous data and other information will be made available upon request to the corresponding author following publication of the present article. Data will be made available in a form not deviating from what is accepted by local regulatory authorities with respect to handling of patient data, and in adherence of the policies of the Karolinska University Hospital and Karolinska Institutet. • This study does not generate any custom code. • Any additional information required to reanalyze the data reported in this work paper is available from the Lead Contact upon request.

## Supplemental information

Document S1. Figures S1–S5, Tables S1–S5, and Methods S1

Document S2. Article plus supplemental information
