# Supplementary material for: Autologous NK cells as consolidation therapy following stem cell transplantation in multiple myeloma
Source: Cell Rep Med. 2022 Jan 28;3(2):100508. doi: 10.1016/j.xcrm.2022.100508 (PMC8861830; doi:10.1016/j.xcrm.2022.100508)
Supplement: Document S1. Figures S1–S5, Tables S1–S5, and Methods S1 [file mmc1.pdf]

Cell Reports Medicine, Volume *3*

Supplemental information

Autologous NK cells as consolidation therapy following stem cell transplantation in multiple myeloma

Hareth Nahi, Michael Chrobok, Stephan Meinke, Charlotte Gran, Nicole Marquardt, Gabriel Afram, Tolga Sutlu, Mari Gilljam, Birgitta Stellan, Arnika K. Wagner, Pontus Blomberg, Per-Henrik Holmqvist, Lilian Walther-Jallow, Karin Mellström, Johan Liwing, Charlotte Gustafsson, Robert Månsson, Monika Klimkowska, Gösta Gahrton, Johan Lund, Per Ljungman, Hans-Gustaf Ljunggren, and Evren Alici

Supplemental Material to Nahi *et al*. "Autologous NK Cells as Consolidation Therapy Following Stem Cell Transplantation in Multiple Myeloma"

Supplemental Figures

Figure S1

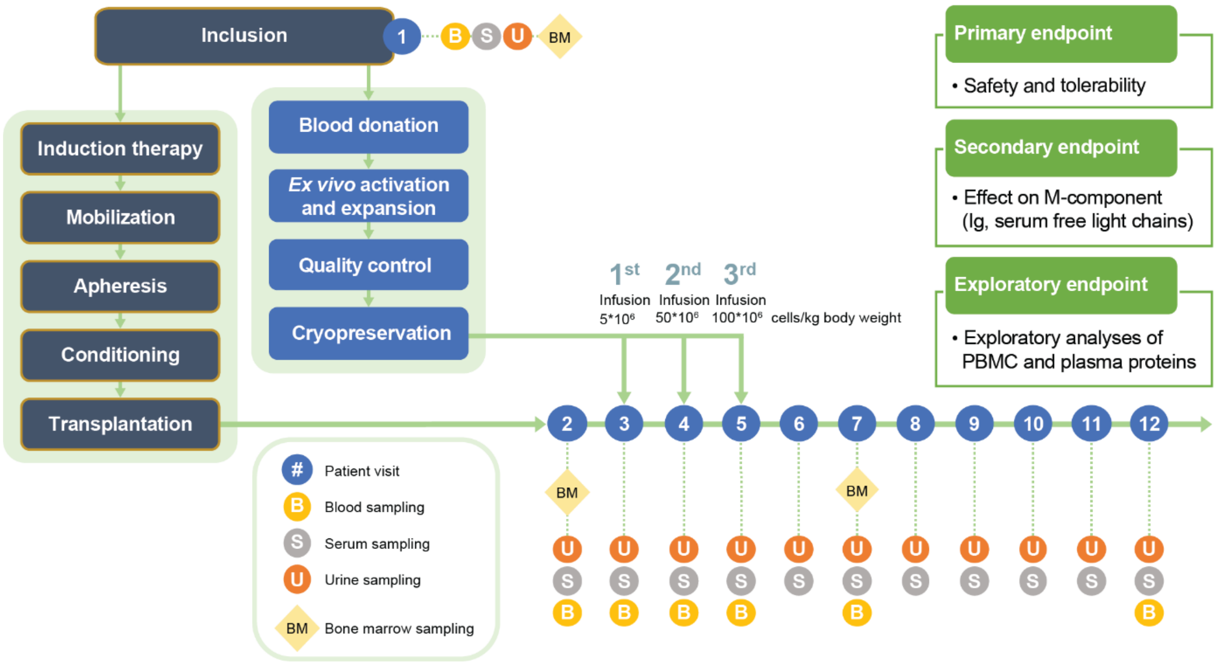

Figure S1. Overview of the clinical study setup. Related to STAR method '*Clinical study protocol for autologous NK cell-based immunotherapy of MM*'. Six study subjects received three escalating doses of 5 x 10^6 (dose 1), 5 x 10^7 (dose 2) and up to 1 x 10^8 (dose 3) NK cell product/kg at weekly intervals. Study subjects were then evaluated for six months following the last infusion. The patients were thereafter continuously followed clinically for up to five years.

Figure S2

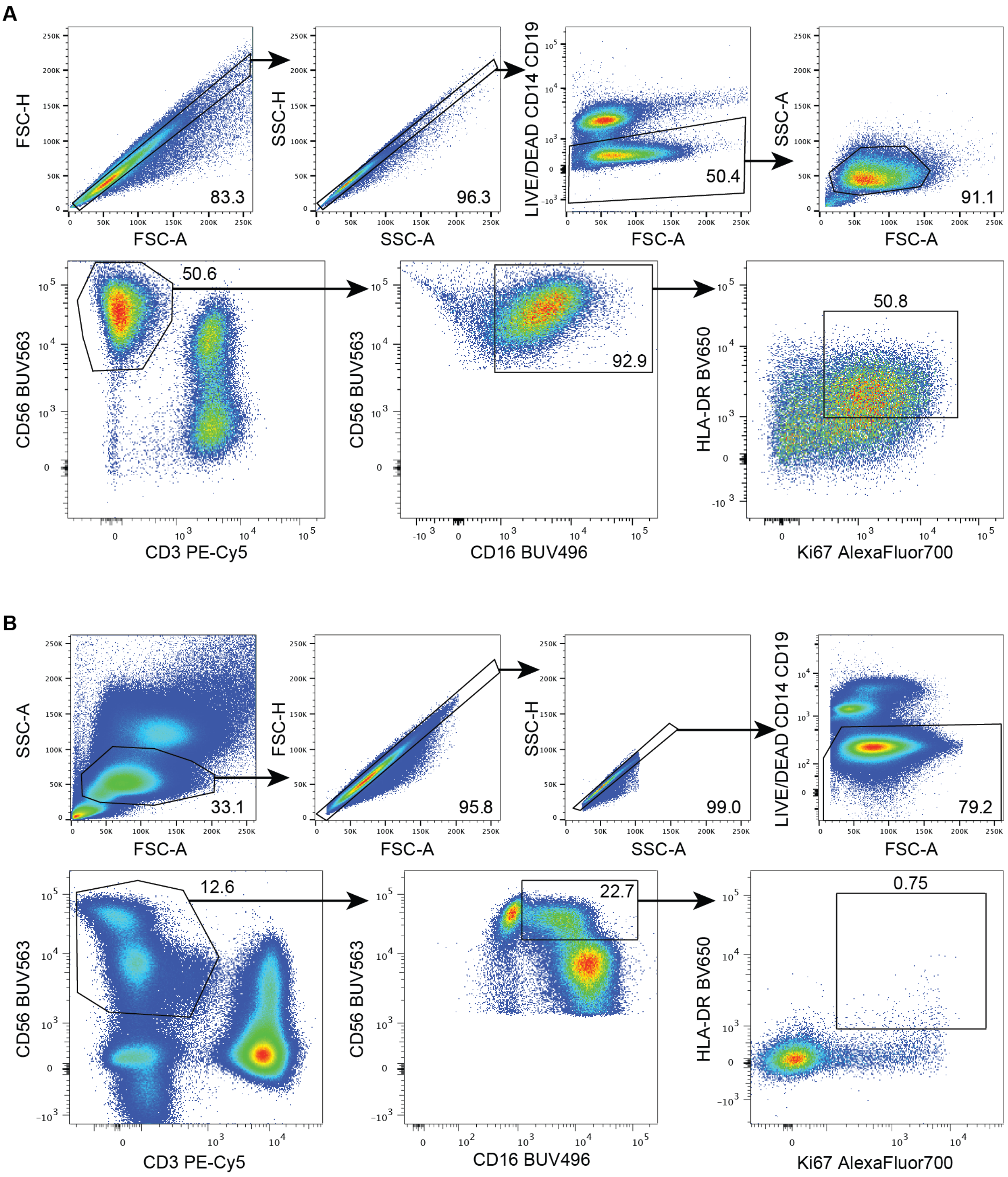

Figure S2. Gating strategy employed to characterize CD56^{bright}CD16^{+}Ki67^{+}HLA-DR^{+} NK cells. Related to Figure 1. Representative plots from one study subject (P107) are shown. **(A)** NK cell product before infusion. **(B)** Study subject PBMC before the first infusion of the NK cell product (same day).

Figure S3

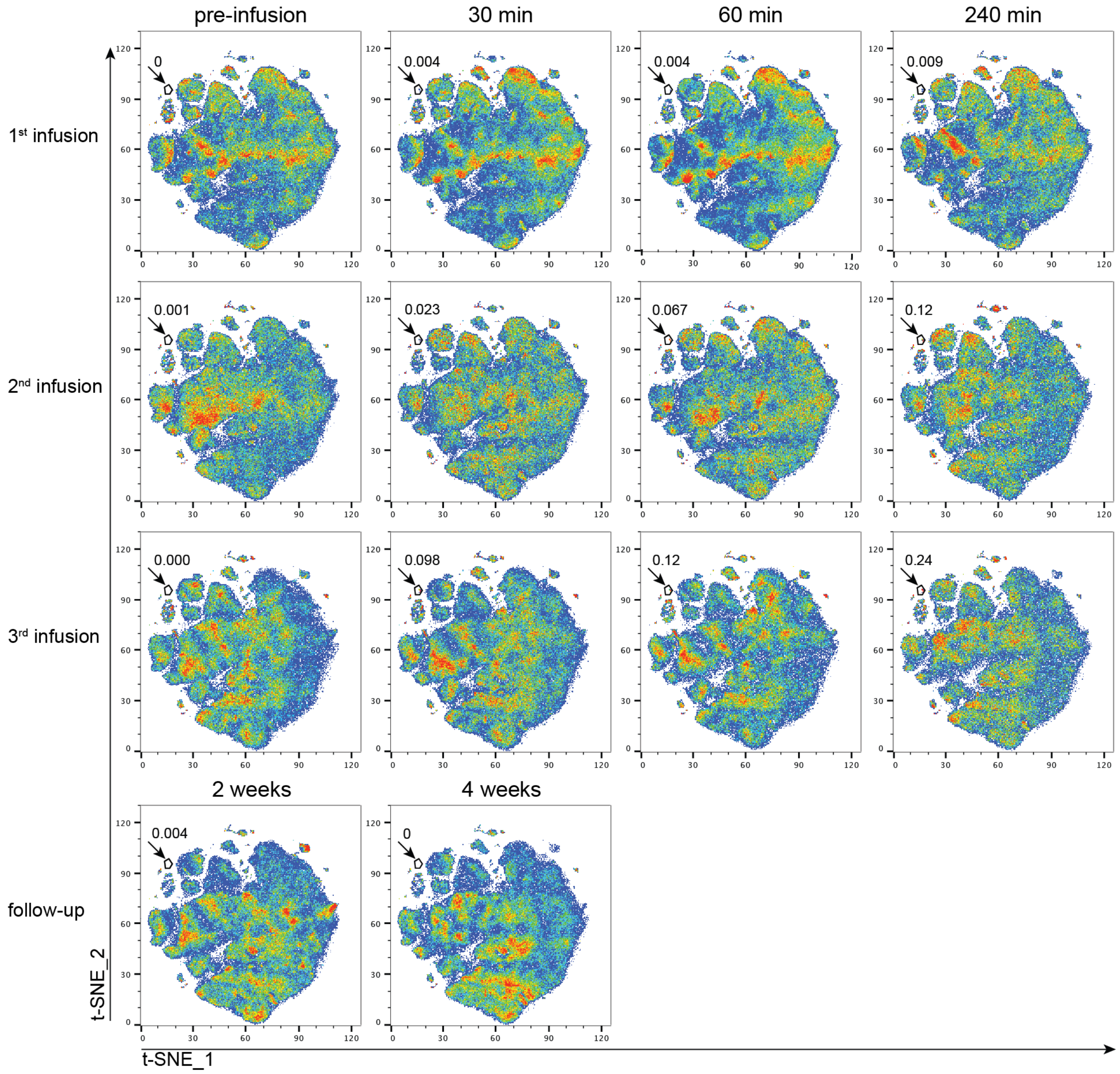

Figure S3. Clustering analysis of data using t-SNE based on 19 markers. Related to Figure 2. Data from $CD56^{+}CD3^{-}CD19^{-}CD14^{-}$ NK cells from all time points were pooled for the calculation. Representative data from one study subject (P103) is shown. The numbers next to the gate represent the percentage of the population within total NK cells at the respective time point.

Figure S4

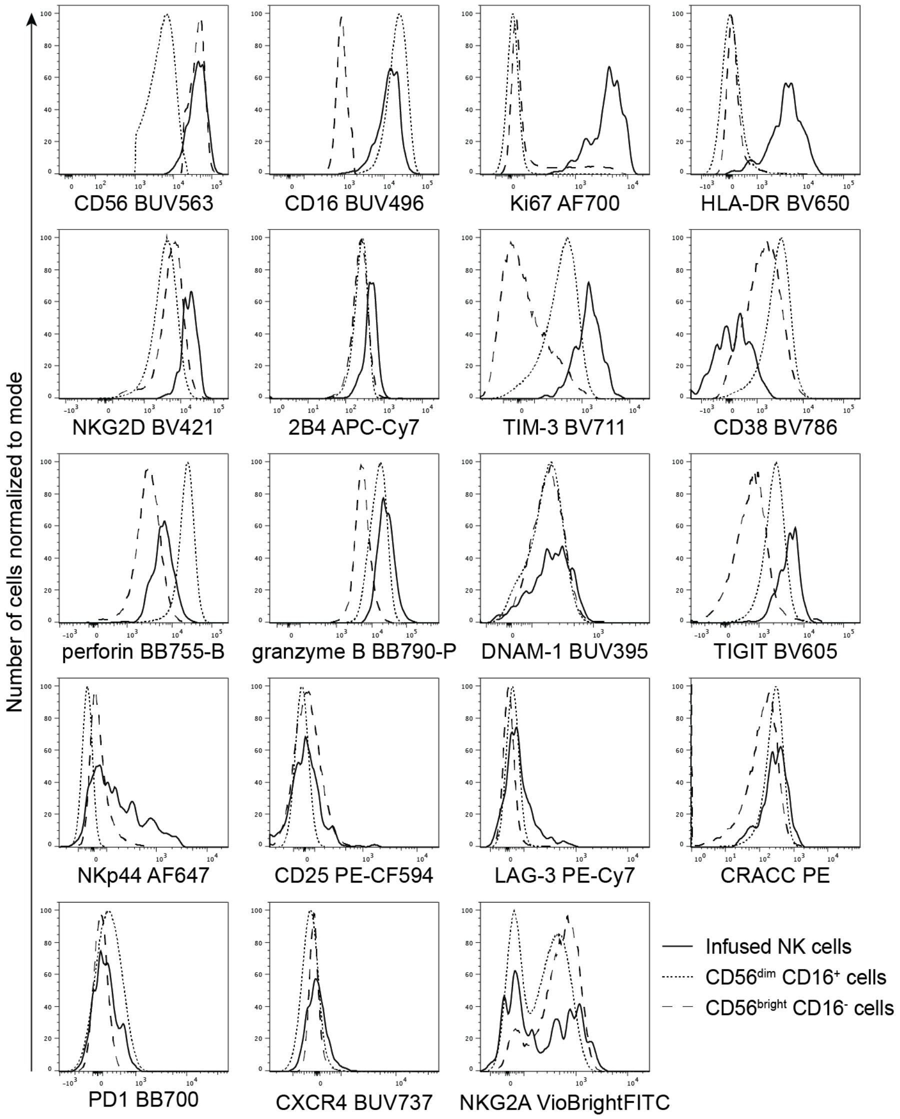

Figure S4. Detailed phenotypic analysis of infused NK cells in comparison to CD56^{dim}CD16^{+} and CD56^{bright}CD16^{-} NK cells in circulation. Related to Figure 2. Representative data from one study subject (P106) is shown. Histograms display data from the respective subpopulations within CD56^{+}CD3^{-}CD19^{-}CD14^{-} NK cells pooled over all time points.

Figure S5

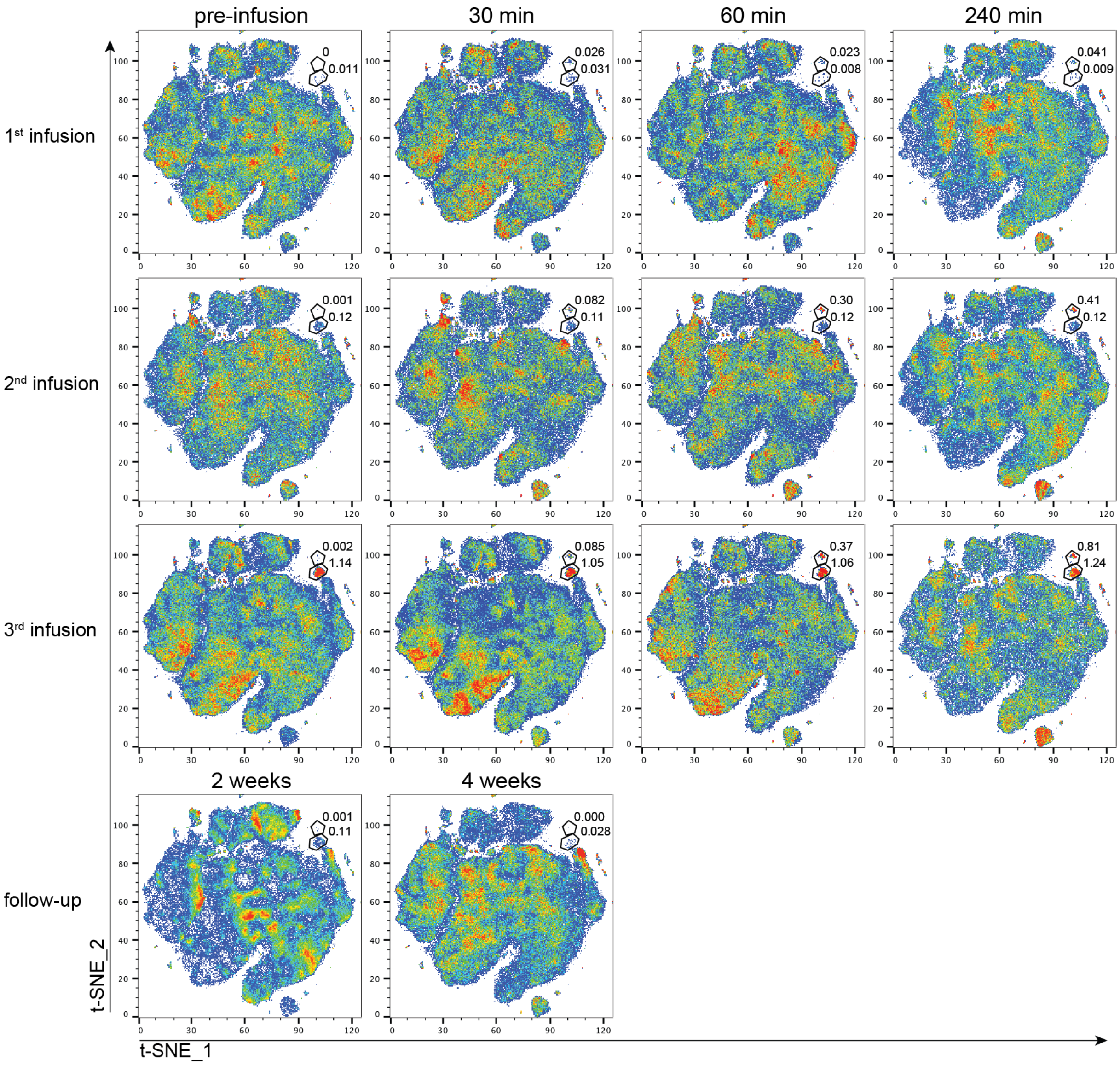

Figure S5. Temporal appearance of infused populations within study subject peripheral blood NK cells. Related to Figure 2. t-SNE plots of data from CD56^{+}CD3^{-}CD19^{-}CD14^{-} NK cells pooled from all time points (study subject P111). The numbers next to the gates represent the percentage of that population within total NK cells at the respective time point.

Supplemental Tables

Table S1. Clinical chemistry at diagnosis and prior to NK cell infusion. Related to Figure 4.

	**At diagnosis**										**Pre-NK cell infusion**				
**Study subject**	**B_2M (mg/L)**	**Albumin (g/L)**	**LDH (ukat/L)**	**Plasma M-spike (g/L)**	**iFLC[a] (mg/L)**	**FLC ratio**	**Urine M-spike (mg/L)**	**Hb (g/L)**	**Creatinine (mmol/L)**		**iFLC[A] (mg/L)**	**FLC ratio**	**Urine M-spike (mg/L)**	**Hb (g/L)**	**Creatinine (mmol/L)**
**P103**	7.1	27	2.3	67	96	9.70	92	96	60		0	0.93	<7.0	94	55
**P105**	4.2	37	2.6	40	58	9.14	33	97	92		4.4	2.90	<7.0	92	66
**P106**	2.4	35	3.4	33	793	193	9.37	127	92		0	0.72	<7.0	136	86
**P107**	-	42	3.9	0	4943	739	5640	95	202		2	1.64	<7.0	116	124
**P110**	3.0	37	4.1	18	1131	126	696	134	78		0	1.03	<7.0	138	81
**P111**	2.5	39	2.2	3[b]	2194	141	1990	115	60		2	1.20	<7.0	111	57

[a] Normal range of FLC-k 6.8-22.4 mg/L

[b] IgD is quantified qualitative, amount may be an underestimation of actual amount

Table S2. **MRD analysis.** Related to Figure 4.

	Read frequency (%)[a]		
**Study subject**	**Diagnosis**	**-2 weeks**	**4 weeks**
**P103**	58.28	0.56	0.25
**P105**	n.c.	n.c.	n.c.
**P106**	54.01	0.033	0.023
**P107**	88.56	b.d.	b.d.
**P110**	n.d.	n.d.	n.d.
**P111**	43.49	0.086	0.052

[a] The read frequency given represents the percentage of the clonal IGH VDJ sequence (identified in the MM diagnosis sample) out of total IGH VDJ sequences.

n.c. no consent to bone marrow sampling

b.d. below detection level; i.e., the frequency of clonal IGH VDJ rearrangements was <1/100 000

n.d. not done

Table S3. Additional study subject information, NK cell dosing, and response status during follow-up.

Related to Figure 4.

Study subject	Response status after ASCT[a]	Time from ASCT to infusions (weeks)	NK cell product doses (10^6 cells/kg)	Response status during six months follow-up	Time from inclusion to progression (months)
**P103**	VGPR	6; 7; 8	5; 50; 100	VGPR	27
**P105**	VGPR	12; 13; 14	5; 50; 100	Relapse at 5 months	15
**P106**	CR	15; 16; 17	5; 50; 52[b]	CR	30
**P107**	VGPR	15; 16; 18	5; 50; 100	CR	>59[c]
**P110**	CR	14; 15; 16	5; 50; 40[b]	CR	>50[c]
**P111**	CR	17; 18; 19	5; 50; 69[b]	CR	38

VGPR, very good partial response; CR, complete remission

[a] ASCT: autologous hematopoietic stem cell transplantation

[b] The third dose was reduced due to scarcity of cells

[c] Study subjects have not progressed at the time of submission

Table S4. **Treatment-emergent adverse events (TEAE).** Related to Figure 5.

Study subject P103

ICD10	TEAE	Manifestation time (days from respective infusion)	Duration (days)	Severity	Causality
R53.9	Malaise and Fatigue	Inf1+0	<1	mild	probable
R11.9A	Nausea	Inf1+0	<1	mild	unlikely
R51.9	Headache	Inf1+1	<1	mild	unlikely
M79.1	Myalgia, legs	Inf1+1	34	mild	possible
M79.1G	Myalgia, legs	Inf1+1	34	moderate	unlikely
M54.2	Neck pain	Inf1+6	29	mild	unlikely
R53.9	Malaise and Fatigue	Inf2+0	<1	mild	unlikely
R53.9	Hot flash	Inf2+0	1	mild	unlikely
J30.4	Rhinitis	Inf2+0	6	mild	unlikely
R53.9	Sickness sensation	Inf3+0	<1	mild	unlikely
R20.2	Paresthesia in toes	Inf3+0	<1	mild	unlikely
R51.9	Headache	Inf3+0	1	mild	unlikely
F32.0	Depression	Inf3+14	n.a.	moderate	unlikely
M54.5	Lumbago	Inf3+78	n.a.	mild	unlikely
B02.9	Shingles, back	Inf3+124	21	moderate	possible
M54.4	Lumbago	Inf3+124	58	mild	unlikely
R50.9	Fever	Inf3+129	1	mild	unlikely
B02.9	Shingles in the groin	Inf3+159	7	moderate	possible

Study subject P105

ICD10	TEAE	Manifestation time (days from respective infusion)	Duration (days)	Severity	Causality
D64.9	Anemia	Inf2+0	1	mild	unlikely
B02.9	Shingles	Inf3+30	5	moderate	possibly
J06.9	Upper respiratory Infection	Inf3+48	16	mild	unlikely
H04.1	Dry eyes	Inf3+90	n.a.	mild	unlikely
J06.9	Upper respiratory infection	Inf3+128	18	moderate	unlikely
D64.9	Anemia	Inf3+146	6	mild	unlikely

Study subject P106

ICD10	TEAE	Manifestation time (days from respective infusion)	Duration (days)	Severity	Causality
R49.0	Dysphonia	Inf1+3	<1	mild	unlikely
R49.0	Dysphonia	Inf1+3	17	mild	unlikely
B02.9	Shingles	Inf3+2	25	moderate	probable
J06.9	Upper respiratory infection	Inf3+56	7	mild	unlikely
M54.5	Lumbago	Inf3+143	3	mild	unlikely

Study subject P107

ICD10	TEAE	Manifestation time (days from respective infusion)	Duration (days)	Severity	Causality
B02.9	Shingles	Inf2+0	13	moderate	possible
K59.1	Diarrhea	Inf2+15	2	mild	unlikely
M54.5	Lumbago	Inf3+6	9	mild	unlikely
K59.1	Diarrhea	Inf3+28	29	mild	unlikely
R20.8	Paresthesia	Inf3+143	40	mild	unlikely

Study subject P110

ICD10	TEAE	Manifestation time (days from respective infusion)	Duration (days)	Severity	Causality
R51.9	Headache	Inf3+1	<1	mild	unlikely
J06.9	Upper respiratory infection	Inf3+	n.a.	mild	unlikely
L57.0	Facial actinic keratosis	Inf3+	n.a.	mild	unlikely
D22.3	Atypical (Dysplastic) melanocytic mole right side of neck	Inf3+	n.a.	mild	unlikely

Study subject P111

ICD10	TEAE	Manifestation time (days from respective infusion)	Duration (days)	Severity	Causality
R25.8	Restless legs	Inf1+0	<1	mild	unlikely
K59.1	Diarrhea	Inf1+6	<1	mild	unlikely
R50.9	Fever	Inf3+4	5	mild	unlikely
R05.9	Cough	Inf3+4	4	mild	unlikely
J06.9	Upper respiratory infection	Inf3+9	20	mild	unlikely
J06.9	Upper respiratory infection	Inf3+53	87	mild	unlikely

Table S5. NK cell product stability. Related to STAR method *Generation of* good manufacturing practice (*GMP) ex vivo activated and expanded NK cells for the clinical study*

	**Dose 1**			**Dose 2**				**Dose 3**	
**Storage time at -180°C (months)**	0	19		0	9	114		0	114
**Viability[a] (%)**	99.0	98.7		99.0	98.2	95.8		99.0	93.5
**NK cells[b] (%)**	84.1	81.5		84.1	83.3	84.4		84.1	84.8
**Degranulation[c] (%)**	80.0	81.0		80.0	76.0	79.1		80.0	84.0

[a] Assessed by trypan blue exclusion

[b] Defined as $CD45^+CD56^+CD3^-$ in flow cytometry

[c] $CD107a^+$ NK cells after co-incubation with K562 cell line

Methods S1. Study protocol synopsis, related to STAR methods *Experimental Model and Subject Details*

PROTOCOL SYNOPSIS

**BASICS**	**EudraCT No**<br>2010-022330-83<br>**Project Identifier**<br>ACP-001<br>**Investigational Product**<br>CellProtect; Ex vivo expanded and activated Natural killer (NK) cells<br>**Development phase**<br>Phase I, first-in-human, therapeutic exploratory<br>**Indication**<br>Multiple Myeloma (MM)<br>**Design**<br>Open, single arm, triple dose study<br>**Number of participating investigator sites**<br>One (1)
**ADMINISTRATIVE STRUCTURE**	**Sponsor**<br>Department of Hematology<br>Karolinska University Hospital<br>M54<br>SE-141 86 Stockholm<br>**Manufacturer of the Investigational Product**<br>Vecura<br>Clinical Research Centre<br>Karolinska University Hospital<br>SE-141 86 Stockholm<br>**Principal Investigator**<br>Dr. Hareth Nahi<br>Department of Hematology<br>Karolinska University Hospital<br>M54<br>SE-141 86 Stockholm
**OBJECTIVES**	**Primary objective**<br>• To investigate the safety and tolerability of CellProtect in patients with MM following Autologous Stem Cell Transplantation (ASCT).

Secondary objectives

- To investigate the effect of CellProtect on monoclonal immunoglobulin levels.
- To investigate the effect of CellProtect on free light chain in serum.
- To investigate the effect of CellProtect on plasma cell fraction in bone marrow.
- To investigate the effect of CellProtect on the International Myeloma Working Group uniform response criteria.

POPULATION

Planned number of patients

Considerable withdrawal rate is expected due to the nature of the disease and the following approximate patient recruitment progress is estimated:

20 blood donations
15 commencing study treatment
12 completed

Inclusion criteria

1) Signed Informed Consent
2) 18 to 70 years of age
3) MM, diagnosed according to Greipp PR, San Miguel J, Durie BG, et al. (2005) as having both
   a) Clonal plasma cells in a bone marrow sample
   b) Measurable monoclonal immunoglobulins in plasma or urine
4) Eligible for, and willing to undergo, high dose chemotherapy and ASCT
5) Eastern Cooperative Oncology Group (ECOG) performance status 0-2
6) Life expectancy of at least three months

Exclusion criteria

1. Non-secretory MM
2. Active malignancy, other than MM
3. Blood donation or other significant blood loss within three months from screening
4. Haemoglobin in blood < 80 g/L
5. Any Related Organ or Tissue Impairment (ROTI), as defined by the International Myeloma Working Group (2003), requiring emergency treatment
6. Known or suspected allergic reactions to any ingredient of the IP

7. Diagnosis or indication of any active autoimmune disease, such as Rheumatoid Arthritis, Inflammatory Bowel Disease, Systemic Lupus Erythematosis or Multiple Sclerosis
8. Uncontrolled or severe cardiovascular disease, such as myocardial infarction within six months from screening, heart failure (class III or IV according to New York Heart Association), uncontrolled angina, clinically significant pericardial disease or cardiac amyloidosis
9. Poorly controlled hypertension, defined as blood pressure that remains above goal in spite of the concurrent use of 3 antihypertensive agents of different classes
10. Poorly controlled Diabetes Mellitus, type I or II, defined as screening results for HbA1c of >63 mmol/mol (IFCC)
11. Renal insufficiency manifested by plasma creatinine > 300 µmol/L and/or by the need for dialysis
12. Diagnosis or indication of any clinically relevant hepatic disease, where indication is defined as screening results (plasma) for either
    a. ALAT >1.2 µkat/L (women) and >1.8 µkat/L (men)
    b. ALP >2.8 µkat/L
    c. ASAT >0.92 µkat/L (women) and >1.14 µkat/L (men)
    d. Bilirubin >30 µmol/L
    e. GGT >1.14 µkat/L (women < 41 years), >1.95 µkat/L (women ≥ 41 years), >2.1 µkat/L (men < 41 years) and >3.0 µkat/L (men ≥ 41 years)
13. Clinically relevant ongoing infection, as judged by the investigator
14. Vaccination with any living vaccine within three months from screening
15. Positive for HIV or Hepatitis B/C
16. Known or suspected drug or alcohol abuse, within 12 months from screening
17. Pregnant, trying to become pregnant, or nursing
18. Lack of, or unreliable contraceptive method, as judged by the Investigator
19. Medical history or any abnormal physical finding that is clinically relevant and could interfere with the safety or objectives of the study, as judged by the Investigator
20. Lack of suitability for participation in the trial, for any reason, as judged by the Investigator

	**Withdrawal criteria**<br>• It is the expressed wish of the patient<br>• It is medically necessary, as judged by the Investigator<br>• The Investigational Product (IP) is, or very likely will be, insufficient for at least the first two infusions<br>• The first infusion is not performed within six months from the ASCT<br>• Pregnancy or trying to become pregnant
**STUDY CONDUCT**	**Duration of a patient's participation**<br>Between approximately 12 to 18 months<br>**Number of study visits**<br>Sixteen (16)<br>**Description**<br>After being included in the study, a patient will first donate blood for the production of the IP.<br>Subsequent to the blood donation, the patient will be treated according to current clinical praxis with chemotherapy (typically for two to four months) followed by ASCT.<br>The study treatment should then be initiated within six months from the ASCT, where the time point for first infusion is chosen with consideration to the patient's physical condition; The study treatment cannot start as long as the patient has an unstable or poor condition.<br>When the patient is sufficiently well and stable after the ASCT, the patient will receive three infusions with IP, with an interval of eight days.<br>Safety and efficacy parameters are followed from first infusion until six months from last infusion.<br>**Safety precaution at infusion visits**<br>Any two patients must not be treated on the same day.<br>Furthermore, sequential treatment of patients must be applied until at least two patients have received all three infusions according to the protocol, and the accumulated number of activated NK cells for at least one of the patients adds up to no less than 9.3×10^6 cells/kg body weight. Patients in sequential treatment must be separated by a minimum of six days.<br>Every infusion during the sequential treatment period must be preceded by a safety evaluation, where the Investigator reviews all relevant laboratory analyses data, vital signs, ECG data and AEs for

previous infusions. The Investigator must consider it safe to proceed before the infusion can take place.

Occurrence of any acute reactions are monitored by measuring the body temperature and vital signs 15, 30, 45 (not temperature) minutes and 1, 2, 4, 6 and 24 hours after start of the infusion, as well as ECG recording 6 and 24 hours after start of the infusion. AEs are continuously monitored up until discharge.

TREATMENTS

Description

The IP is a cell suspension based on ex vivo expanded NK cells from patients with MM. The treatment is strictly autologous. The IP is given as three infusions with escalating doses.

The cell expansion protocol includes stimulation and selection of NK cells ($CD3^{-}CD56^{+}$), which are expected to be the most cytotoxic to tumour cells.

The product is individually prepared and provided in bags, where the volume and concentration of cells depend on the intended dose and the expansion yield. The total volume for each dose level is always within the range of 10 to 200 ml. The contents of each bag are drawn up in a syringe and administered as i.v. infusion.

In this study protocol, the expression “investigational product” is synonymous with the expression “Advanced Therapy Investigational Medicinal Product” used in the “Detailed guideline on good clinical practice specific to advanced therapy medicinal products”

Mode of administration

Intravenous infusions.

Dose levels

- First infusion; $5x10^{6}$ cells/kg body weight
- Second infusion; $50x10^{6}$ cells/kg body weight
- Third infusion; $100x10^{6}$ cells/kg body weight

A dose range of ±10 % is acceptable for each dose level. The doses refer to the total number of cells in the preparation before cryopreservation. For a per protocol treatment to be achieved, at least 6 % of the total number of cells should be activated NK cells.

If there is a scarcity of material only two infusions may be given. The third infusion should only be given if the available dose is equal or higher than that of the second infusion.

ASSESSMENTS

Safety

- Weight
- Physical examination
- Vital signs

	• Body temperature<br>• ECG<br>• Laboratory analyses, including:<br>o Standard routine safety analyses of blood and urine<br>o Cytokines IL-2, IL-6, IL-10 and TNF-α in blood<br>• Adverse Events<br>**Efficacy**<br>• The International Myeloma Working Group uniform response criteria<br>• Laboratory analyses, including:<br>o Monoclonal immunoglobulin levels in serum and urine<br>o Free light chain in serum<br>o NK cell count, NK phenotype and plasma cell fraction in bone marrow<br>• Blood and bone marrow samples from consenting patients are saved for future explorative analyses
**STATISTICS**	The sample size of 12 completed patients is based on clinical praxis where sample sizes between 6 to 12 subjects are commonly used for similar safety studies.<br>The study is analysed by descriptive statistics only. Continuous variables are described by summary statistics, i.e. number of observations, mean, standard deviations, medians and range (minimum and maximum values). Categorical variables are summarised in frequency tables as counts and percentages. Graphs are generated when appropriate.<br>Baseline is defined as the status at pre-dose first infusion.
**PROTOCOL**	Version 9.0, dated 03DEC2012
