# Supplementary material for: Autologous NK cells as consolidation therapy following stem cell transplantation in multiple myeloma
Source: Cell Rep Med. 2022 Jan 28;3(2):100508. doi: 10.1016/j.xcrm.2022.100508 (PMC8861830; doi:10.1016/j.xcrm.2022.100508)
Supplement: Document S2. Article plus supplemental information [file mmc2.pdf]

Cell Reports
Medicine

Article

Autologous NK cells as consolidation therapy following stem cell transplantation in multiple myeloma

Graphical abstract

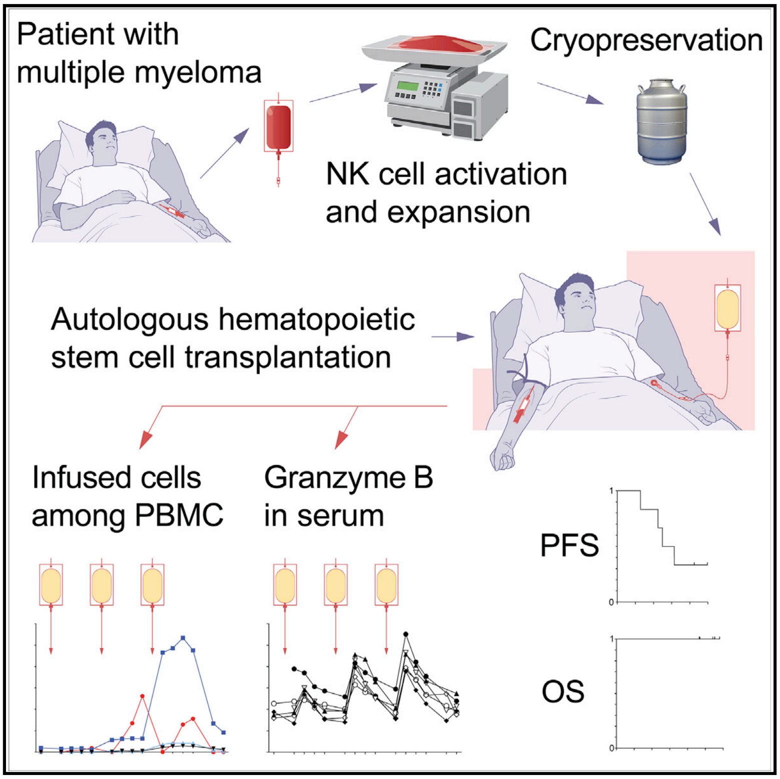

Authors

Hareth Nahi, Michael Chrobok, Stephan Meinke, ..., Per Ljungman, Hans-Gustaf Ljunggren, Evren Alici

Correspondence

evren.alici@ki.se

In brief

Activated and expanded autologous NK cells were repeatedly infused in patients with multiple myeloma following autologous HSCT. The study by Nahi et al. shows safety and demonstrates efficacy parameters. It opens up the possibility to use autologous NK cells in clinical settings, either alone or as parts of combination therapies.

Highlights

- Infusing activated and expanded autologous NK cells in patients with MM is possible
- Infused NK cells are detected in circulation for up to 4 weeks
- Elevated granzyme B levels are observed following each consecutive NK cell infusion
- Objective, detectable responses after NK cell infusions are seen in patients

Nahi et al., 2022, Cell Reports Medicine *3*, 100508
February 15, 2022 © 2022 The Authors.
https://doi.org/10.1016/j.xcrm.2022.100508

CellPress

Cell Reports Medicine

CellPress
OPEN ACCESS

Article

Autologous NK cells as consolidation therapy following stem cell transplantation in multiple myeloma

Hareth Nahi,[1,2] Michael Chrobok,[1,14] Stephan Meinke,[1,14] Charlotte Gran,[1,3] Nicole Marquardt,[4] Gabriel Afram,[1,2] Tolga Sutlu,[1,15] Mari Gilljam,[1] Birgitta Stellan,[1] Arnika K. Wagner,[1] Pontus Blomberg,[5,6] Per-Henrik Holmqvist,[6] Lilian Walther-Jallow,[7] Karin Mellström,[8] Johan Liwing,[8] Charlotte Gustafsson,[9] Robert Månsson,[9] Monika Klimkowska,[10,11] Gösta Gahrton,[1] Johan Lund,[1,2] Per Ljungman,[12,13] Hans-Gustaf Ljunggren,[4] and Evren Alici[1,16,*]

[1]Center for Hematology and Regenerative Medicine, Department of Medicine Huddinge, Karolinska Institutet, SE-14183 Huddinge, Sweden
[2]Department of Hematology, Karolinska University Hospital, SE-14186 Huddinge, Sweden
[3]Department of Clinical Chemistry, Karolinska University Laboratory, SE-14183 Huddinge, Sweden
[4]Center for Infectious Medicine, Department of Medicine Huddinge, Karolinska Institutet, SE-14183 Huddinge, Sweden
[5]Vecura, Department of Laboratory Medicine, Karolinska Institutet, SE-14186 Stockholm, Sweden
[6]Vecura, Karolinska Cell Therapy Center, Karolinska University Hospital, SE-14186 Stockholm, Sweden
[7]Department of Clinical Science, Intervention and Technology, Karolinska Institutet, SE-14183 Huddinge, Sweden
[8]XNK Therapeutics AB, Hälsovägen 7, Novum, SE-14157 Huddinge, Sweden
[9]Center for Hematology and Regenerative Medicine, Division of Clinical Immunology and Transfusion Medicine, Department of Laboratory Medicine, Karolinska Institutet, SE-14183 Huddinge, Sweden
[10]Pathology Unit, Department of Laboratory Medicine, Karolinska Institutet, SE-14183 Huddinge, Sweden
[11]Department of Clinical Pathology and Cytology, Karolinska University Hospital, SE-14186 Huddinge, Sweden
[12]Department of Cellular Therapy and Allogeneic Stem Cell Transplantation, Karolinska University Hospital Huddinge, SE-14186 Huddinge, Sweden
[13]Division of Hematology, Department of Medicine Huddinge, Karolinska Institutet, SE-14183 Huddinge, Sweden
[14]These authors contributed equally
[15]Present address: Department of Molecular Biology and Genetics, Boğaziçi University, 34342 Bebek/Istanbul, Turkey
[16]Lead contact
*Correspondence: evren.alici@ki.se
https://doi.org/10.1016/j.xcrm.2022.100508

SUMMARY

Few approaches have been made toward exploring autologous NK cells in settings of cancer immunotherapy. Here, we demonstrate the feasibility of infusing multiple doses of *ex vivo* activated and expanded autologous NK cells in patients with multiple myeloma (MM) post-autologous stem cell transplantation. Infused NK cells were detected in circulation up to 4 weeks after the last infusion. Elevations in plasma granzyme B levels were observed following each consecutive NK cell infusion. Moreover, increased granzyme B levels were detected in bone marrow 4 weeks after the last infusion. All measurable patients had objective, detectable responses after NK cell infusions in terms of reduction in M-component and/or minimal residual disease. The present study demonstrates that autologous NK cell-based immunotherapy is feasible in a setting of MM consolidation therapy. It opens up the possibility for usage of autologous NK cells in clinical settings where patients are not readily eligible for allogeneic NK cell-based immunotherapies.

INTRODUCTION

In recent years, a plethora of allogeneic natural killer (NK) cell-based immunotherapy trials have been reported with promising results.[1–6] The rationale for using allogeneic NK cells was initially a consequence of insights into the molecular specificity of NK cells, in particular their capacity to mediate missing self-reactivity.[7,8] Applicability was further supported by findings on the role of NK cells in haploidentical hematopoietic stem cell transplantation (HSCT).[9,10] The "off-the-shelf" applicability of allogeneic NK cells has recently further boosted an interest in their clinical use.[6,11–15] As a result, allogeneic NK cell-based immunotherapy strategies currently dominate the field of adoptive NK cell-based cancer immunotherapy (see, e.g., Tschan-Plessl et al. 2021).[16]

In contrast, few studies currently encompass the use of autologous NK cells. As such, the concept of using autologous NK cells, however, is not new. Adoptive transfer of autologous NK cells to patients with cancer originates from studies in the mid-1980s.[17] Yet, these and a few other attempts with autologous NK cells were met with mixed results at best,[18] at least in part due to the clinical setting in which they were used. For example, clinical treatment of patients with refractory solid tumors, such

This is an open access article under the CC BY license (http://creativecommons.org/licenses/by/4.0/).

Table 1. Patients infused with the investigational NK cell-based product

Study subject	Gender	Performance status[a]	Stage of disease[b]	Type of M component	Risk group according to FISH[c]	Bone lesion	BM plasma cells at diagnosis, %
P103	F	1	III/II	IgGκ	SR	Yes	15
P105	F	1	II/II	IgGκ	SR	Yes	60
P106	M	0	II/II	IgGκ	HR	Yes	47
P107	M	1	-[d]	κ[e]	HR	Yes	39
P110	M	1	II/II	IgAκ	SR	Yes	97
P111	M	1	I/I	IgDκ	SR	Yes	18

[a]Performance status grouped according to Eastern Cooperative Oncology Group (ECOG)[26].
[b]International staging system (ISS)[27]/revised international staging system (R-ISS)[28].
[c]SR/HR denoted standard/high risk as classified by the International Myeloma Working Group (IMWG),[29] fluorescence *in situ* hybridization (FISH).
[d]Staging not possible due to lack of B_2M analysis at diagnosis.
[e]Light-chain kappa.

as progressive stage IV melanoma or renal cancer, was unsuccessful.[19] Despite these results, we have revisited the potential of using activated and expanded autologous NK cells in a refined clinical consolidation setting, where allogeneic NK cells would not readily be applicable and where autologous NK cells potentially could be effective.

We here present results from an investigator-driven first-in-human clinical trial utilizing *ex vivo* activated and expanded NK cells following autologous HSCT in patients with multiple myeloma (MM). The study design included administration of 3 escalating doses of activated and expanded NK cells in weekly intervals for each patient. Clinical results, including safety analysis and surrogate exploratory parameters indicative of effector functions, are presented. Conceptually, these early results open up the possibility for further assessment of autologous NK cell-based immunotherapies in specific clinical settings; e.g., low tumor burden, minimal residual disease (MRD), or consolidation treatment in malignant diseases.

RESULTS

Clinical development of autologous NK cell-based immunotherapy for MM patients

Activated and expanded NK cells display an increased cytotoxic activity against autologous primary MM cells *ex vivo*.[20] This, and results from earlier studies,[21–23] led to the hypothesis that autologous *ex vivo* activated and expanded NK cells could be evaluated in clinical settings. Hence, we designed a clinical study exploring the feasibility and safety of adoptively transferred activated and expanded autologous NK cells to patients with MM. For this study, a feeder-free, GMP-compliant, automated and closed system for activation and expansion of NK cells from patients with malignant diseases was adopted.[24] Based on this protocol, a first-in-human investigator-initiated clinical study was initiated in patients having undergone autologous HSCT for MM. The clinical study was designed as an open, single-arm study. The primary objective was to assess safety and tolerability of the NK cell-based product. Secondary objectives were to assess disease response, measured by monoclonal immunoglobulin levels, serum-free light chain, and MRD according to the international myeloma working group (IMWG) uniform response criteria.[25] The clinical characteristics of patients treated are summarized in Table 1. Additional patient information is listed in Table S1. The setup of the study is outlined in Figure S1 and described in more detail in Methods S1. In the study, 6 study subjects received 3 escalating doses each of activated and expanded NK cells in weekly intervals.

Flow cytometry-based tracking of infused autologous NK cell product in patients

Upon *ex vivo* activation and expansion, we observed that the NK cells gained a unique activated phenotype that includes populations of CD56[bright]CD16[+]Ki67[+]HLA-DR[+] NK cells (Figure 1A; Figure S2). We hypothesized that this specific phenotype might allow us to detect the infused NK cells among peripheral blood NK cells directly following the infusion to the study subjects. To address this, peripheral blood was collected from the non-NK cell infusion arm (median cubital vein) directly before and at several time points after each consecutive NK cell-based product-infusion (illustrated in Figure 1B). Cells taken from the non-NK cell infusion arm were analyzed by multiparameter flow cytometry. Clustering analyses of data from these samples using t-distributed stochastic neighbor embedding (t-SNE) revealed the gradual appearance of the new NK cell population in a dose-dependent fashion over 4 h following infusion of the NK cell product (Figures 1C and 1D; Figure S3). Furthermore, comparable to the NK cell infusion product, these cells expressed high levels of NKG2D, 2B4, TIM-3, and TIGIT, as well as surprisingly low levels of CD38 (Figure 1E; Figure S4). In 2 of the study subjects, we also observed a gradual appearance of a CD56[dim]CD16[+]Ki67[dim]HLA-DR[dim] NK cell population (Figures 2A, 2B, 2C, and S5), persisting for at least 4 weeks (Figures 2D and 2E). These observations suggest a phenotypic drift following infusion, potentially due to tumor exposure and reduced non-specific inflammatory stimuli such as IL-2.

Plasma proteomic assessment of granzymes and pro-inflammatory cytokines in conjunction with NK cell product infusion

In parallel with the detailed analysis of NK cell subpopulations in peripheral blood, we assessed the plasma proteome in conjunction with each consecutive NK cell product infusion (Figure 3A). With the exception of the first infusion to study subject P103, a

CellPress
OPEN ACCESS

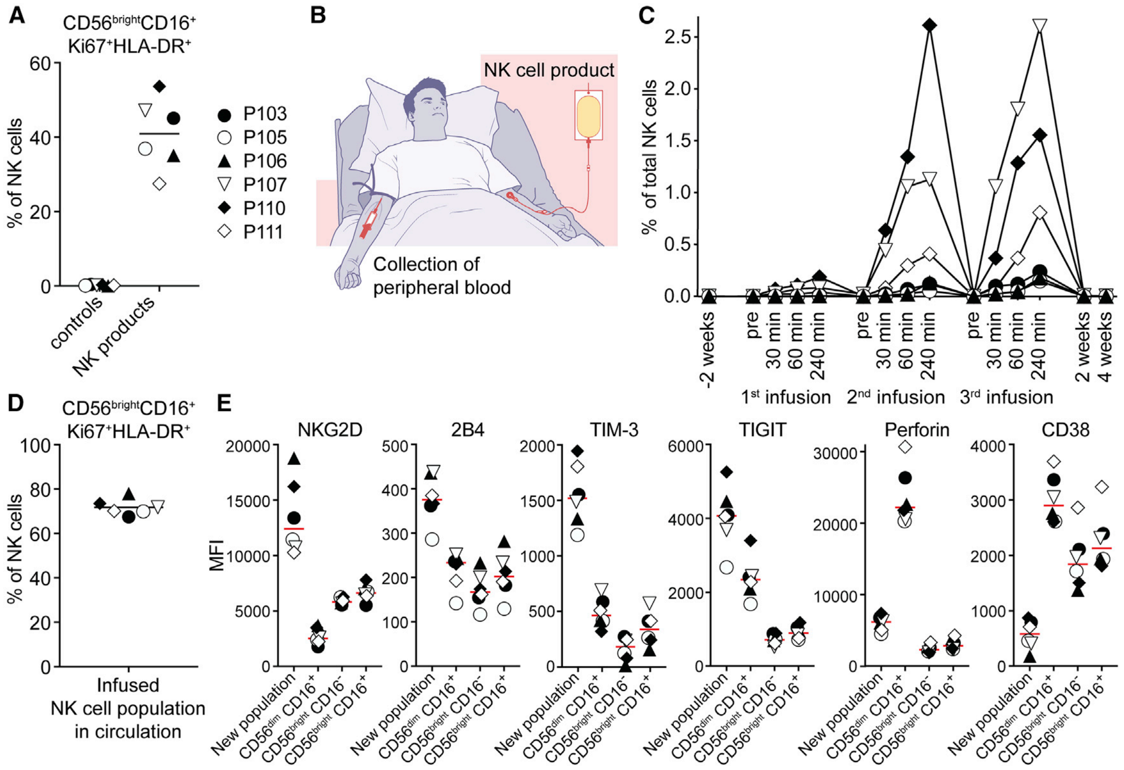

Figure 1. Flow-cytometry-based tracking of infused autologous NK cell product in patients

(A) Percentage of *ex vivo* activated and expanded NK cells with the $CD56^{bright}CD16^{+}Ki67^{+}HLA\text{-}DR^{+}$ phenotype in the NK cell products. Controls represent study subject peripheral blood NK cells before the first infusion. Lines represent the mean values. Symbols represent patients in all panels displayed (n = 6).

(B) Strategy employed to detect *ex vivo* activated and expanded autologous NK cells among peripheral blood NK cells directly following the infusion.

(C) Relative size of a defined subset of the infused NK cell population as detected in the circulation of study subjects after infusion of the NK cell product. Infused NK cells were identified by their distinct phenotype by using t-SNE analysis.

(D) Percentage of NK cells with the $CD56^{bright}CD16^{+}Ki67^{+}HLA\text{-}DR^{+}$ phenotype within the infused cell populations followed in (C). Data shown are pooled from all time points. Line represents the mean value.

(E) Median fluorescence intensities of selected markers on the infused NK cell populations followed in (C) compared with other NK cell subpopulations. Data shown are pooled over all time points. Lines represent the median values.

significant and dose-dependent increase, peaking 30 min after each NK cell-product infusion, was observed for granzyme B (Figure 3B). Similar, but less marked, patterns were observed for granzyme A and granzyme H (Figures 3C and 3D) and the pro-inflammatory molecules CCL3 (MIP1-α) and CCL4 (MIP1-β) (Figure 3A). IL-6 levels peaked at 60–240 min after each infusion (Figure 3D). Absolute values for IL-6 as measured by ELISA, however, did not exceed 17 pg/mL. For other plasma proteins, no or insignificant responses were observed (Figure 3A). Finally, an increase in granzyme B was also observed in plasma from bone marrow aspirates taken 4 weeks after the last infusion (Figure 3F; data shown from 4 study subjects from which bone marrow aspirate material was available for analysis).

Clinical assessment of MM patients following autologous NK cell-based immunotherapy

The clinical effects of the NK cell product infusions were assessed during a 6-month follow-up period. At the time of the first NK cell product infusion, 2 of 6 patients had a detectable M-component. Both of these showed a decrease in M-component following the first NK cell infusion, and additionally, after the completion of the infusion period. In patients with no detectable M-component, whether serum Ig (Figure 4A, left) or free light chains (Figure 4A, right), the M-component remained undetectable throughout the entire infusion period and during the full 6-month follow-up period. In 3 of 4 assessed study subjects, a deepening of MRD was observed 4 weeks after the last infusion (Figure 4B; Table S2). The fourth study subject was MRD negative at the time of infusion and remained MRD negative 4 weeks after the last infusion. Median progression-free survival (PFS) was 34 months (Figure 4C). All six study subjects are still alive, during a minimum 60-month follow-up period (Figure 4D). Additional data, including response status after autologous HSCT, time from autologous HSCT to infusion, NK cell product doses infused, and follow-up response status, are displayed in Table S3.

Safety assessment of MM patients following autologous NK cell-based immunotherapy

The safety of the NK cell product infusions was assessed immediately and for a 6-month follow-up period. No severe adverse

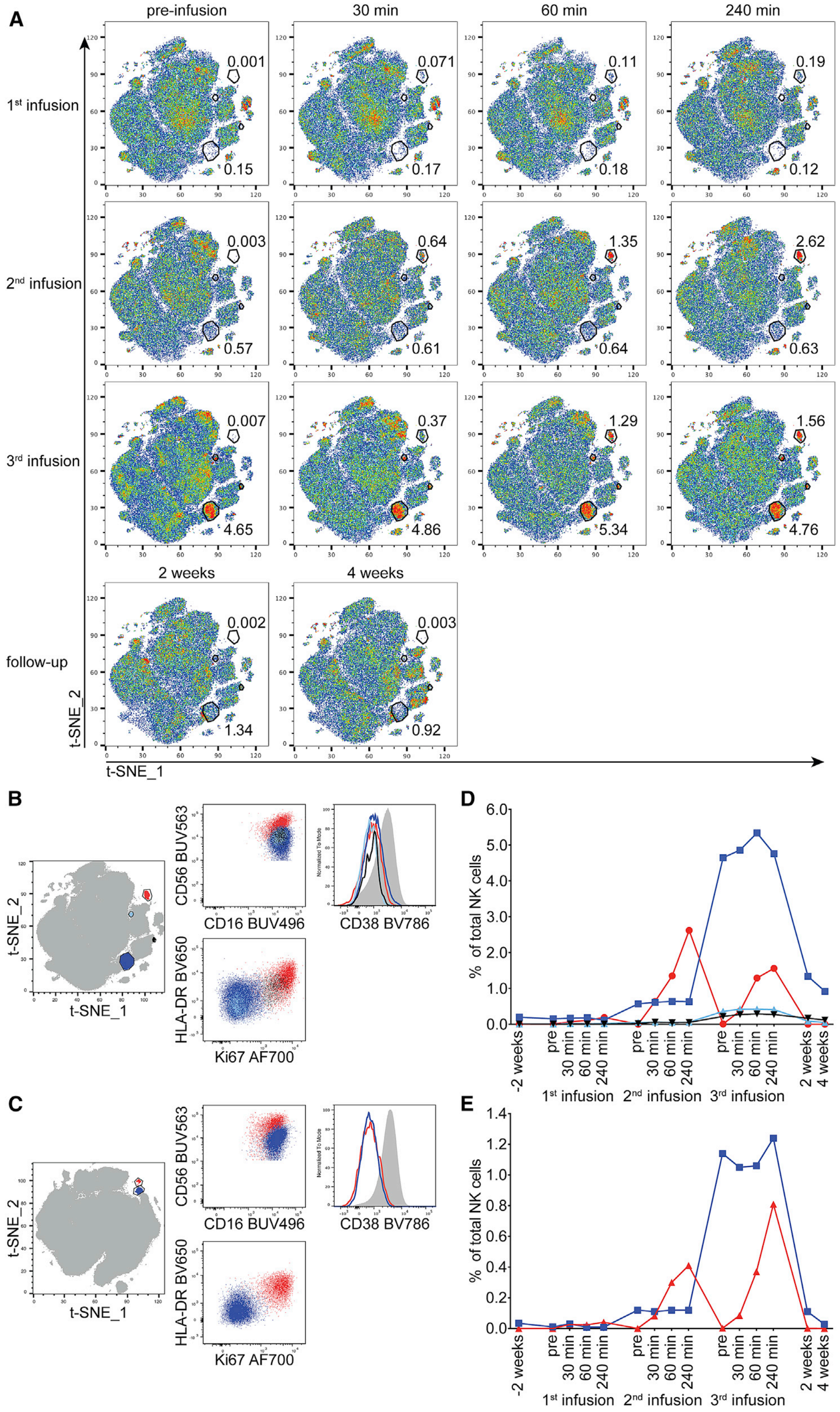

(legend on next page)

events (SAE) were observed. A complete list of treatment-emergent adverse events (TEAE) is displayed in Table S4. Overall, the clinical study demonstrated safety and tolerability. However, unexpectedly, the first 4 study subjects developed herpes zoster (HZ) following the infusion of the NK cell product (Figure 5A). In all cases, HZ development occurred after the antiviral prophylaxis with valacyclovir following the autologous HSCT had ended. In 3 of the 4 study subjects, it manifested after the third NK cell product infusion, and in 1 of the study subjects, it had already manifested at the time of the second infusion (Figures 5B and 5C). Clinically, shingles manifestation was managed with standard antiviral treatment. Upon consultancy with the Swedish Medical Products Agency, the last 2 study subjects were prophylactically treated with valacyclovir (500 mg) twice daily for 6 months from the first NK cell product infusion. No signs of shingles were observed in these 2 study subjects.

DISCUSSION

The present study has explored the potential use of autologous *ex vivo* activated and expanded NK cells in patients with MM. A particular emphasis was on clinical settings where allogeneic NK cells would not readily be applicable and where there would be an unmet need to explore alternative immunotherapy approaches to treatment. On the basis of *ex vivo* and *in vivo* results from experimental MM model systems, we proceeded with a clinical study in MM patients following autologous HSCT. Although autologous HSCT, following conditioning, serves to reduce the tumor burden, all MM patients eventually relapse.[30,31] Hence, in this setting, there is ample reason for the exploration of novel complementary approaches. We here report that escalating doses of *ex vivo* activated and expanded autologous NK cells are well tolerated in MM patients without any SAEs. The 4 of the total 6 patients that had measurable disease following HSCT showed indications of objective, measurable responses to NK cell infusions in terms of reduction in M-component (serum Ig) and/or MRD. Exploratory analyses revealed increased numbers of infused NK cells in patients' peripheral blood and marked elevations in plasma granzyme B correlating to each individual consecutive infusion. Furthermore, increased levels of granzyme B were also detected in bone marrow aspirates 4 weeks after the last infusion.

No SAE, nor any signs of cytokine release syndrome (CRS) were observed. IL-6 presented with only moderate transient fluctuations following the infusion of the NK cell product. With respect to the manifestation of shingles in the first 4 patients, it is unlikely that the autologous HSCT *per se* is responsible for the HZ reactivation observed in 4 consecutive patients, especially given the time from autologous HSCT to shingles development.[32] The mechanism for the HZ activation is unknown, but it is fathomable that it could relate to the infusion of the autologous NK cell product. Noteworthy, the present study protocol was originally designed without antiviral prophylaxis upon NK cell product infusion. We speculate that activated NK cells could have attacked reservoir cells of the varicella zoster virus that, in turn, could have caused a viral reactivation. Corroborating this hypothesis, it has been previously reported that activated NK cells can recognize dorsal root ganglia cells in experimental model systems.[33–35] At the present stage, we conclude that autologous NK cell-based immunotherapy, even when performed without a lymphodepletion regimen, should be combined with adequate prophylactic antiviral treatment.

In this study, we attempted to follow the infused activated NK cell population, something that turned out to be feasible in the current clinical context, given the specific features of the NK cell product. The rationale for this approach was the fact that the fate of these cells was unclear. Additionally, the phenotypic dynamics of the infused cell population was equally unclear. The infused cells were traceable for a period of 4 weeks and retained a $CD16^{+}CD38^{dim}$ phenotype, a feature that may enable future combinatorial therapies.

While there is a strong rationale for the use of allogeneic NK cell-based immunotherapies, there might be time to revisit the partially omitted field of autologous NK cell-based therapies. Most importantly, the latter may fill a space not easily covered by allogeneic NK cell-based therapies. We base this on the following arguments. Efficient and cost-effective *ex vivo* activation and expansion protocols for human NK cells have now been developed, including the feeder-free protocols as used here. Current protocols allow efficient activation and expansion of NK cells also from patients with malignant diseases.[20,36] As verified for cells undergoing activation and expansion using the protocol employed here, long-term storage of patients' own activated and expanded NK cells is feasible with indications of more than 10 years of stability. The latter may have implications for the development of future therapies. For example, it opens up the possibility for multiple dosing over long periods of time. Importantly, autologous NK cells also allow for infusion to the patient without immunosuppressive conditioning. Possible limitations with respect to autologous NK cell-based products are the obvious lack of off-the-shelf availability, recurring manufacturing costs, and rapid accessibility of the potential therapeutic product.

In adoptive NK cell-based immunotherapy, antitumor activity by NK cells depends on their degree of education/licensing, a process predominantly determined by their interactions with self major histocompatibility complex (MHC) class I molecules on other cells.[37–39] Given this fact, the administration of NK cells to an autologous host sets the stage for an optimal licensing process via NK cell inhibitory receptors interacting with self MHC

Figure 2. Temporal appearance and phenotype of infused populations within study subject peripheral blood NK cells

(A) The temporal appearance of infused populations within the study subject peripheral blood NK cells. Representative t-SNE analysis based on 19 markers of 1 study subject (P110) is shown. The numbers next to the gates represent the percentage of that population within total NK cells at the respective time point. Two populations with different kinetics of appearance are marked (the population on the top right is included in Figure 1C).

(B–E) (B and C) Comparison of the phenotypes. (D and E) The relative sizes of the infused NK cell populations detected in the circulation after infusion of the NK cell product for study subject P110 (B and D) and study subject P111 (C and E). The color coding in the t-SNE plots on the left represents the populations in the graphs to the right.

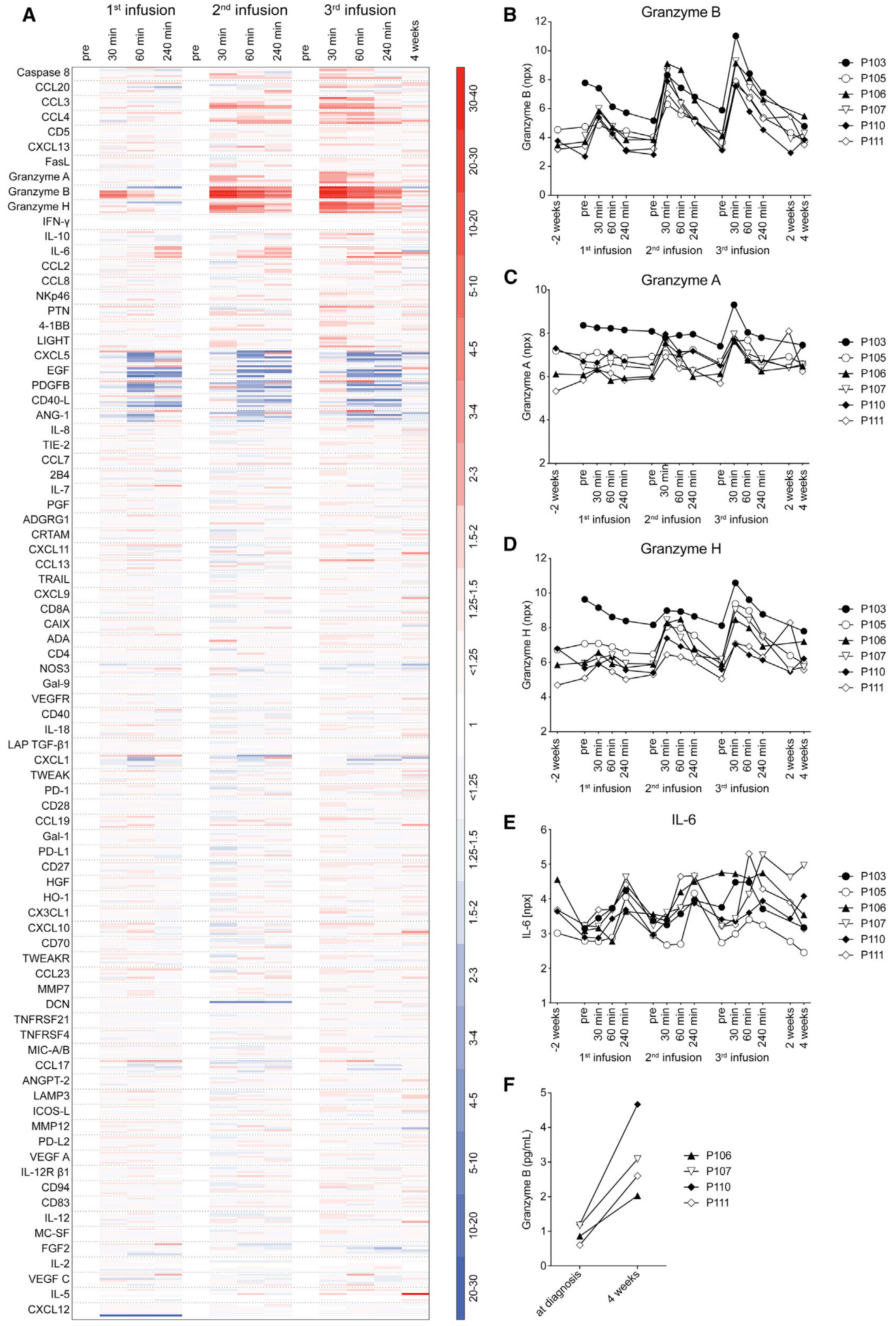

(legend on next page)

CellPress
OPEN ACCESS

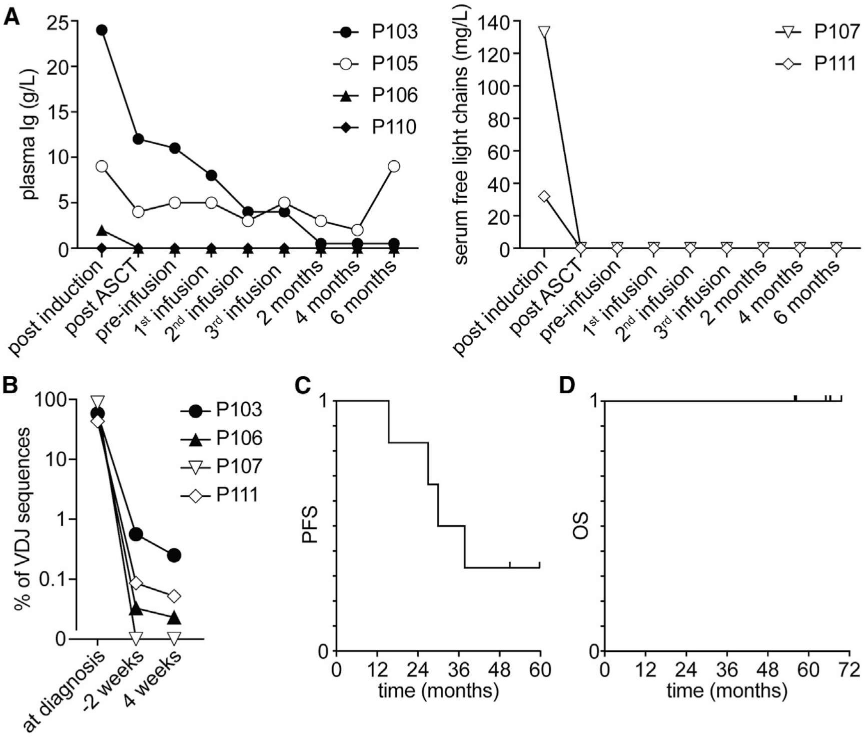

Figure 4. Outcome of autologous NK cell-based immunotherapy for consolidation treatment of patients with multiple myeloma (MM)

(A) Dynamics of plasma M-component (left, n = 4) and serum-free light chains (right, n = 2) in study subjects in the course of the clinical study. ASCT, autologous stem cell transplant.

(B) IgH variability, diversity, and joining(VDJ) rearrangement analysis of BM samples taken at diagnosis and respective MRD values 2 weeks before the first and 4 weeks after the last infusion of the NK cell product. Percentages of the clonal IgH VDJ sequence (as identified in MM diagnosis samples) out of total IgH VDJ sequences are displayed. Data from 4 of 6 study subjects are shown.

(C and D) (C) PFS and (D) OS of all study subjects (n = 6) during the course of the study calculated from the time of inclusion.

class I. That is, a process allowing for optimal recognition of “missing self;” i.e., specific tumor target cells lacking some or all self MHC class I molecules.[40] In this context, there is abundant evidence demonstrating partial or complete loss of histocompatibility leukocyte antigen (HLA) class I expression in a wide spectrum of human tumor types.[41,42] Such loss may result from the immune selection by CD8$^+$ T cells and has also more recently been linked to acquired resistance to checkpoint inhibition therapy.[43] Deduced from the latter studies, and the early predictions of the missing self-concept, autologous NK cell-based adoptive immunotherapy may offer a possible treatment strategy in MHC class I-deficient and, hence, T cell checkpoint inhibitor-therapy refractory patients.[44] Beyond this, current checkpoint inhibitors, e.g., PD1 or PD1L inhibitors may also exert effects on endogenous NK cells and, hence, possibly also enhance the efficacy of adoptively transferred NK cells.[45,46] In the latter context, new generations of more NK cell-specific checkpoint inhibitors are also under development.[47]

As noted above, autologous NK cells readily allow multiple dosing, a strategy that may be particularly challenging in many settings of allogeneic therapies. As deduced from the example of the present clinical study, we reason that autologous NK cell-based therapies may be particularly suited for patients with low tumor burden, e.g., in hematological remission but MRD positive. The present reasoning may also apply to other indications where tumor reduction is achieved by irradiation, surgical resection, and/or chemotherapy. This builds on earlier studies in experimental model systems, demonstrating the capacity of NK cells to preferentially target small tumor grafts.[48]

In the present context of autologous NK cell-based immunotherapies, it is noteworthy that the rapid development and recent advances in the autologous T cell therapy field; e.g., the isolation and usage of tumor-infiltrating lymphocytes (TILs)[49] or chimeric antigen receptor (CAR) T cells[50] have made stunning progress. In this context, despite obvious differences among T cells and NK cells, the overall development of autologous NK cell-based immunotherapies has been significantly lagging behind that of T cells. However, CARs are now making their way into adoptive NK cell-based immunotherapies.[51–53] One feature of NK cells in contrast to T cells is that they, in addition to introduced CARs, may still be able to recognize tumors through their germline-encoded receptors, reducing the potential risk of tumor escape through antigen modulation. Finally, autologous NK cell products could readily be used in combination with other treatment modalities such as monoclonal antibodies,[54,55] bi or tri-specific-engagers,[56,57] proteasome inhibitors,[58] or other types of immunomodulatory drugs.

Figure 3. Assessment of the plasma proteome in conjunction with each NK cell product infusion

(A) A total of 92 plasma proteins were assessed by proximity extension assay. As described in STAR Methods, 8 were excluded from analysis. The heat map shows the log2-based npx values from each of the 6 study subjects translated into fold change related to the value of the pre-infusion sample of each infusion. Red indicates fold increase; blue indicates fold decrease.

(B–E) Assessment of peripheral blood plasma granzyme B (B), granzyme A (C), granzyme H (D), and IL-6 (E) in relation to infusion of *ex vivo* activated and expanded NK cells. The relative concentration was measured by proximity extension assay and is presented in arbitrary log2-based units npx for all study subjects (n = 6).

(F) Assessment of bone marrow plasma granzyme B at diagnosis and after infusion of *ex vivo* activated and expanded NK cells by ELISA. Data shown from 4 study subjects; p = 0.021, paired t test.

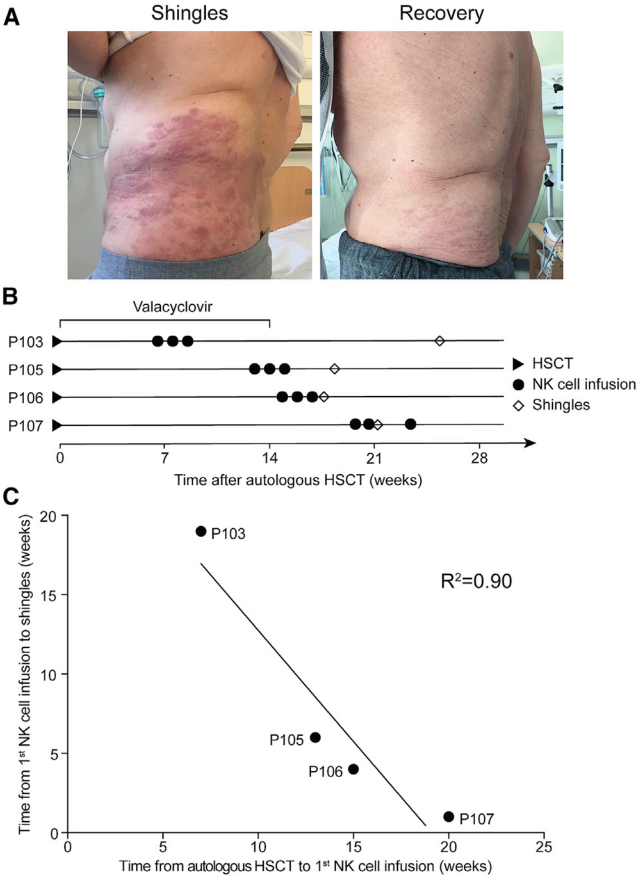

Figure 5. Development of shingles in the first 4 patients following NK cell product infusion

(A) Shingles in 1 study subject following NK cell product infusion (left) and recovery after antiviral treatment (right).

(B) Development of shingles in relation to NK cell product infusion. Diamonds mark the first appearance of shingles. Period of post-HSCT valacyclovir treatment is indicated (n = 4).

(C) Correlation analysis between the time from HSCT to the NK cell product infusion and the time from NK cell product infusion to development of shingles. Regression calculated by Pearson correlation.

In summary, the results presented here open up the possibility for further clinical studies utilizing autologous NK cells, e.g., in settings where patients are not readily eligible for allogeneic NK cell-based products. The latter could include MRD and/or consolidation treatment in human cancer. Provided use of properly activated and expanded cells, whether or not in combinations with other drugs, the present results suggest that use of autologous NK cells merits further investigation in the context of future cancer treatment.

Limitations of the study

Although positive aspects of the study are observed, the present study, focusing on safety and feasibility assessment, is not sufficient to provide a firm conclusion of clinical efficacy of the present treatment. For the latter, a larger study including a control group is needed. Even though it is alluring to compare progression-free and overall survival rates to the same time period cohorts, we believe it might be misleading, considering the small size of the trial. It cannot be excluded that newer maintenance therapies introduced in this patient group may affect the present therapeutic strategy. With respect to the granzyme B data, it cannot be concluded that this is due to the interaction between infused immune effector cells and tumor cells. Likewise, the physiopathology behind shingles manifestation is speculative, based on earlier studies.

Finally, NK cell expansions in closed bioreactor systems are technically challenging and require extensive process optimization. As mentioned above, of 10 expansions, 6 were able to fulfill the release criteria, whereas 1 was directly attributable to a bioreactor-specific consumable error and another was attributable to bioreactor technical error. It is not uncommon to have a higher rate of batch manufacturing failures in early clinical trials. However, the process has currently been optimized, resulting in decreased failure rates.

STAR METHODS

Detailed methods are provided in the online version of this paper and include the following:

- KEY RESOURCES TABLE
- RESOURCE AVAILABILITY
  - Lead contact
  - Materials availability
  - Data and code availability
- EXPERIMENTAL MODEL AND SUBJECT DETAILS
  - Clinical study protocol for autologous NK cell-based immunotherapy of MM
  - Study approval
- METHOD DETAILS
  - Generation of good manufacturing practice (GMP) *ex vivo* activated and expanded NK cells for the clinical study
  - Measurement of M-component and serum free light chain
  - Measurement of minimal residual disease (MRD)
  - Flow cytometry
  - Measurement of cytokines, chemokines, and other proteins
  - Measurement of granzyme B from bone marrow-derived plasma
- QUANTIFICATION AND STATISTICAL ANALYSIS
- ADDITIONAL RESOURCES

SUPPLEMENTAL INFORMATION

Supplemental information can be found online at https://doi.org/10.1016/j.xcrm.2022.100508.

ACKNOWLEDGMENTS

We thank O. Bunte, H. Concha Quezada, S. Dilber, A.D. Duru, E. Hellström-Lindberg, K. LeBlanc, C. Lindström, K.-J. Malmberg, J. Michaëlsson, C. Nilsson, N. Renkvist, E. Watz, L. Wikingsson, and K. Wikström for fruitful discussions and, in applicable cases, technical support. We would like to

CellPress
OPEN ACCESS

acknowledge the Stem Cell Laboratory and Apheresis Unit (Karolinska University Hospital), as well as the MedH Flow Cytometry core facility (Karolinska Institutet), supported by Karolinska Institutet/Region Stockholm.

This study has been funded by the Tobias Foundation (H.-G.L.), the Swedish Cancer Society CAN 2017/505 (H.-G.L.), the Swedish Research Council VR2015-02499 (H.-G.L.), Radiumhemmets Forskningsfonder 184083 (H.-G.L.) and 191063 (E.A.), Sweden's Innovation Agency 2010-00501 (P.B., H.-G.L., and E.A.), Swedish Foundation for Strategic Research RIF14-0045 (P.B.), Region Stockholm 20190762CIMED (H.N., P.B., E.A., and H.-G.L.), and Karolinska Institutet (H.N., E.A., and H.-G.L.).

AUTHOR CONTRIBUTIONS

H.N., clinical study Principal Investigator, clinical care, and interpretation of results; M.C., cell product development and proteomic analyses; S.M., flow cytometry analyses and interpretation of results; C. Gran, clinical chemistry and follow-up; N.M., flow cytometry panel design; G.A., clinical follow-up; T.S., process development; M.G., cell product development; B.S., cell product development; A.K.W., flow cytometry panel design and interpretation of results; P.B., GMP-process design; P.-H.H., cell product development; L.W.-J., cell product stability assays; K.M., clinical study management; J. Liwing, clinical study management and statistical analyses; C. Gustafsson, MRD analysis; R.M., MRD analysis; M.K., histopathological examination and analyses; G.G., clinical advice and guidance; J. Lund, clinical care; P.L., clinical study advisor; H.-G.L., clinical study design and analyses, interpretation of results, and conceptualization of manuscript layout; E.A. conceptualized study design, overall study coordination, and interpretation of results. S.M., H.N., H.-G.L., and E.A. wrote the manuscript. All authors read, commented on, and approved the final version of the manuscript.

DECLARATION OF INTERESTS

J. Liwing and P.-H.H. are employed by XNK Therapeutics (XNK); H.N., S.M., and M.C. are consulting for XNK; H.-G.L., is a board member of XNK; M.G., B.S., L.W.-J., K.M., G.G., H.-G.L., and E.A. are minority shareholders of XNK. A patent application pertaining to the use of antiviral prophylaxis in the context of autologous NK cell infusions has been filed (WO 2019/211310 A1). The remaining authors have declared that no competing interests exist.

Received: March 30, 2021
Revised: November 3, 2021
Accepted: January 5, 2022
Published: January 28, 2022

REFERENCES

1. Miller, J.S., Soignier, Y., Panoskaltsis-Mortari, A., McNearney, S.A., Yun, G.H., Fautsch, S.K., McKenna, D., Le, C., Defor, T.E., Burns, L.J., et al. (2005). Successful adoptive transfer and in vivo expansion of human haploidentical NK cells in patients with cancer. Blood *105*, 3051–3057. https://doi.org/10.1182/blood-2004-07-2974.
2. Bachanova, V., Cooley, S., Defor, T.E., Verneris, M.R., Zhang, B., McKenna, D.H., Curtsinger, J., Panoskaltsis-Mortari, A., Lewis, D., Hippen, K., et al. (2014). Clearance of acute myeloid leukemia by haploidentical natural killer cells is improved using IL-2 diphtheria toxin fusion protein. Blood *123*, 3855–3863. https://doi.org/10.1182/blood-2013-10-532531.
3. Romee, R., Rosario, M., Berrien-Elliott, M.M., Wagner, J.A., Jewell, B.A., Schappe, T., Leong, J.W., Abdel-Latif, S., Schneider, S.E., Willey, S., et al. (2016). Cytokine-induced memory-like natural killer cells exhibit enhanced responses against myeloid leukemia. Sci. Transl Med. *8*, 357ra123. https://doi.org/10.1126/scitranslmed.aaf2341.
4. Ciurea, S.O., Schafer, J.R., Bassett, R., Denman, C.J., Cao, K., Willis, D., Rondon, G., Chen, J., Soebbing, D., Kaur, I., et al. (2017). Phase 1 clinical trial using mbIL21 ex vivo-expanded donor-derived NK cells after haploidentical transplantation. Blood *130*, 1857–1868. https://doi.org/10.1182/blood-2017-05-785659.
5. Bjorklund, A.T., Carlsten, M., Sohlberg, E., Liu, L.L., Clancy, T., Karimi, M., Cooley, S., Miller, J.S., Klimkowska, M., Schaffer, M., et al. (2018). Complete remission with reduction of high-risk clones following haploidentical NK-cell therapy against MDS and AML. Clin. Cancer Res. *24*, 1834–1844. https://doi.org/10.1158/1078-0432.CCR-17-3196.
6. Myers, J.A., and Miller, J.S. (2020). Exploring the NK cell platform for cancer immunotherapy. Nat. Rev. Clin. Oncol. https://doi.org/10.1038/s41571-020-0426-7.
7. Karre, K., Ljunggren, H.G., Piontek, G., and Kiessling, R. (1986). Selective rejection of H-2-deficient lymphoma variants suggests alternative immune defence strategy. Nature *319*, 675–678. https://doi.org/10.1038/319675a0.
8. Ljunggren, H.G., and Karre, K. (1990). In search of the 'missing self': MHC molecules and NK cell recognition. Immunol. Today *11*, 237–244. https://doi.org/10.1016/0167-5699(90)90097-s.
9. Ruggeri, L., Capanni, M., Urbani, E., Perruccio, K., Shlomchik, W.D., Tosti, A., Posati, S., Rogaia, D., Frassoni, F., Aversa, F., et al. (2002). Effectiveness of donor natural killer cell alloreactivity in mismatched hematopoietic transplants. Science *295*, 2097–2100. https://doi.org/10.1126/science.1068440.
10. Ruggeri, L., Mancusi, A., Burchielli, E., Perruccio, K., Aversa, F., Martelli, M.F., and Velardi, A. (2006). Natural killer cell recognition of missing self and haploidentical hematopoietic transplantation. Semin. Cancer Biol. *16*, 404–411. https://doi.org/10.1016/j.semcancer.2006.07.007.
11. Shah, N., Li, L., McCarty, J., Kaur, I., Yvon, E., Shaim, H., Muftuoglu, M., Liu, E., Orlowski, R.Z., Cooper, L., et al. (2017). Phase I study of cord blood-derived natural killer cells combined with autologous stem cell transplantation in multiple myeloma. Br. J. Haematol. *177*, 457–466. https://doi.org/10.1111/bjh.14570.
12. Dolstra, H., Roeven, M.W.H., Spanholtz, J., Hangalapura, B.N., Tordoir, M., Maas, F., Leenders, M., Bohme, F., Kok, N., Trilsbeek, C., et al. (2017). Successful transfer of umbilical cord blood CD34(+) hematopoietic stem and progenitor-derived NK cells in older acute myeloid leukemia patients. Clin. Cancer Res. *23*, 4107–4118. https://doi.org/10.1158/1078-0432.CCR-16-2981.
13. Zhu, H., Blum, R.H., Bjordahl, R., Gaidarova, S., Rogers, P., Lee, T.T., Abujarour, R., Bonello, G.B., Wu, J., Tsai, P.F., et al. (2020). Pluripotent stem cell-derived NK cells with high-affinity noncleavable CD16a mediate improved antitumor activity. Blood *135*, 399–410. https://doi.org/10.1182/blood.2019000621.
14. Mitwasi, N., Feldmann, A., Arndt, C., Koristka, S., Berndt, N., Jureczek, J., Loureiro, L.R., Bergmann, R., Mathe, D., Hegedus, N., et al. (2020). "UniCAR"-modified off-the-shelf NK-92 cells for targeting of GD2-expressing tumour cells. Sci. Rep. *10*, 2141. https://doi.org/10.1038/s41598-020-59082-4.
15. Shimasaki, N., Jain, A., and Campana, D. (2020). NK cells for cancer immunotherapy. Nat. Rev. Drug Discov. *19*, 200–218. https://doi.org/10.1038/s41573-019-0052-1.
16. Tschan-Plessl, A., Kalberer, C.P., Wieboldt, R., Stern, M., Siegler, U., Wodnar-Filipowicz, A., Gerull, S., Halter, J., Heim, D., Tichelli, A., et al. (2021). Cellular immunotherapy with multiple infusions of in vitro-expanded haploidentical natural killer cells after autologous transplantation for patients with plasma cell myeloma. Cytotherapy *23*, 329–338. https://doi.org/10.1016/j.jcyt.2020.09.009.
17. Rosenberg, S.A., Lotze, M.T., Muul, L.M., Leitman, S., Chang, A.E., Ettinghausen, S.E., Matory, Y.L., Skibber, J.M., Shiloni, E., Vetto, J.T., et al. (1985). Observations on the systemic administration of autologous lymphokine-activated killer cells and recombinant interleukin-2 to patients with metastatic cancer. N. Engl. J. Med. *313*, 1485–1492. https://doi.org/10.1056/NEJM198512053132327.
18. Burns, L.J., Weisdorf, D.J., DeFor, T.E., Vesole, D.H., Repka, T.L., Blazar, B.R., Burger, S.R., Panoskaltsis-Mortari, A., Keever-Taylor, C.A., Zhang,

M.J., and Miller, J.S. (2003). IL-2-based immunotherapy after autologous transplantation for lymphoma and breast cancer induces immune activation and cytokine release: a phase I/II trial. Bone Marrow Transpl. *32*, 177–186. https://doi.org/10.1038/sj.bmt.1704086.

19. Parkhurst, M.R., Riley, J.P., Dudley, M.E., and Rosenberg, S.A. (2011). Adoptive transfer of autologous natural killer cells leads to high levels of circulating natural killer cells but does not mediate tumor regression. Clin. Cancer Res. *17*, 6287–6297. https://doi.org/10.1158/1078-0432.CCR-11-1347.
20. Alici, E., Sutlu, T., Bjorkstrand, B., Gilljam, M., Stellan, B., Nahi, H., Quezada, H.C., Gahrton, G., Ljunggren, H.G., and Dilber, M.S. (2008). Autologous antitumor activity by NK cells expanded from myeloma patients using GMP-compliant components. Blood *111*, 3155–3162. https://doi.org/10.1182/blood-2007-09-110312.
21. Guimaraes, F., Guven, H., Donati, D., Christensson, B., Ljunggren, H.G., Bejarano, M.T., and Dilber, M.S. (2006). Evaluation of ex vivo expanded human NK cells on antileukemia activity in SCID-beige mice. Leukemia *20*, 833–839. https://doi.org/10.1038/sj.leu.2404147.
22. Alici, E., Konstantinidis, K.V., Sutlu, T., Aints, A., Gahrton, G., Ljunggren, H.G., and Dilber, M.S. (2007). Anti-myeloma activity of endogenous and adoptively transferred activated natural killer cells in experimental multiple myeloma model. Exp. Hematol. *35*, 1839–1846. https://doi.org/10.1016/j.exphem.2007.08.006.
23. Garg, T.K., Szmania, S.M., Khan, J.A., Hoering, A., Malbrough, P.A., Moreno-Bost, A., Greenway, A.D., Lingo, J.D., Li, X., Yaccoby, S., et al. (2012). Highly activated and expanded natural killer cells for multiple myeloma immunotherapy. Haematologica *97*, 1348–1356. https://doi.org/10.3324/haematol.2011.056747.
24. Sutlu, T., Stellan, B., Gilljam, M., Quezada, H.C., Nahi, H., Gahrton, G., and Alici, E. (2010). Clinical-grade, large-scale, feeder-free expansion of highly active human natural killer cells for adoptive immunotherapy using an automated bioreactor. Cytotherapy *12*, 1044–1055. https://doi.org/10.3109/14653249.2010.504770.
25. Rajkumar, S.V., Harousseau, J.L., Durie, B., Anderson, K.C., Dimopoulos, M., Kyle, R., Blade, J., Richardson, P., Orlowski, R., Siegel, D., et al. (2011). Consensus recommendations for the uniform reporting of clinical trials: report of the International Myeloma Workshop Consensus Panel 1. Blood *117*, 4691–4695. https://doi.org/10.1182/blood-2010-10-299487.
26. Oken, M.M., Creech, R.H., Tormey, D.C., Horton, J., Davis, T.E., McFadden, E.T., and Carbone, P.P. (1982). Toxicity and response criteria of the eastern cooperative Oncology group. Am. J. Clin. Oncol. *5*, 649–655.
27. Greipp, P.R., San Miguel, J., Durie, B.G., Crowley, J.J., Barlogie, B., Blade, J., Boccadoro, M., Child, J.A., Avet-Loiseau, H., Kyle, R.A., et al. (2005). International staging system for multiple myeloma. J. Clin. Oncol. *23*, 3412–3420. https://doi.org/10.1200/JCO.2005.04.242.
28. Palumbo, A., Avet-Loiseau, H., Oliva, S., Lokhorst, H.M., Goldschmidt, H., Rosinol, L., Richardson, P., Caltagirone, S., Lahuerta, J.J., Facon, T., et al. (2015). Revised international staging system for multiple myeloma: a report from international myeloma working group. J. Clin. Oncol. *33*, 2863–2869. https://doi.org/10.1200/JCO.2015.61.2267.
29. Chng, W.J., Dispenzieri, A., Chim, C.S., Fonseca, R., Goldschmidt, H., Lentzsch, S., Munshi, N., Palumbo, A., Miguel, J.S., Sonneveld, P., et al. (2014). IMWG consensus on risk stratification in multiple myeloma. Leukemia *28*, 269–277. https://doi.org/10.1038/leu.2013.247.
30. Palumbo, A., Cavallo, F., Gay, F., Di Raimondo, F., Ben Yehuda, D., Petrucci, M.T., Pezzatti, S., Caravita, T., Cerrato, C., Ribakovsky, E., et al. (2014). Autologous transplantation and maintenance therapy in multiple myeloma. N. Engl. J. Med. *371*, 895–905. https://doi.org/10.1056/NEJMoa1402888.
31. Al Hamed, R., Bazarbachi, A.H., Malard, F., Harousseau, J.L., and Mohty, M. (2019). Current status of autologous stem cell transplantation for multiple myeloma. Blood Cancer J. *9*, 44. https://doi.org/10.1038/s41408-019-0205-9.
32. Kamber, C., Zimmerli, S., Suter-Riniker, F., Mueller, B.U., Taleghani, B.M., Betticher, D., Zander, T., and Pabst, T. (2015). Varicella zoster virus reactivation after autologous SCT is a frequent event and associated with favorable outcome in myeloma patients. Bone Marrow Transpl. *50*, 573–578. https://doi.org/10.1038/bmt.2014.290.
33. Backstrom, E., Chambers, B.J., Kristensson, K., and Ljunggren, H.G. (2000). Direct NK cell-mediated lysis of syngenic dorsal root ganglia neurons in vitro. J. Immunol. *165*, 4895–4900. https://doi.org/10.4049/jimmunol.165.9.4895.
34. Backstrom, E., Chambers, B.J., Ho, E.L., Naidenko, O.V., Mariotti, R., Fremont, D.H., Yokoyama, W.M., Kristensson, K., and Ljunggren, H.G. (2003). Natural killer cell-mediated lysis of dorsal root ganglia neurons via RAE1/NKG2D interactions. Eur. J. Immunol. *33*, 92–100. https://doi.org/10.1002/immu.200390012.
35. Davies, A.J., Kim, H.W., Gonzalez-Cano, R., Choi, J., Back, S.K., Roh, S.E., Johnson, E., Gabriac, M., Kim, M.S., Lee, J., et al. (2019). Natural killer cells degenerate intact sensory afferents following nerve injury. Cell *176*, 716–728 e8. https://doi.org/10.1016/j.cell.2018.12.022.
36. Guven, H., Gilljam, M., Chambers, B.J., Ljunggren, H.G., Christensson, B., Kimby, E., and Dilber, M.S. (2003). Expansion of natural killer (NK) and natural killer-like T (NKT)-cell populations derived from patients with B-chronic lymphocytic leukemia (B-CLL): a potential source for cellular immunotherapy. Leukemia *17*, 1973–1980. https://doi.org/10.1038/sj.leu.2403083.
37. Anfossi, N., Andre, P., Guia, S., Falk, C.S., Roetynck, S., Stewart, C.A., Breso, V., Frassati, C., Reviron, D., Middleton, D., et al. (2006). Human NK cell education by inhibitory receptors for MHC class I. Immunity *25*, 331–342. https://doi.org/10.1016/j.immuni.2006.06.013.
38. Guia, S., Jaeger, B.N., Piatek, S., Mailfert, S., Trombik, T., Fenis, A., Chevrier, N., Walzer, T., Kerdiles, Y.M., Marguet, D., et al. (2011). Confinement of activating receptors at the plasma membrane controls natural killer cell tolerance. Sci. Signal. *4*, ra21. https://doi.org/10.1126/scisignal.2001608.
39. Goodridge, J.P., Jacobs, B., Saetersmoen, M.L., Clement, D., Hammer, Q., Clancy, T., Skarpen, E., Brech, A., Landskron, J., Grimm, C., et al. (2019). Remodeling of secretory lysosomes during education tunes functional potential in NK cells. Nat. Commun. *10*, 514. https://doi.org/10.1038/s41467-019-08384-x.
40. Liu, L.L., Beziat, V., Oei, V.Y.S., Pfefferle, A., Schaffer, M., Lehmann, S., Hellstrom-Lindberg, E., Soderhall, S., Heyman, M., Grander, D., and Malmberg, K.J. (2017). Ex vivo expanded adaptive NK cells effectively kill primary acute lymphoblastic leukemia cells. Cancer Immunol. Res. *5*, 654–665. https://doi.org/10.1158/2326-6066.CIR-16-0296.
41. Klippel, Z.K., Chou, J., Towlerton, A.M., Voong, L.N., Robbins, P., Bensinger, W.I., and Warren, E.H. (2014). Immune escape from NY-ESO-1-specific T-cell therapy via loss of heterozygosity in the MHC. Gene Ther. *21*, 337–342. https://doi.org/10.1038/gt.2013.87.
42. Garrido, F., Ruiz-Cabello, F., and Aptsiauri, N. (2017). Rejection versus escape: the tumor MHC dilemma. Cancer Immunol. Immunother. *66*, 259–271. https://doi.org/10.1007/s00262-016-1947-x.
43. Zaretsky, J.M., Garcia-Diaz, A., Shin, D.S., Escuin-Ordinas, H., Hugo, W., Hu-Lieskovan, S., Torrejon, D.Y., Abril-Rodriguez, G., Sandoval, S., Barthly, L., et al. (2016). Mutations associated with acquired resistance to PD-1 blockade in melanoma. N. Engl. J. Med. *375*, 819–829. https://doi.org/10.1056/NEJMoa1604958.
44. Malmberg, K.J., Sohlberg, E., Goodridge, J.P., and Ljunggren, H.G. (2017). Immune selection during tumor checkpoint inhibition therapy paves way for NK-cell "missing self" recognition. Immunogenetics *69*, 547–556. https://doi.org/10.1007/s00251-017-1011-9.
45. Neo, S.Y., Yang, Y., Record, J., Ma, R., Chen, X., Chen, Z., Tobin, N.P., Blake, E., Seitz, C., Thomas, R., et al. (2020). CD73 immune checkpoint defines regulatory NK cells within the tumor microenvironment. J. Clin. Invest. *130*, 1185–1198. https://doi.org/10.1172/JCI128895.
46. Hsu, J., Hodgins, J.J., Marathe, M., Nicolai, C.J., Bourgeois-Daigneault, M.C., Trevino, T.N., Azimi, C.S., Scheer, A.K., Randolph, H.E., Thompson,

CellPress
OPEN ACCESS

T.W., et al. (2018). Contribution of NK cells to immunotherapy mediated by PD-1/PD-L1 blockade. J. Clin. Invest. *128*, 4654–4668. https://doi.org/10.1172/JCI99317.

47. Andre, P., Denis, C., Soulas, C., Bourbon-Caillet, C., Lopez, J., Arnoux, T., Blery, M., Bonnafous, C., Gauthier, L., Morel, A., et al. (2018). Anti-NKG2A mAb is a checkpoint inhibitor that promotes anti-tumor immunity by unleashing both T and NK cells. Cell *175*, 1731–1743.e3. https://doi.org/10.1016/j.cell.2018.10.014.

48. Ljunggren, H.G., and Karre, K. (1985). Host resistance directed selectively against H-2-deficient lymphoma variants. Analysis of the mechanism. J. Exp. Med. *162*, 1745–1759. https://doi.org/10.1084/jem.162.6.1745.

49. Mullinax, J.E., Hall, M., Prabhakaran, S., Weber, J., Khushalani, N., Eroglu, Z., Brohl, A.S., Markowitz, J., Royster, E., Richards, A., et al. (2018). Combination of ipilimumab and adoptive cell therapy with tumor-infiltrating lymphocytes for patients with metastatic melanoma. Front. Oncol. *8*, 44. https://doi.org/10.3389/fonc.2018.00044.

50. Fraietta, J.A., Lacey, S.F., Orlando, E.J., Pruteanu-Malinici, I., Gohil, M., Lundh, S., Boesteanu, A.C., Wang, Y., O'Connor, R.S., Hwang, W.T., et al. (2018). Determinants of response and resistance to CD19 chimeric antigen receptor (CAR) T cell therapy of chronic lymphocytic leukemia. Nat. Med. *24*, 563–571. https://doi.org/10.1038/s41591-018-0010-1.

51. Liu, E., Tong, Y., Dotti, G., Shaim, H., Savoldo, B., Mukherjee, M., Orange, J., Wan, X., Lu, X., Reynolds, A., et al. (2018). Cord blood NK cells engineered to express IL-15 and a CD19-targeted CAR show long-term persistence and potent antitumor activity. Leukemia *32*, 520–531. https://doi.org/10.1038/leu.2017.226.

52. Liu, E., Marin, D., Banerjee, P., Macapinlac, H.A., Thompson, P., Basar, R., Nassif Kerbauy, L., Overman, B., Thall, P., Kaplan, M., et al. (2020). Use of CAR-transduced natural killer cells in CD19-positive lymphoid tumors. N. Engl. J. Med. *382*, 545–553. https://doi.org/10.1056/NEJMoa1910607.

53. Leivas, A., Valeri, A., Cordoba, L., Garcia-Ortiz, A., Ortiz, A., Sanchez-Vega, L., Grana-Castro, O., Fernandez, L., Carreno-Tarragona, G., Perez, M., et al. (2021). NKG2D-CAR-transduced natural killer cells efficiently target multiple myeloma. Blood Cancer J. *11*, 146. https://doi.org/10.1038/s41408-021-00537-w.

54. Usmani, S.Z., Weiss, B.M., Plesner, T., Bahlis, N.J., Belch, A., Lonial, S., Lokhorst, H.M., Voorhees, P.M., Richardson, P.G., Chari, A., et al. (2016). Clinical efficacy of daratumumab monotherapy in patients with heavily pretreated relapsed or refractory multiple myeloma. Blood *128*, 37–44. https://doi.org/10.1182/blood-2016-03-705210.

55. Lee, S.C., Shimasaki, N., Lim, J.S.J., Wong, A., Yadav, K., Yong, W.P., Tan, L.K., Koh, L.P., Poon, M.L.M., Tan, S.H., et al. (2020). Phase I trial of expanded, activated autologous NK-cell infusions with trastuzumab in patients with HER2-positive cancers. Clin. Cancer Res. https://doi.org/10.1158/1078-0432.CCR-20-0768.

56. Davis, Z.B., Vallera, D.A., Miller, J.S., and Felices, M. (2017). Natural killer cells unleashed: checkpoint receptor blockade and BiKE/TriKE utilization in NK-mediated anti-tumor immunotherapy. Semin. Immunol. *31*, 64–75. https://doi.org/10.1016/j.smim.2017.07.011.

57. Ellwanger, K., Reusch, U., Fucek, I., Wingert, S., Ross, T., Muller, T., Schniegler-Mattox, U., Haneke, T., Rajkovic, E., Koch, J., et al. (2019). Redirected optimized cell killing (ROCK(R)): a highly versatile multispecific fit-for-purpose antibody platform for engaging innate immunity. MAbs *11*, 899–918. https://doi.org/10.1080/19420862.2019.1616506.

58. Lundqvist, A., Berg, M., Smith, A., and Childs, R.W. (2011). Bortezomib treatment to potentiate the anti-tumor immunity of ex-vivo expanded adoptively infused autologous natural killer cells. J. Cancer *2*, 383–385. https://doi.org/10.7150/jca.2.383.

STAR METHODS

KEY RESOURCES TABLE

REAGENT or RESOURCE	SOURCE	IDENTIFIER
Antibodies		
Mouse monoclonal anti-CD56 BUV563 (clone NCAM16.2)	BD BioSciences	Cat# 565704, Lot# 8250989; RRID: AB_2744431
Mouse monoclonal anti-CD16 BUV496 (clone 3G8)	BD BioSciences	Cat# 564653, lot# 8291900; RRID: AB_2744294
Mouse monoclonal anti-NKp44 AlexaFluor647 (clone p44-8)	BD BioSciences	Cat# 558564, lot# 5082973; RRID: AB_647153
Mouse monoclonal anti-CD25 PE-CF594 (clone M-A251)	BD BioSciences	Cat# 562403, lot# 9099748; RRID: AB_11151919
Mouse monoclonal anti-PD1 BB700 (clone EH12.1)	BD BioSciences	Cat# 566460, Lot# 9182916, 8346689; RRID: AB_2744348
Mouse monoclonal anti-HLA-DR BV650 (clone G46-6)	BD BioSciences	Cat# 564231, lot# 9017957; RRID: AB_2738685
Mouse monoclonal anti-TIM-3 BV711 (clone 7D3)	BD BioSciences	Cat# 565566, lot# 9049619, 9116947; RRID: AB_2744370
Mouse monoclonal anti-CD38 BV738 (clone HIT2)	BD BioSciences	Cat# 563964, lot# 7338505, 9151925; RRID: AB_2738515
Mouse monoclonal anti-DNAM-1 BUV395 (clone DX11)	BD BioSciences	Cat# 742498, lot# 9136597, 9276805, 9276799, 9276800; RRID: AB_2740831
Mouse monoclonal anti-CXCR4 BUV737 (clone 12G5)	BD BioSciences	Cat# 741862, lot# 9276807, 9276809, 9276810; RRID: AB_2871192
Mouse monoclonal anti-Ki67 AlexaFluor700 (clone B56)	BD BioSciences	Cat# 561277, lot# 7349946; RRID: AB_10611571
Mouse monoclonal anti-perforin 1 BB755-P (clone δG9)	BD BioSciences	Custom conjugate, Cat# 624391, lot# 9101891
Mouse monoclonal anti-granzyme B BB790-P (clone GB11)	BD BioSciences	Custom conjugate, Cat# 624296, lot# 9130980
Mouse monoclonal anti-CD19 V500 (clone HIB19)	BD BioSciences	Cat# 561121, lot# 7066974; RRID: AB_10562391
Mouse monoclonal anti-CD14 V500 (clone MΦP9)	BD BioSciences	Cat# 562693, lot# 8215846; RRID: AB_2737727
Mouse monoclonal anti-CD3 PE-Cy5 (clone HIT3a)	BioLegend	Cat# 300310, lot# B181404, B291468; RRID: AB_314046
Mouse monoclonal anti-NKG2D BV421 (clone 1D11)	BioLegend	Cat# 320821, lot# B274633; RRID: AB_2566510
Mouse monoclonal anti-2B4 APC-Cy7 (clone C1.7)	BioLegend	Cat# 329518, lot# B329518, B219470, B276619; RRID: AB_2572015
Mouse monoclonal anti-CD319 PE (clone 162.1)	BioLegend	Cat# 331806, lot# B268818; RRID: AB_2239190
Mouse monoclonal anti-LAG-3 PE-Cy7 (11C3C65)	BioLegend	Cat# 369310, lot# B289010; RRID: AB_2629753
Mouse monoclonal anti-TIGIT BV605 (clone A15153G)	BioLegend	Cat# 372711, lot# B240084, B281575; RRID: AB_2632926
Mouse monoclonal anti-NKG2A VioBright FITC (clone REA110)	Miltenyi Biotec	Cat# 130-105-646, lot# 5160809205; RRID: AB_2655382
Orthoclone OKT3 (MACS GMP CD3 pure)	Miltenyi Biotech	Cat# 170-076-124
Biological samples		
Human AB serum	Lonza	Cat# 14-490E; current Cat# 4W-320
Chemicals, peptides, and recombinant proteins		
CellGro SCGM serum-free cell culture media	CellGenix	Cat# 0020902-0500
IL-2 (Proleukin)	Chiron, currently Clinigen	https://www.medicines.org.uk/emc/product/291/smpc
Pluronic F68	Life Technologies	Cat# 24040032

(Continued on next page)

Continued

REAGENT or RESOURCE	SOURCE	IDENTIFIER
Critical commercial assays		
eBioscience Foxp3/Transcription Factor Staining Buffer Set	Thermo Fisher Scientific	Cat# 00-5523-00
LIVE/DEAD Fixable Aqua Dead Cell Stain Kit	Thermo Fisher Scientific	Cat# L34957
LymphoTrack Dx IGH FR1/FR2/ FR3 Assay Kit A -MiSeq	Invivoscribe	Cat# 9-121-0129
QIAamp DNA micro kit	Qiagen	Cat# 56304
QIAamp DNA Blood Midi Kit	Qiagen	Cat# 51183
Agencourt AMPure XP beads	Beckman Coulter	Cat# A63880
Qubit dsDNA HS Assay Kit	Invitrogen	Cat# Q32854
Agilent High Sensitivity DNA ScreenTape Assay	Agilent	Cat# 5067-5584
Olink Immuno-Oncology panel v.3101	Olink Bioscience	Cat# 95310
PeliKine Compact human GRANZYME B ELISA Kit	Sanquin CLB	Ref# M1936, no longer available
Software and algorithms		
FlowJo v.10	Treestar inc.	RRID: SCR_008520
GraphPad Prism v.8	GraphPad Software	RRID: SCR_002798
LymphoTrack Dx Software – MiSeq Version 2.4.3	Invivoscribe	Cat# 95000009
LymphoTrack MRD Software v1.2.0	Invivoscribe	Cat# 75000008
Other		
WAVE Bioreactor^{TM} System 2/10	GE Healthcare	
Cobas 8000 system	Roche Diagnostics	https://diagnostics.roche.com/global/en/products/systems/cobas_-8000-modular-analyzer-series.html#productInfo
Beckman Coulter IMMAGE 800 Protein Chemistry Analyzer	Beckman Coulter	RRID: SCR_019642
BN ProSpec	Siemens Healthcare GmbH	https://www.siemens-healthineers.com/se/plasma-protein/systems/bn-prospec-system
Atellica NEPH 630 system	Siemens Healthcare GmbH	https://www.siemens-healthineers.com/se/plasma-protein/systems/atellica-neph-630-system
Sebia Hydrasys LC system	Sébia	https://www.sebia.com/instruments/hydrasys-2-scan-focusing/

RESOURCE AVAILABILITY

Lead contact

Further information and requests for resources and reagents should be directed to and will be fulfilled by the lead contact, Evren Alici (evren.alici@ki.se).

Materials availability

This study did not generate new unique reagents.

Data and code availability

- Study protocol-related data have been submitted to European Union Drug Regulating Authorities Clinical Trials Database (EudraCT). Anonymous data and other information will be made available upon request to the corresponding author following publication of the present article. Data will be made available in a form not deviating from what is accepted by local regulatory authorities with respect to handling of patient data, and in adherence of the policies of the Karolinska University Hospital and Karolinska Institutet.
- This study does not generate any custom code.
- Any additional information required to reanalyze the data reported in this work paper is available from the Lead Contact upon request.

EXPERIMENTAL MODEL AND SUBJECT DETAILS

Clinical study protocol for autologous NK cell-based immunotherapy of MM

The synopsis of the study protocol is presented in Methods S1. Informed consent to inclusion in the clinical study was obtained at diagnosis. Eleven patients referred to the Hematological Center, Karolinska University Hospital, were enrolled in the study. One patient withdrew consent for participation prior to blood donation for production of the NK cell product. From ten MM patients peripheral blood was collected and NK cells were activated and expanded *ex vivo* as described above. NK cell products from six patients met the release criteria. These patients were infused with the investigational NK cell-based product and were followed in the study (P103, P105, P106, P107, P110, P111). The patient characteristics at inclusion in the study including Ig heavy chain, serum free light chain, Eastern Cooperative Oncology Group (ECOG) scale of performance status,[26] and international staging system (ISS) classification for MM[27,28] are summarized in Table 1. Additional clinical chemistry data on study subjects is provided in Table S1. Briefly, following upfront induction with three to four cycles of cyclophosphamide, bortezomib, and dexamethasone (CyBorD), patients underwent autologous HSCT. Conditioning for HSCT was melphalan 200 mg/m^2 single day. Following autologous HSCT, standard antiviral prophylaxis (valacyclovir) was administered orally twice daily during the first 100 days for the first four study subjects and for six months after the first NK cell product infusion for the fifth and sixth study subject. The study subjects received NK cell infusions when they were in either subclinical relapse after either complete remission (CR), stable partial remission (PR), or PR with asymptomatic progression. The study subjects that had not relapsed or progressed still received NK cell infusions at six months. Study subjects received three escalating doses of 5×10^6 (dose 1), 5×10^7 (dose 2) and up to 1×10^8 (dose 3) NK cell product/kg at weekly intervals (when not noted otherwise). Study subjects were then evaluated for six months after the last infusion. The patients were thereafter continuously followed clinically for up to five years. Of note, the clinical study protocol allowed treatment of twelve patients (Methods S1). However, upon consultation with the Swedish Medical Products Agency during the interim analysis of the clinical study, the cell therapy product with the doses infused was deemed to fulfill the requirements (primary endpoints) outlined in the study protocol. Consequently, the clinical trial was concluded after the interim analysis.

Study approval

The clinical study was approved by the Swedish Medical Products Agency (151:2010/63508; amendment: 5.1-2013-77703) and the Stockholm Regional Ethical Review Board (2010/1618-31/4; amendments: 2013/490-32 and 2018/1899-32). Written informed consent was obtained from the study subjects before inclusion in the study. Studies outside the clinical protocol were approved by the Stockholm Regional Ethical Review Board (2008/1166-31).

METHOD DETAILS

Generation of good manufacturing practice (GMP) *ex vivo* activated and expanded NK cells for the clinical study

The NK cell product used was produced under GMP conditions according to a previously described procedure.[24] Briefly, peripheral blood lymphocytes were separated from 450 mL blood, donated by the patient at diagnosis. The lymphocytes were expanded for 20 days using a closed Wave bioreactor system (WAVE Bioreactor^{TM} System 2/10, GE Healthcare). The cells were grown in a disposable cell bag, containing CellGro SCGM serum-free cell culture media (CellGenix), supplemented with 500 IU/mL IL-2 (Proleukin, Chiron), 10 ng/mL orthoclone OKT3 (Miltenyi Biotech), 5% (v/v) human AB serum (Lonza) and 0.1% (v/v) pluronic F68 (Life Technologies). The expanded cell product had a composition and phenotype similar to what was previously reported.[24] The final product was frozen in human AB plasma with 5% DMSO and stored at -180 °C until use. The product showed stability of at least ten years in -180 °C (Table S5; data from one validation study shown).

Measurement of M-component and serum free light chain

Quantification of plasma immunoglobulins (M-component) was performed using an immunoturbidimetric method on a Cobas 8000 system (Roche Diagnostics) or an Immage platform (Beckman Coulter). Light chains in urine and serum were quantified using a nephelometric method on a BN ProSpec or Atellica NEPH 630 system (Siemens Healthcare GmbH). Additional quantification of M-component in plasma was performed with gel-electrophoresis on a Sebia Hydrasys LC system (Sébia).

Measurement of minimal residual disease (MRD)

To identify the immunoglobulin heavy chain (IGH) V(D)J sequence of the dominating MM clone(s) at diagnosis, DNA was prepared from dried bone marrow smears on glass slides (prepared at diagnosis. In brief, smears were pre-wetted with drops of Buffer ATL and transferred into a 1.5 mL microcentrifuge tube. Buffer ATL was added to a total of 180 μL and DNA extracted using QIAamp DNA micro kit (Qiagen, cat# 56304) following the manufacturer's instructions for dried blood spots. The LymphoTrack Dx *IGH* FR1/FR2/FR3 Assay Kit A -MiSeq (Invivoscribe, cat# 9-121-0129) was used to identify and quantify *IGH* gene rearrangements according to manufacturer's instructions. In brief, IGH VDJ sequences were amplified using the supplied framework region (FR) 1, −2, and −3 primers using 50–100 ng of DNA per reaction. Amplified DNA was purified using Agencourt AMPure XP beads (Beckman Coulter cat#A63880). Samples were quantified with Qubit dsDNA HS Assay Kit (Invitrogen cat#Q32854) and size determined with Agilent High Sensitivity DNA ScreenTape Assay (Agilent cat#5067–5584). Successfully amplified FR libraries were pooled and

paired-end sequenced (300 × 300 or 250 × 250 cycles) using the Illumina MiSeq system. The IGH V(D)J sequence of the dominating MM clone(s) at diagnosis was subsequently identified using LymphoTrack Dx Software – MiSeq Version 2.4.3 (Invivoscribe), according to the manufacturer's instructions. To subsequently perform MRD analysis on pre- and post-infusion samples, DNA was prepared using the QIAamp DNA Blood Midi Kit (Qiagen cat#51183). In brief, vital frozen unfractionated BM cells were pelleted and resuspended in 1 mL of PBS before proceeding following the manufacturer's instructions. IGH VDJ libraries were prepared as described above (with the FR primers successfully used to determine the IGH VDJ rearrangement in diagnosis samples) using four times 2 μg of DNA per sample. Libraries were paired-end sequenced (250 × 250 cycles), and the IGH V(D)J repertoire determined using the LymphoTrackDx software. MRD detection levels and confidence of MRD negativity was subsequently determined (based on the quantity of DNA utilized for library preparation and sequencing depth) using LymphoTrack MRD Software v1.2.0 (Invivoscribe) according to manufacturer's instructions.

Flow cytometry

Frozen peripheral blood mononuclear cell samples where thawed, counted (Nucleocounter NC-3000, ChemoMetec), and stained with LIVE/DEAD Fixable Aqua Dead Cell Stain (Invitrogen) diluted 1:1000 in PBS for 20 min at 4 °C. Staining for surface markers was performed in PBS with 2% fetal bovine serum and 1 mM EDTA using optimized antibody concentrations (20 min at 4 °C). Antibodies for surface staining were prepared in BD Brilliant Stain Buffer (BD Biosciences). After surface staining, cells were fixed and permeabilized using the Foxp3/transcription factor staining buffer set (eBioscience) and were stained with optimized concentrations of antibodies against Ki67, perforin, and granzyme B (30 min at RT). The samples were analyzed on a BD Symphony A5 (BD Biosciences). CS&T research beads (BD Biosciences) were used to ensure stable performance between experiments. Data analysis was performed using FlowJo v10 (Treestar Inc.). Live NK cells were defined as $CD56^{+}CD3^{-}CD19^{-}CD14^{-}$. For each study subject, the NK cell populations from every time point were concatenated into one data set for clustering analysis. t-SNE was performed on the pooled NK cell data for each study subject using the standard settings (1000 iterations, perplexity 30, Barnes-Hutt algorithm). All markers were included in the analysis except for CD3, CD14, CD19, and the dead cell marker. Clusters emerging after the infusions were identified by overlaying t-SNE plots of the data from before and after infusions.

Measurement of cytokines, chemokines, and other proteins

92 protein biomarkers were analysed by proximity extension assay using the Olink Immuno-Oncology panel (v.3101) at Olink Bioscience, Uppsala, Sweden. Briefly, the proteins were detected using antibody pairs labeled with specific oligonucleotides that hybridize when in close proximity. Their DNA barcodes are subsequently quantified by real-time PCR. Protein levels are reported in arbitrary units, normalized protein expression (npx). Eight analyses were excluded because the proteins were detected in less than 25% of the samples.

Measurement of granzyme B from bone marrow-derived plasma

Granzyme B levels were determined with commercially available ELISA (PeliKine Compact human GrB-ELISA; Sanquin CLB).

QUANTIFICATION AND STATISTICAL ANALYSIS

Statistical analyses were performed using Prism 8 (GraphPad Software). Granzyme B levels in BM plasma were compared using paired t-test. p-values < 0.05 were considered statistically significant. Correlation between the time from HSCT to the NK cell product infusion and the time from NK cell product infusion to the development of shingles was calculated using Pearson correlation.

ADDITIONAL RESOURCES

The study was prospectively registered in the EudraCT database (2010-022330-83) and retrospectively registered at clinicaltrials.gov (NCT04558853).

Cell Reports Medicine, Volume *3*

Supplemental information

Autologous NK cells as consolidation therapy following stem cell transplantation in multiple myeloma

Hareth Nahi, Michael Chrobok, Stephan Meinke, Charlotte Gran, Nicole Marquardt, Gabriel Afram, Tolga Sutlu, Mari Gilljam, Birgitta Stellan, Arnika K. Wagner, Pontus Blomberg, Per-Henrik Holmqvist, Lilian Walther-Jallow, Karin Mellström, Johan Liwing, Charlotte Gustafsson, Robert Månsson, Monika Klimkowska, Gösta Gahrton, Johan Lund, Per Ljungman, Hans-Gustaf Ljunggren, and Evren Alici

Supplemental Material to Nahi *et al*. "Autologous NK Cells as Consolidation Therapy Following Stem Cell Transplantation in Multiple Myeloma"

Supplemental Figures

Figure S1

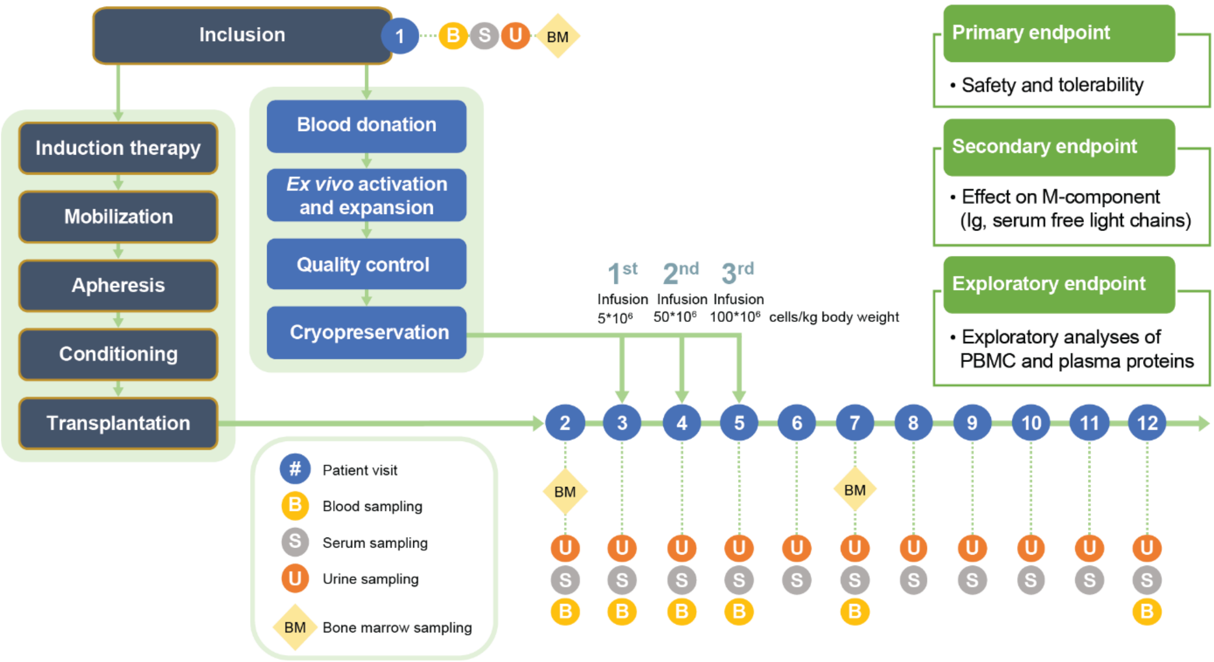

Figure S1. Overview of the clinical study setup. Related to STAR method '*Clinical study protocol for autologous NK cell-based immunotherapy of MM*'. Six study subjects received three escalating doses of 5 x 10^6 (dose 1), 5 x 10^7 (dose 2) and up to 1 x 10^8 (dose 3) NK cell product/kg at weekly intervals. Study subjects were then evaluated for six months following the last infusion. The patients were thereafter continuously followed clinically for up to five years.

Figure S2

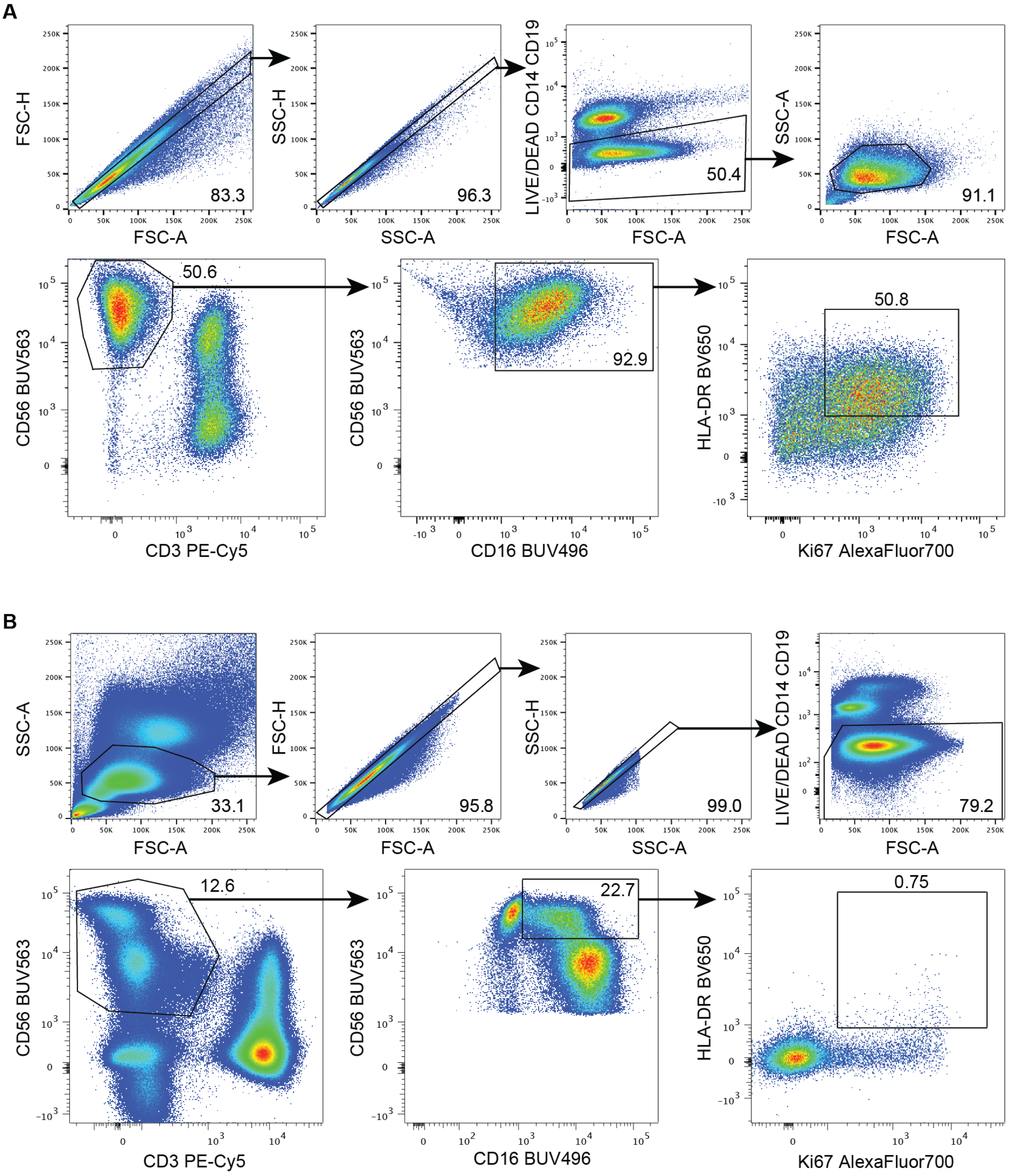

Figure S2. Gating strategy employed to characterize CD56^{bright}CD16^{+}Ki67^{+}HLA-DR^{+} NK cells. Related to Figure 1. Representative plots from one study subject (P107) are shown. **(A)** NK cell product before infusion. **(B)** Study subject PBMC before the first infusion of the NK cell product (same day).

Figure S3

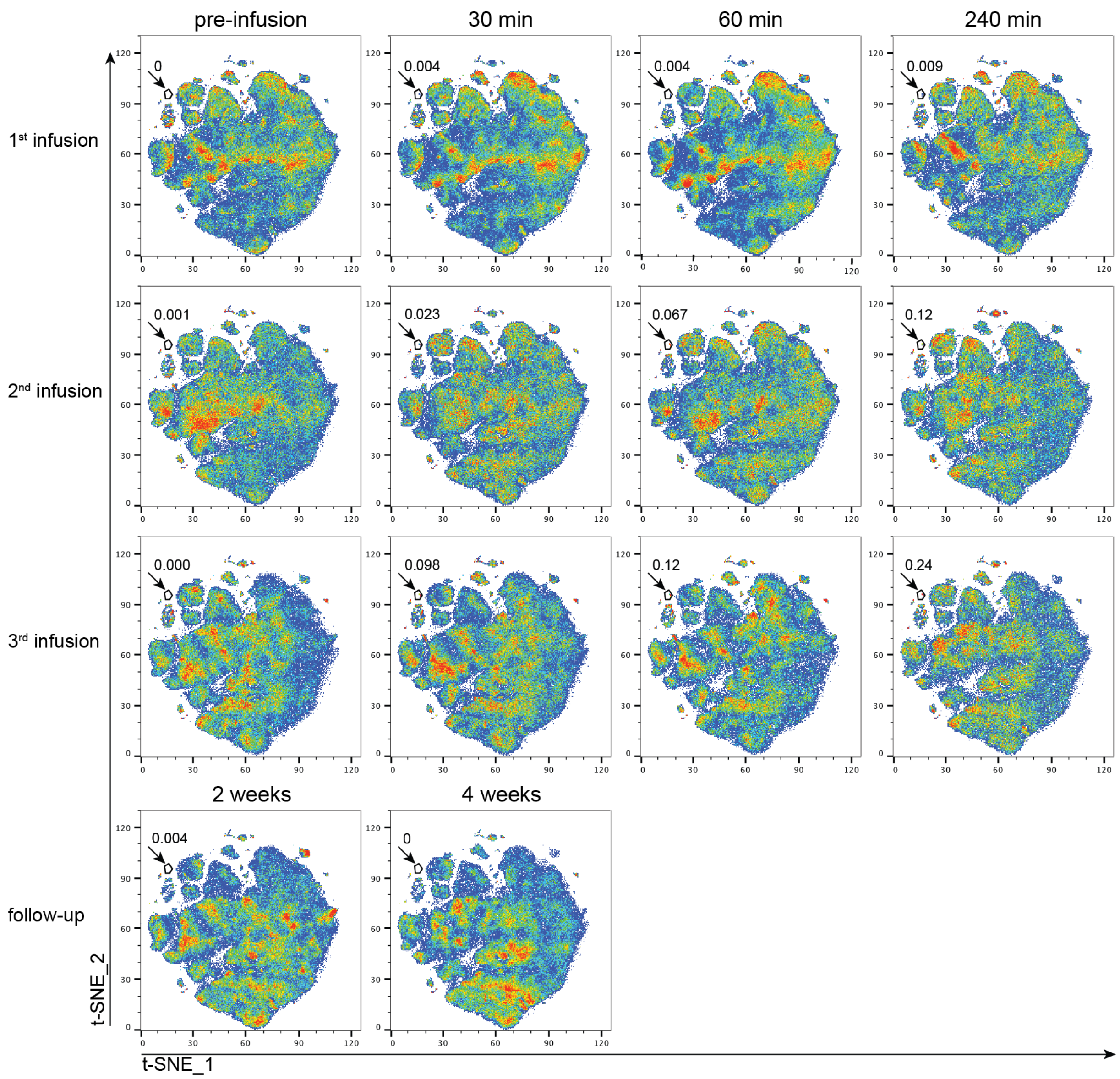

Figure S3. Clustering analysis of data using t-SNE based on 19 markers. Related to Figure 2. Data from $CD56^{+}CD3^{-}CD19^{-}CD14^{-}$ NK cells from all time points were pooled for the calculation. Representative data from one study subject (P103) is shown. The numbers next to the gate represent the percentage of the population within total NK cells at the respective time point.

Figure S4

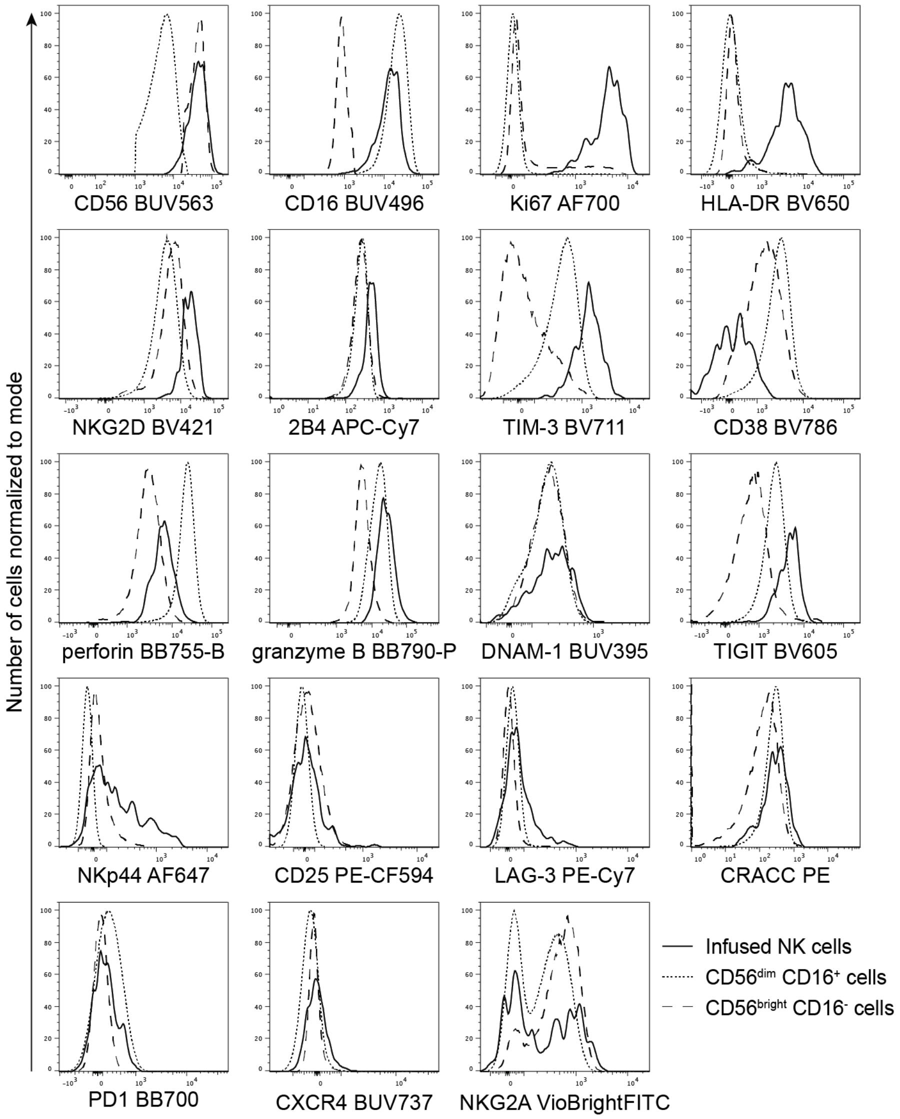

Figure S4. Detailed phenotypic analysis of infused NK cells in comparison to CD56^{dim}CD16^{+} and CD56^{bright}CD16^{-} NK cells in circulation. Related to Figure 2. Representative data from one study subject (P106) is shown. Histograms display data from the respective subpopulations within CD56^{+}CD3^{-}CD19^{-}CD14^{-} NK cells pooled over all time points.

Figure S5

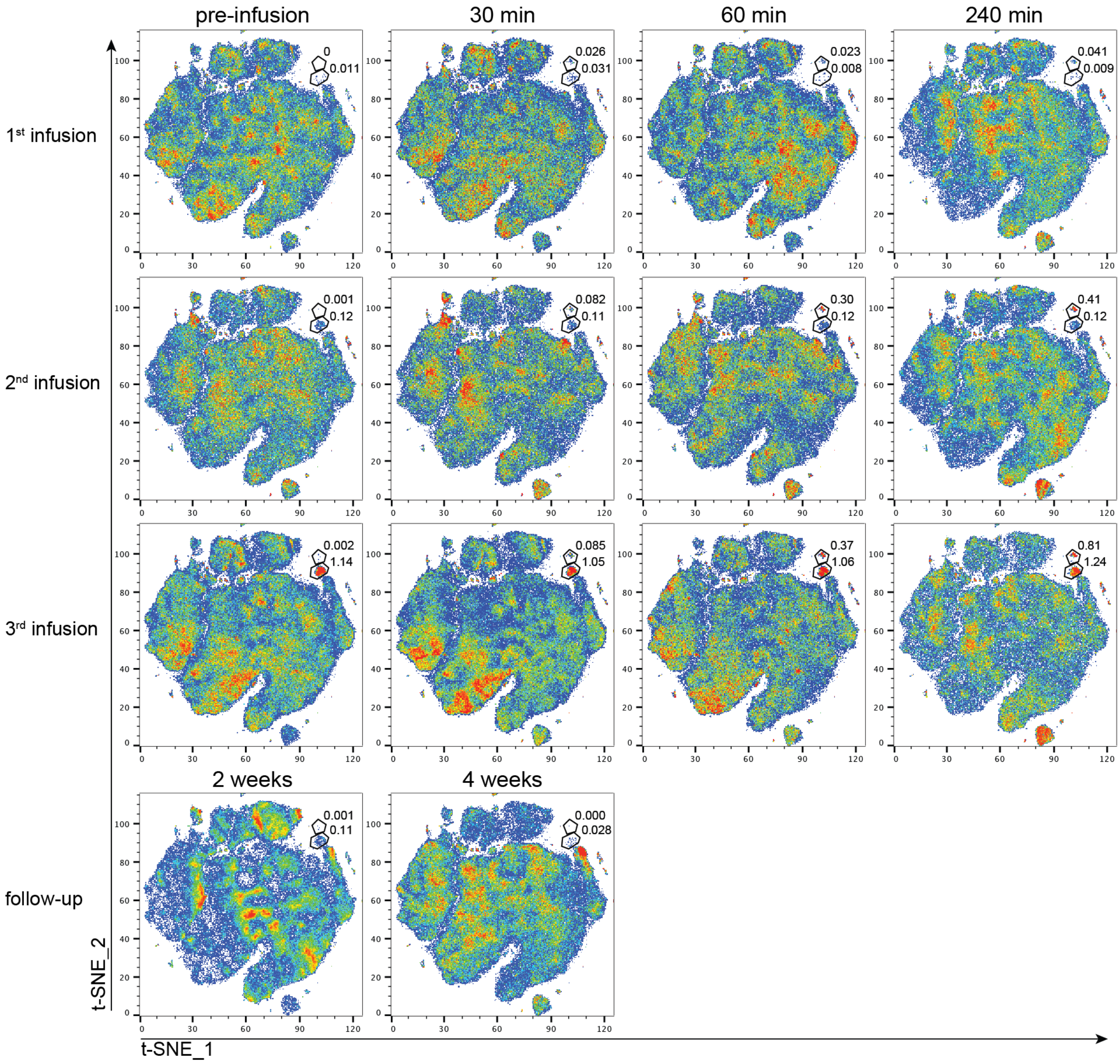

Figure S5. Temporal appearance of infused populations within study subject peripheral blood NK cells. Related to Figure 2. t-SNE plots of data from $CD56^{+}CD3^{-}CD19^{-}CD14^{-}$ NK cells pooled from all time points (study subject P111). The numbers next to the gates represent the percentage of that population within total NK cells at the respective time point.

Supplemental Tables

Table S1. Clinical chemistry at diagnosis and prior to NK cell infusion. Related to Figure 4.

	**At diagnosis**										**Pre-NK cell infusion**				
**Study subject**	**B_2M (mg/L)**	**Albumin (g/L)**	**LDH (ukat/L)**	**Plasma M-spike (g/L)**	**iFLC[a] (mg/L)**	**FLC ratio**	**Urine M-spike (mg/L)**	**Hb (g/L)**	**Creatinine (mmol/L)**		**iFLC[A] (mg/L)**	**FLC ratio**	**Urine M-spike (mg/L)**	**Hb (g/L)**	**Creatinine (mmol/L)**
**P103**	7.1	27	2.3	67	96	9.70	92	96	60		0	0.93	<7.0	94	55
**P105**	4.2	37	2.6	40	58	9.14	33	97	92		4.4	2.90	<7.0	92	66
**P106**	2.4	35	3.4	33	793	193	9.37	127	92		0	0.72	<7.0	136	86
**P107**	-	42	3.9	0	4943	739	5640	95	202		2	1.64	<7.0	116	124
**P110**	3.0	37	4.1	18	1131	126	696	134	78		0	1.03	<7.0	138	81
**P111**	2.5	39	2.2	3[b]	2194	141	1990	115	60		2	1.20	<7.0	111	57

[a] Normal range of FLC-k 6.8-22.4 mg/L

[b] IgD is quantified qualitative, amount may be an underestimation of actual amount

Table S2. **MRD analysis.** Related to Figure 4.

	Read frequency (%)[a]		
**Study subject**	**Diagnosis**	**-2 weeks**	**4 weeks**
**P103**	58.28	0.56	0.25
**P105**	n.c.	n.c.	n.c.
**P106**	54.01	0.033	0.023
**P107**	88.56	b.d.	b.d.
**P110**	n.d.	n.d.	n.d.
**P111**	43.49	0.086	0.052

[a] The read frequency given represents the percentage of the clonal IGH VDJ sequence (identified in the MM diagnosis sample) out of total IGH VDJ sequences.

n.c. no consent to bone marrow sampling

b.d. below detection level; i.e., the frequency of clonal IGH VDJ rearrangements was <1/100 000

n.d. not done

Table S3. Additional study subject information, NK cell dosing, and response status during follow-up.

Related to Figure 4.

Study subject	Response status after ASCT[a]	Time from ASCT to infusions (weeks)	NK cell product doses (10^6 cells/kg)	Response status during six months follow-up	Time from inclusion to progression (months)
**P103**	VGPR	6; 7; 8	5; 50; 100	VGPR	27
**P105**	VGPR	12; 13; 14	5; 50; 100	Relapse at 5 months	15
**P106**	CR	15; 16; 17	5; 50; 52[b]	CR	30
**P107**	VGPR	15; 16; 18	5; 50; 100	CR	>59[c]
**P110**	CR	14; 15; 16	5; 50; 40[b]	CR	>50[c]
**P111**	CR	17; 18; 19	5; 50; 69[b]	CR	38

VGPR, very good partial response; CR, complete remission

[a] ASCT: autologous hematopoietic stem cell transplantation

[b] The third dose was reduced due to scarcity of cells

[c] Study subjects have not progressed at the time of submission

Table S4. **Treatment-emergent adverse events (TEAE).** Related to Figure 5.

Study subject P103

ICD10	TEAE	Manifestation time (days from respective infusion)	Duration (days)	Severity	Causality
R53.9	Malaise and Fatigue	Inf1+0	<1	mild	probable
R11.9A	Nausea	Inf1+0	<1	mild	unlikely
R51.9	Headache	Inf1+1	<1	mild	unlikely
M79.1	Myalgia, legs	Inf1+1	34	mild	possible
M79.1G	Myalgia, legs	Inf1+1	34	moderate	unlikely
M54.2	Neck pain	Inf1+6	29	mild	unlikely
R53.9	Malaise and Fatigue	Inf2+0	<1	mild	unlikely
R53.9	Hot flash	Inf2+0	1	mild	unlikely
J30.4	Rhinitis	Inf2+0	6	mild	unlikely
R53.9	Sickness sensation	Inf3+0	<1	mild	unlikely
R20.2	Paresthesia in toes	Inf3+0	<1	mild	unlikely
R51.9	Headache	Inf3+0	1	mild	unlikely
F32.0	Depression	Inf3+14	n.a.	moderate	unlikely
M54.5	Lumbago	Inf3+78	n.a.	mild	unlikely
B02.9	Shingles, back	Inf3+124	21	moderate	possible
M54.4	Lumbago	Inf3+124	58	mild	unlikely
R50.9	Fever	Inf3+129	1	mild	unlikely
B02.9	Shingles in the groin	Inf3+159	7	moderate	possible

Study subject P105

ICD10	TEAE	Manifestation time (days from respective infusion)	Duration (days)	Severity	Causality
D64.9	Anemia	Inf2+0	1	mild	unlikely
B02.9	Shingles	Inf3+30	5	moderate	possibly
J06.9	Upper respiratory Infection	Inf3+48	16	mild	unlikely
H04.1	Dry eyes	Inf3+90	n.a.	mild	unlikely
J06.9	Upper respiratory infection	Inf3+128	18	moderate	unlikely
D64.9	Anemia	Inf3+146	6	mild	unlikely

Study subject P106

ICD10	TEAE	Manifestation time (days from respective infusion)	Duration (days)	Severity	Causality
R49.0	Dysphonia	Inf1+3	<1	mild	unlikely
R49.0	Dysphonia	Inf1+3	17	mild	unlikely
B02.9	Shingles	Inf3+2	25	moderate	probable
J06.9	Upper respiratory infection	Inf3+56	7	mild	unlikely
M54.5	Lumbago	Inf3+143	3	mild	unlikely

Study subject P107

ICD10	TEAE	Manifestation time (days from respective infusion)	Duration (days)	Severity	Causality
B02.9	Shingles	Inf2+0	13	moderate	possible
K59.1	Diarrhea	Inf2+15	2	mild	unlikely
M54.5	Lumbago	Inf3+6	9	mild	unlikely
K59.1	Diarrhea	Inf3+28	29	mild	unlikely
R20.8	Paresthesia	Inf3+143	40	mild	unlikely

Study subject P110

ICD10	TEAE	Manifestation time (days from respective infusion)	Duration (days)	Severity	Causality
R51.9	Headache	Inf3+1	<1	mild	unlikely
J06.9	Upper respiratory infection	Inf3+	n.a.	mild	unlikely
L57.0	Facial actinic keratosis	Inf3+	n.a.	mild	unlikely
D22.3	Atypical (Dysplastic) melanocytic mole right side of neck	Inf3+	n.a.	mild	unlikely

Study subject P111

ICD10	TEAE	Manifestation time (days from respective infusion)	Duration (days)	Severity	Causality
R25.8	Restless legs	Inf1+0	<1	mild	unlikely
K59.1	Diarrhea	Inf1+6	<1	mild	unlikely
R50.9	Fever	Inf3+4	5	mild	unlikely
R05.9	Cough	Inf3+4	4	mild	unlikely
J06.9	Upper respiratory infection	Inf3+9	20	mild	unlikely
J06.9	Upper respiratory infection	Inf3+53	87	mild	unlikely

Table S5. NK cell product stability. Related to STAR method *Generation of* good manufacturing practice (*GMP) ex vivo activated and expanded NK cells for the clinical study*

	**Dose 1**			**Dose 2**				**Dose 3**	
**Storage time at -180°C (months)**	0	19		0	9	114		0	114
**Viability[a] (%)**	99.0	98.7		99.0	98.2	95.8		99.0	93.5
**NK cells[b] (%)**	84.1	81.5		84.1	83.3	84.4		84.1	84.8
**Degranulation[c] (%)**	80.0	81.0		80.0	76.0	79.1		80.0	84.0

[a] Assessed by trypan blue exclusion

[b] Defined as $CD45^{+}CD56^{+}CD3^{-}$ in flow cytometry

[c] $CD107a^{+}$ NK cells after co-incubation with K562 cell line

Methods S1. Study protocol synopsis, related to STAR methods *Experimental Model and Subject Details*

PROTOCOL SYNOPSIS

**BASICS**	**EudraCT No**<br>2010-022330-83<br>**Project Identifier**<br>ACP-001<br>**Investigational Product**<br>CellProtect; Ex vivo expanded and activated Natural killer (NK) cells<br>**Development phase**<br>Phase I, first-in-human, therapeutic exploratory<br>**Indication**<br>Multiple Myeloma (MM)<br>**Design**<br>Open, single arm, triple dose study<br>**Number of participating investigator sites**<br>One (1)
**ADMINISTRATIVE STRUCTURE**	**Sponsor**<br>Department of Hematology<br>Karolinska University Hospital<br>M54<br>SE-141 86 Stockholm<br>**Manufacturer of the Investigational Product**<br>Vecura<br>Clinical Research Centre<br>Karolinska University Hospital<br>SE-141 86 Stockholm<br>**Principal Investigator**<br>Dr. Hareth Nahi<br>Department of Hematology<br>Karolinska University Hospital<br>M54<br>SE-141 86 Stockholm
**OBJECTIVES**	**Primary objective**<br>• To investigate the safety and tolerability of CellProtect in patients with MM following Autologous Stem Cell Transplantation (ASCT).

Secondary objectives

- To investigate the effect of CellProtect on monoclonal immunoglobulin levels.
- To investigate the effect of CellProtect on free light chain in serum.
- To investigate the effect of CellProtect on plasma cell fraction in bone marrow.
- To investigate the effect of CellProtect on the International Myeloma Working Group uniform response criteria.

POPULATION

Planned number of patients

Considerable withdrawal rate is expected due to the nature of the disease and the following approximate patient recruitment progress is estimated:

20 blood donations
15 commencing study treatment
12 completed

Inclusion criteria

1) Signed Informed Consent
2) 18 to 70 years of age
3) MM, diagnosed according to Greipp PR, San Miguel J, Durie BG, et al. (2005) as having both
   a) Clonal plasma cells in a bone marrow sample
   b) Measurable monoclonal immunoglobulins in plasma or urine
4) Eligible for, and willing to undergo, high dose chemotherapy and ASCT
5) Eastern Cooperative Oncology Group (ECOG) performance status 0-2
6) Life expectancy of at least three months

Exclusion criteria

1. Non-secretory MM
2. Active malignancy, other than MM
3. Blood donation or other significant blood loss within three months from screening
4. Haemoglobin in blood < 80 g/L
5. Any Related Organ or Tissue Impairment (ROTI), as defined by the International Myeloma Working Group (2003), requiring emergency treatment
6. Known or suspected allergic reactions to any ingredient of the IP

7. Diagnosis or indication of any active autoimmune disease, such as Rheumatoid Arthritis, Inflammatory Bowel Disease, Systemic Lupus Erythematosis or Multiple Sclerosis
8. Uncontrolled or severe cardiovascular disease, such as myocardial infarction within six months from screening, heart failure (class III or IV according to New York Heart Association), uncontrolled angina, clinically significant pericardial disease or cardiac amyloidosis
9. Poorly controlled hypertension, defined as blood pressure that remains above goal in spite of the concurrent use of 3 antihypertensive agents of different classes
10. Poorly controlled Diabetes Mellitus, type I or II, defined as screening results for HbA1c of >63 mmol/mol (IFCC)
11. Renal insufficiency manifested by plasma creatinine > 300 µmol/L and/or by the need for dialysis
12. Diagnosis or indication of any clinically relevant hepatic disease, where indication is defined as screening results (plasma) for either
    a. ALAT >1.2 µkat/L (women) and >1.8 µkat/L (men)
    b. ALP >2.8 µkat/L
    c. ASAT >0.92 µkat/L (women) and >1.14 µkat/L (men)
    d. Bilirubin >30 µmol/L
    e. GGT >1.14 µkat/L (women < 41 years), >1.95 µkat/L (women ≥ 41 years), >2.1 µkat/L (men < 41 years) and >3.0 µkat/L (men ≥ 41 years)
13. Clinically relevant ongoing infection, as judged by the investigator
14. Vaccination with any living vaccine within three months from screening
15. Positive for HIV or Hepatitis B/C
16. Known or suspected drug or alcohol abuse, within 12 months from screening
17. Pregnant, trying to become pregnant, or nursing
18. Lack of, or unreliable contraceptive method, as judged by the Investigator
19. Medical history or any abnormal physical finding that is clinically relevant and could interfere with the safety or objectives of the study, as judged by the Investigator
20. Lack of suitability for participation in the trial, for any reason, as judged by the Investigator

	**Withdrawal criteria**<br>• It is the expressed wish of the patient<br>• It is medically necessary, as judged by the Investigator<br>• The Investigational Product (IP) is, or very likely will be, insufficient for at least the first two infusions<br>• The first infusion is not performed within six months from the ASCT<br>• Pregnancy or trying to become pregnant
**STUDY CONDUCT**	**Duration of a patient's participation**<br>Between approximately 12 to 18 months<br>**Number of study visits**<br>Sixteen (16)<br>**Description**<br>After being included in the study, a patient will first donate blood for the production of the IP.<br>Subsequent to the blood donation, the patient will be treated according to current clinical praxis with chemotherapy (typically for two to four months) followed by ASCT.<br>The study treatment should then be initiated within six months from the ASCT, where the time point for first infusion is chosen with consideration to the patient's physical condition; The study treatment cannot start as long as the patient has an unstable or poor condition.<br>When the patient is sufficiently well and stable after the ASCT, the patient will receive three infusions with IP, with an interval of eight days.<br>Safety and efficacy parameters are followed from first infusion until six months from last infusion.<br>**Safety precaution at infusion visits**<br>Any two patients must not be treated on the same day.<br>Furthermore, sequential treatment of patients must be applied until at least two patients have received all three infusions according to the protocol, and the accumulated number of activated NK cells for at least one of the patients adds up to no less than 9.3×10^6 cells/kg body weight. Patients in sequential treatment must be separated by a minimum of six days.<br>Every infusion during the sequential treatment period must be preceded by a safety evaluation, where the Investigator reviews all relevant laboratory analyses data, vital signs, ECG data and AEs for

	previous infusions. The Investigator must consider it safe to proceed before the infusion can take place.<br>Occurrence of any acute reactions are monitored by measuring the body temperature and vital signs 15, 30, 45 (not temperature) minutes and 1, 2, 4, 6 and 24 hours after start of the infusion, as well as ECG recording 6 and 24 hours after start of the infusion. AEs are continuously monitored up until discharge.
**TREATMENTS**	**Description**<br>The IP is a cell suspension based on ex vivo expanded NK cells from patients with MM. The treatment is strictly autologous. The IP is given as three infusions with escalating doses.<br>The cell expansion protocol includes stimulation and selection of NK cells ($CD3^{-}CD56^{+}$), which are expected to be the most cytotoxic to tumour cells.<br>The product is individually prepared and provided in bags, where the volume and concentration of cells depend on the intended dose and the expansion yield. The total volume for each dose level is always within the range of 10 to 200 ml. The contents of each bag are drawn up in a syringe and administered as i.v. infusion.<br>In this study protocol, the expression "investigational product" is synonymous with the expression "Advanced Therapy Investigational Medicinal Product" used in the "Detailed guideline on good clinical practice specific to advanced therapy medicinal products"<br>**Mode of administration**<br>Intravenous infusions.<br>**Dose levels**<br>• First infusion; $5x10^6$ cells/kg body weight<br>• Second infusion; $50x10^6$ cells/kg body weight<br>• Third infusion; $100x10^6$ cells/kg body weight<br>A dose range of ±10 % is acceptable for each dose level. The doses refer to the total number of cells in the preparation before cryopreservation. For a per protocol treatment to be achieved, at least 6 % of the total number of cells should be activated NK cells.<br>If there is a scarcity of material only two infusions may be given. The third infusion should only be given if the available dose is equal or higher than that of the second infusion.
**ASSESSMENTS**	**Safety**<br>• Weight<br>• Physical examination<br>• Vital signs

	• Body temperature<br>• ECG<br>• Laboratory analyses, including:<br>o Standard routine safety analyses of blood and urine<br>o Cytokines IL-2, IL-6, IL-10 and TNF-α in blood<br>• Adverse Events<br>**Efficacy**<br>• The International Myeloma Working Group uniform response criteria<br>• Laboratory analyses, including:<br>o Monoclonal immunoglobulin levels in serum and urine<br>o Free light chain in serum<br>o NK cell count, NK phenotype and plasma cell fraction in bone marrow<br>• Blood and bone marrow samples from consenting patients are saved for future explorative analyses
**STATISTICS**	The sample size of 12 completed patients is based on clinical praxis where sample sizes between 6 to 12 subjects are commonly used for similar safety studies.<br>The study is analysed by descriptive statistics only. Continuous variables are described by summary statistics, i.e. number of observations, mean, standard deviations, medians and range (minimum and maximum values). Categorical variables are summarised in frequency tables as counts and percentages. Graphs are generated when appropriate.<br>Baseline is defined as the status at pre-dose first infusion.
**PROTOCOL**	Version 9.0, dated 03DEC2012
